# Occurrence of antibacterials, antivirals, and anti-inflammatory pharmaceuticals for COVID-19 treatment as emerging contaminants in the Chinese freshwater environment before, during and after the pandemic: the need for dynamic eco-pharmacovigilance

**DOI:** 10.1265/ehpm.25-00395

**Published:** 2026-07-03

**Authors:** Sijia Ma, Hongxia Chen, Jun Wang

**Affiliations:** Institute of Pharmaceutical Innovation, Hubei Province Key Laboratory of Occupational Hazard Identification and Control, School of Medicine, Wuhan University of Science and Technology, Wuhan 430065, China

**Keywords:** Pharmaceuticals in the environment, Emerging contaminants, Anti-COVID-19 drugs, Aquatic environment, Eco-pharmacovigilance

## Abstract

**Background:**

Global epidemic diseases such as COVID-19 have driven both the excessive use of pharmaceuticals and shifts in their usage spectrum. Consequently, the profile of pharmaceuticals in the environment (PiE) may undergo dynamic temporal changes, posing a significant challenge to eco-pharmacovigilance (EPV), a specialized branch of pharmacovigilance focused on detecting, evaluating, understanding, and preventing the adverse environmental effects of pharmaceuticals.

**Methods:**

Based on data extracted from 89 studies published between 2014 and 2025, this review conducts a focused comparison of the occurrence patterns of 43 selected typical anti-COVID-19 drugs (20 antibacterials, 11 antivirals, and 12 anti-inflammatory pharmaceuticals) as contaminants in distinct regions across China’s seven major river basins before, during, and after the pandemic. The need of dynamic EPV was then analyzed.

**Results:**

Before the COVID-19 outbreak, erythromycin, ampicillin, roxithromycin, and acetaminophen dominated the PiE profile in terms of reported maximum residual concentrations; during the pandemic lockdown, ciprofloxacin, ofloxacin, azithromycin, and ketoprofen were identified as the top-priority anti-COVID-19 PiE; after the pandemic, azithromycin, norfloxacin, ofloxacin, ciprofloxacin, and roxithromycin remained the dominant PiE. Residual levels of individual PiE at the same location varied noticeably across the three periods.

**Conclusions:**

Results revealed highly distinct regional and temporal variations in surface freshwater pollution by these COVID-19-associated PiE across the three periods, with no consistent regularity observed, thereby further highlighting the necessity of implementing EPV in a “dynamic” manner. Drawing on the framework of dynamic pharmacovigilance, the implementation of dynamic EPV can be promoted by establishing a dynamic watch-list mechanism, clarifying stakeholder roles, and advancing supportive policies and technological platforms, so as to enable adaptive interventions for the timely management of event-driven pharmaceutical pollution.

**Supplementary information:**

The online version contains supplementary material available at https://doi.org/10.1265/ehpm.25-00395.

## 1. Introduction

The global rise in medical product consumption has elevated the environmental burden of pharmaceuticals, which represent a prominent class of emerging contaminants capable of inducing adverse effects on ecosystems [1–3]. Pharmaceuticals in the environment (PiE) consist of a diverse array of active substances with complex chemical and biological properties [4]. Especially during the coronavirus disease 2019 (COVID-19) pandemic, public health authorities worldwide recommended numerous medications, including antibacterials, antivirals, and anti-inflammatory agents, for the management of severe acute respiratory syndrome coronavirus 2 (SARS-CoV-2) infection and associated bacterial co-infections [5–7]. Accordingly, the consumption of these pharmaceuticals increased tremendously during the pandemic, often even being excessive and inappropriate due to the lack of established treatment protocols at that time [8–11]. Antiviral agents such as favipiravir, lopinavir, oseltamivir, remdesivir, ribavirin, ritonavir, and umifenovir emerged as standard treatments for COVID-19 [12, 13]. In Jordan, antiviral use increased by 37.7% in 2020 compared with 2019 [10]. A time-series analysis based on a dataset of global monthly antimicrobial purchases revealed that worldwide antiviral consumption rose from 54.8 units per 1,000 population in March 2019 (pre-pandemic) to 96.5 units per 1,000 population in March 2020 (during the pandemic), representing the highest increase rate (76.2%) among all subgroups of antimicrobials, including antibacterials, antivirals, and antifungals [14]. Moreover, bacterial co-infection occurred in approximately 16% of COVID-19 patients, leading to the administration of antibacterials (e.g., azithromycin, ceftriaxone, meropenem, moxifloxacin, tazobactam) in over 78% of cases [8]. Global antibacterial purchases increased from 634.9 to 674.1 units per 1,000 population in March 2020 compared with March 2019 [14]. In addition, anti-inflammatory agents, including nonsteroidal anti-inflammatory drugs (NSAIDs) and corticosteroids, were widely used to alleviate fever, pain, and other COVID-19-related inflammatory symptoms [15]. As low-cost, readily available over-the-counter medications, NSAIDs such as aspirin, ibuprofen, and paracetamol were among the most commonly used agents during the pandemic, with reported usage in 19% of SARS-CoV-2-infected individuals [16]. Following the release of World Health Organization (WHO) guidance on corticosteroid use for COVID-19 [17], potent anti-inflammatory corticosteroids such as dexamethasone, prednisone, and hydrocortisone were extensively prescribed to hospitalized patients, even in an inappropriate manner [18].

Such extensive consumption of anti-COVID-19 drugs has further exacerbated the environmental input of these groups of PiE [19–21]. Following administration, pharmaceuticals are excreted via urine and feces as unchanged parent compounds or metabolites, which then enter domestic wastewater systems. Moreover, widespread home storage of medications during the pandemic also contributed to the improper disposal of expired or unused drugs, which may enter groundwater and surface water via landfill leachate [22, 23]. Emissions from hospitals and pharmaceutical manufacturing facilities represent additional important sources. As typical emerging contaminants, PiE are largely unregulated and poorly removed by conventional wastewater and solid-waste treatment systems, resulting in their continuous release and pseudo-persistence in the environment [3]. Pharmaceuticals released into aquatic systems inevitably undergo various natural attenuation processes that can reduce their residual concentrations, including degradation pathways such as photolysis [24], biodegradation [25], and sedimentation [26]. Because many PiE exhibit toxicity toward microorganisms, biodegradation may be less efficient than photodegradation for certain PiE compounds [27]. During photodegradation, a variety of reactive intermediates can be generated, which further promote the degradation of PiE [27, 28]. Despite these natural attenuation processes, the large-scale consumption of anti-COVID-19 drugs during the pandemic has still led to the accumulation of PiE in aquatic environments at relatively high concentrations.

To date, numerous antibacterial, antiviral, and anti-inflammatory agents have been demonstrated to elicit ecotoxicological effects at environmentally relevant concentrations. Previous reviews [29, 30] have indicated that the long-term presence of antiviral drugs (e.g. oseltamivir, amantadine) in aquatic systems, even at low concentrations, may threaten non-target organisms and promote the emergence and spread of environmentally acquired antiviral resistance. Commonly used antibacterials (e.g. fluoroquinolones, macrolides) and their mixtures in aquatic environments can drive the development and dissemination of antibiotic-resistant bacteria and resistance genes, while exerting toxic effects on algae, invertebrates, vertebrates, and other aquatic biota. Additionally, residual antibacterials in ecosystems may also pose a threat to human health through dietary exposure to drug-contaminated plant- or animal-derived food [31–34]. In fact, the WHO has formally recognized the severe ecological risks posed by antibiotic residues [34]. Furthermore, conclusive evidence has been provided regarding the adverse impacts of NSAIDs in the environment on scavenging birds and aquatic species [35–37]. As early as 2011, certain NSAIDs (diclofenac, naproxen and ibuprofen) were included in the European Union (EU)’s first priority list of hazardous PiE for aquatic monitoring [35]. Environmentally relevant concentrations of NSAIDs can induce genotoxicity, oxidative stress, hematological disorders, immunosuppression, and histopathological damage in the intestine, liver, kidney, and gills of fish [35]. Paracetamol, diclofenac, ibuprofen, and other NSAIDs have also been demonstrated to exert adverse effects at the molecular, biochemical, and cellular levels in freshwater invertebrates [36]. Moreover, accumulative evidence suggests that anti-inflammatory corticosteroids represent a group of PiE with high potency and tissue penetration, exerting a series of harmful effects on aquatic ecosystems [38–42]. Chronic exposure to trace levels (ng/L to µg/L) of anti-inflammatory corticosteroids, such as dexamethasone, prednisolone, clobetasol propionate, has been associated with reproductive impairment, behavioral changes, immunosuppression, and osteoporosis-like symptoms in fish, reduced growth and fecundity in *Ceriodaphnia dubia*, and developmental disorders in molluscs. Reported lowest observed effect concentrations (LOECs) in zebrafish were 91 ng/L for clobetasol propionate, 100 ng/L for prednisolone, and 145 ng/L for cortisol [38]. Exposure to 20–60 ng/L dexamethasone for even a short term induced oxidative damage, embryonic alterations, growth and developmental abnormalities in *Cyprinus carpio* embryos [40]. Multi-generational exposure to dexamethasone and prednisolone can alter life-history parameters in *C. dubia*, which may lead to cumulative damage at the population level [39].

Considering the aforementioned ecotoxic effects of anti-COVID-19 drugs, dynamic monitoring of their environmental occurrence has attracted considerable attention to characterize PiE changes before, during, and after the pandemic, thereby facilitating targeted responses to associated ecological threats [20, 43–46]. However, environmental occurrence data for anti-COVID-19 drugs, particularly post-pandemic data, remain fragmented. Most previous studies [20, 43–46] have focused on comprehensive analyses of multiple classes of COVID-19-associated emerging contaminants (e.g., microplastics, disinfectants, antidepressants) using global datasets, which limits the ability to fittingly compare PiE residual patterns across different spatial scales. A focused comparison of occurrence patterns of commonly approved anti-COVID-19 drugs (i.e., antivirals, antibacterials, and anti-inflammatory pharmaceuticals) as PiE within specific regions is therefore imperative. Such targeted analyses will provide more accurate spatiotemporal insights into the pandemic’s impact on PiE profiles, thereby enabling the development of tailored management strategies.

As a source-control strategy for PiE, eco-pharmacovigilance (EPV) is widely recognized for detecting, evaluating, understanding, and preventing the environmental adverse effects of pharmaceuticals from a drug administration perspective [3, 30, 47–49]. EPV emphasizes rigorous environmental monitoring and risk assessment of PiE, supporting the implementation of upstream measures to mitigate pharmaceutical pollution. These measures include regulating emissions from pharmaceutical manufacturing, promoting the rational clinical use of drugs through eco-directed sustainable prescribing, establishing medication take-back and green disposal systems, and developing green drug design and manufacturing processes, *etc.*, all aimed at minimizing the discharge of hazardous PiE at the source [3, 30, 47–49]. To ensure the efficacy of these upstream EPV measures, real-time data on the environmental concentration and sources of PiE are essential for adapting solutions in a targeted and dynamic manner [21, 30]. In response to the COVID-19-associated increase in environmental PiE loads, a previous study [21] has proposed targeting COVID-19-related pharmaceuticals in post-pandemic EPV programs. Thus, the dynamic changes in PiE profiles across different pandemic periods (pre-, during, and post-pandemic) highlight the need for a “dynamic” EPV approach that can adapt to evolving contamination patterns.

Following human consumption, anti-COVID-19 drugs enter the environment primarily via domestic wastewater and surface runoff [19–21], and thus initially accumulate in freshwater environments (i.e., rivers, streams, lakes, and reservoirs). Moreover, freshwater ecosystems are closely associated with human activities and drinking water sources, and are ecologically vulnerable [50]. In this study, we focused on China’s freshwater environment, summarizing occurrence data for four main classes of anti-COVID-19 drugs (antivirals, antibacterials, NSAIDs, and anti-inflammatory corticosteroids) and conducting a time-series comparison of measurements from different regions. The findings contribute to understanding the impact of major epidemic diseases (e.g. COVID-19) on the environmental occurrence of related PiE. Furthermore, we discuss the necessity of implementing dynamic EPV to proactively address emerging environmental challenges posed by these anti-COVID-19 drugs.

## 2. Occurrence analysis of four classes of anti-COVID-19 drugs in Chinese freshwater environment

This section aimed to conduct a time-series comparison of the pandemic’s impacts on the occurrence of major anti-COVID-19 drugs, covering the pre-pandemic, lockdown, and post-pandemic periods. As the initial epicenter of the COVID-19 pandemic, China experienced a relatively prolonged epidemic response and was among the earliest regions with large-scale use of anti-COVID-19 medications [51]. Here, we collected and analyzed the literature published in English between 2014 and 2025 regarding the occurrence of four categories of anti-COVID-19 drugs in Chinese freshwater environments. To support hazard screening and priority-setting for EPV, this review focused on the maximum residual concentrations of PiE, as these values reflect worst-case exposure scenarios relevant to ecological risk assessment.

A previous review by Dai et al. [52] summarized anti-COVID-19 drugs recommended in national COVID-19 clinical guidelines across nine countries, including the United States, the United Kingdom, Italy, Germany, Brazil, Turkey, Korea, India, and China. The present study selected antivirals, NSAIDs, and anti-inflammatory corticosteroids recommended for COVID-19 treatment and symptom relief, identified in that review [52], as target PiE: 11 antiviral drugs: Nirmatrelvir (NIR), Ritonavir (RTV), Molnupiravir (MOL), Remdesivir (RDV), Ribavirin (RBV), Lopinavir (LPV), Oseltamivir (OTV), Ivermectin (IVE), Darunavir (DRV), Favipravir (FPV), Azvudine (AZV); 6 NSAIDs: Ibuprofen (IBF), Diclofenac (DFC), Ketoprofen (KPF), Acetaminophen (ATP), Naproxen (NPX), Indomethacin (IM); 6 anti-inflammatory corticosteroids: Dexamethasone (DXM), Prednisone (PN), Prednisolone (PNL), Hydrocortisone (HYD), Methylprednisolone (MP), Budesonide (BUD).

For antibacterials, only azithromycin (AZM) was officially recommended for COVID-19 inpatients in Italy, as well as for symptomatic patients and pediatric patients in Turkey [52]. However, broad-spectrum antibacterials were widely prescribed in clinical practice to manage bacterial co-infections during the pandemic [53–56]. A meta-analysis of global antibacterial prescriptions for COVID-19 patients [54] and a nationwide analysis of antibacterial procurement in Chinese public health institutions [55] consistently identified the most commonly used antibacterial categories: quinolones (QNs), macrolides (MLs), β-lactams (β-Ls, mainly second- and third-generation cephalosporins), and lincosamides (LCMs). Accordingly, 20 representative antibacterials were included: 6 QNs: Ofloxacin (OFX), Levofloxacin (LVX), Norfloxacin (NOR), Moxifloxacin (MOX), Ciprofloxacin (CPFX), Enrofloxacin (ENR); 4 MLs: Erythromycin (ERY), Roxithromycin (ROX), Clarithromycin (CLR), Azithromycin (AZM); 8 β-Ls: Cefotaxime (CTX), Cefixime (CFM), Cefaclor (CEC), Ceftazidime (CAZ), Ceftriaxone (CRO), Cefuroxime (CXM), Ampicillin (AMP), Amoxicillin (AMX); and 2 LCMs: Lincomycin (LIN), Clindamycin (CLI).

**Fig. 1:**
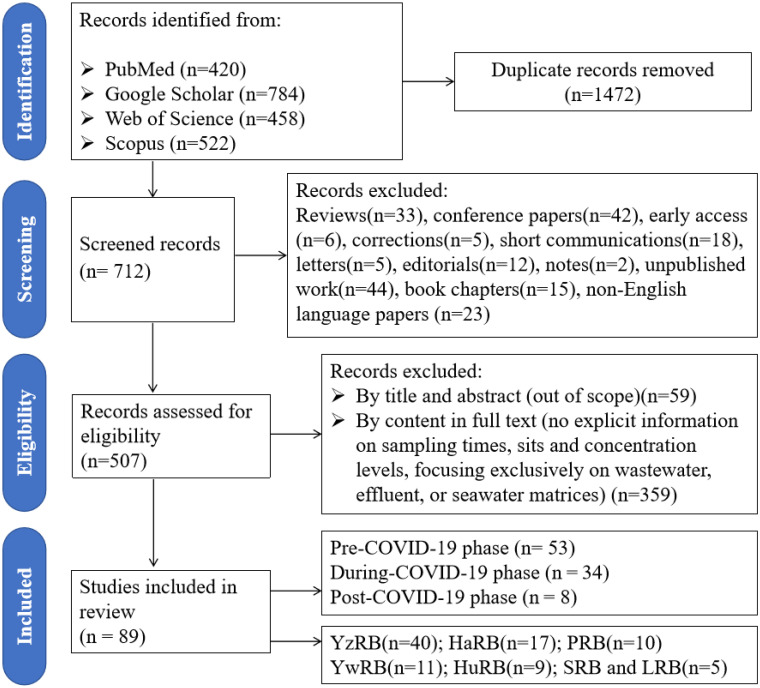
PRISMA-based flowchart of literature screening process.

A bibliographic search was conducted in academic databases, including PubMed, Google Scholar, Web of Science, and Scopus, using combinations of the following keywords: “water environment” OR “aquatic environment”, “China” OR “Chinese”, and the full name of each target drug. The inclusion criteria were as follows: (1) studies reporting occurrence data of the target anti-COVID-19 drugs in Chinese surface freshwater systems, including rivers, streams, lakes, and reservoirs; (2) clear documentation of sampling date, sampling site, and maximum residual concentration; (3) peer-reviewed original research articles published in English between January 1, 2014 and July 31, 2025. The exclusion criteria were as follows: (1) studies focusing exclusively on wastewater, effluent, or seawater matrices; (2) studies lacking explicit information on sampling times, sits and concentration levels; (3) non-original research publications, such as reviews, conference papers, early access, corrections, short communications, letters, editorials, notes, unpublished work, and book chapters. Ultimately, a total of 89 articles reporting the occurrence of antibacterials, antivirals, and anti-inflammatory drugs used in COVID-19 treatment in the Chinese freshwater environment were included in this review. The literature screening process is illustrated in Fig. 1.

Based on official announcements from China’s National Health Commission, the COVID-19 pandemic was formally identified on January 20, 2020 and declared concluded as a public health emergency on December 9, 2022. According to sampling dates, all included studies were categorized into three distinct temporal groups:

● Pre-COVID-19 phase: January 2014–January 2020 (n = 53)
● During-COVID-19 phase: February 2020–December 2022 (n = 34)
● Post-COVID-19 phase: January 2023–July 2025 (n = 8)

**Fig. 2:**
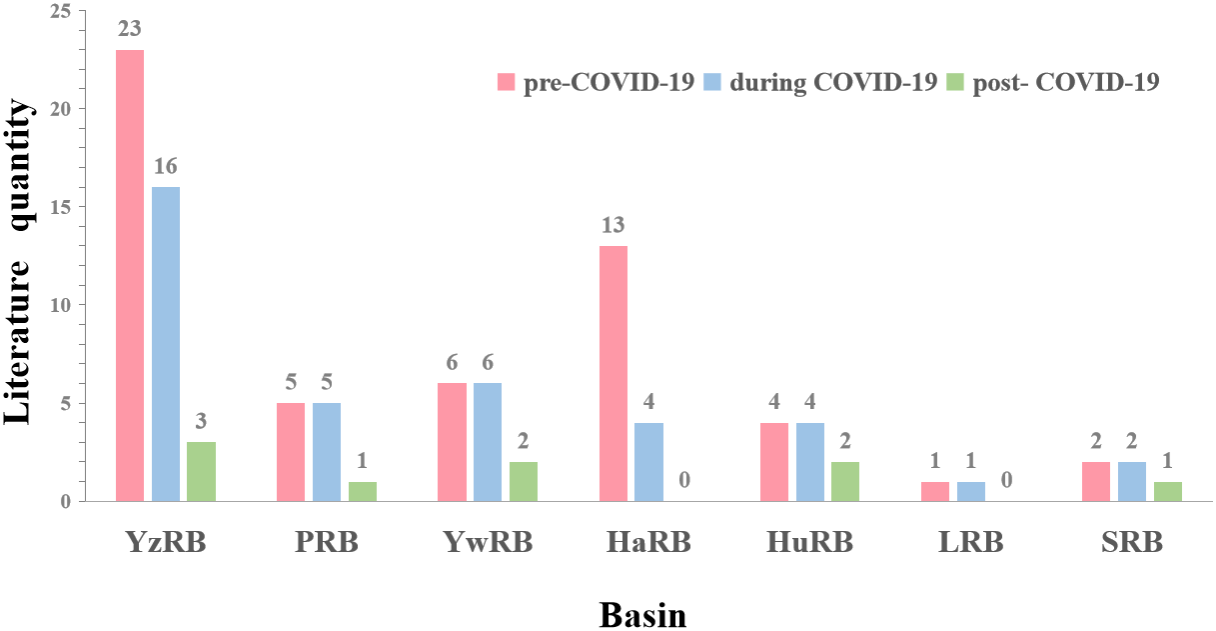
Quantity of included literature reporting occurrence data of anti-COVID-19 PiE in China’s seven major river basins before, during, and after the pandemic.

A detailed summary of the included studies and occurrence data across the three groups is provided in Additional file 1. To characterize the spatialtemporal variations in PiE profiles across China, the occurrence data were stratified according to the nation’s seven major river basins [57, 58]: Yangtze River Basin (YzRB), Yellow River Basin (YwRB), Pearl River Basin (PRB), Hai River Basin (HaRB), Huai River Basin (HuRB), Liao River Basin (LRB), and Songhua River Basin (SRB). As illustrated in Fig. 2, over the past decade, most investigations were conducted in the YzRB (40 studies, 44.9%), followed by the HaRB (17 studies, 19.1%) and YwRB (11 studies, 12.4%). Complete datasets covering all three periods were available for most basins, except for the HaRB and LRB. Detailed analyses were therefore performed for the YzRB, PRB, and YwRB, which had comprehensive temporal coverage and relatively abundant data.

### 2.1. Spatialtemporal variations in the occurrence of anti-COVID-19 drugs in the YzRB

As the third largest river worldwide and the largest aquatic system in China, the Yangtze River runs through 11 provincial administrative regions. The YzRB (90°33′–122°25′E, 24°30′–35°45′N) covers an area of 1,800 × 10^3^ km^2^ and spans key economic zones in western, central and eastern China [59]. Several megacities with populations exceeding 10 million, including Shanghai, Wuhan, and Chongqing, are distributed along its mainstream. The YzRB supports nearly one-third of China’s population, receives approximately 40% of the nation’s wastewater discharge, and supplies drinking water to about 500 million people [60]. Consequently, the continuous input of anthropogenic pollutants has rendered the YzRB one of the primary regions for monitoring emerging contaminants in China [60, 61].

Concentration distributions of target anti-COVID-19 drugs in the YzRB across the three periods are summarized in Table S1. Among the four classes of anti-COVID-19 drugs, only antibacterials were consistently detected in YzRB surface water across pre-, during, and post-COVID-19 periods. In the pre-pandemic period, the macrolide antibiotic ERY exhibited the highest residual level (up to 1,490 ng/L) [62], followed by CTX (830 ng/L) and AMX (710 ng/L) [63]. Notably, the concentrations of these three dominant antibacterials remained below 200 ng/L during and after the pandemic. During the COVID-19 period, CPFX showed the highest concentration (2,717.31 ng/L) [64], followed by CLI (2,406.09 ng/L), NOR (2,178.01 ng/L), and OFX (1,395.9 ng/L) [64, 65], indicating that QNs became the predominant antibacterial contaminants in the YzRB during this phase. In the post-pandemic period, AZM (368.2 ng/L) and ROX (257.56 ng/L) were the two most abundant antibacterials, whereas their maximum pre-pandemic concentrations were only 99 ng/L and 190 ng/L, respectively. In particular, CLR and AZM were consistently below 100 ng/L before the pandemic, but increased sharply to 266 ng/L and 935 ng/L during the pandemic, and remained elevated at 149.54 ng/L and 368.2 ng/L after the pandemic. These data demonstrate a pronounced shift in the antibacterial contamination profile within the YzRB.

**Fig. 3:**
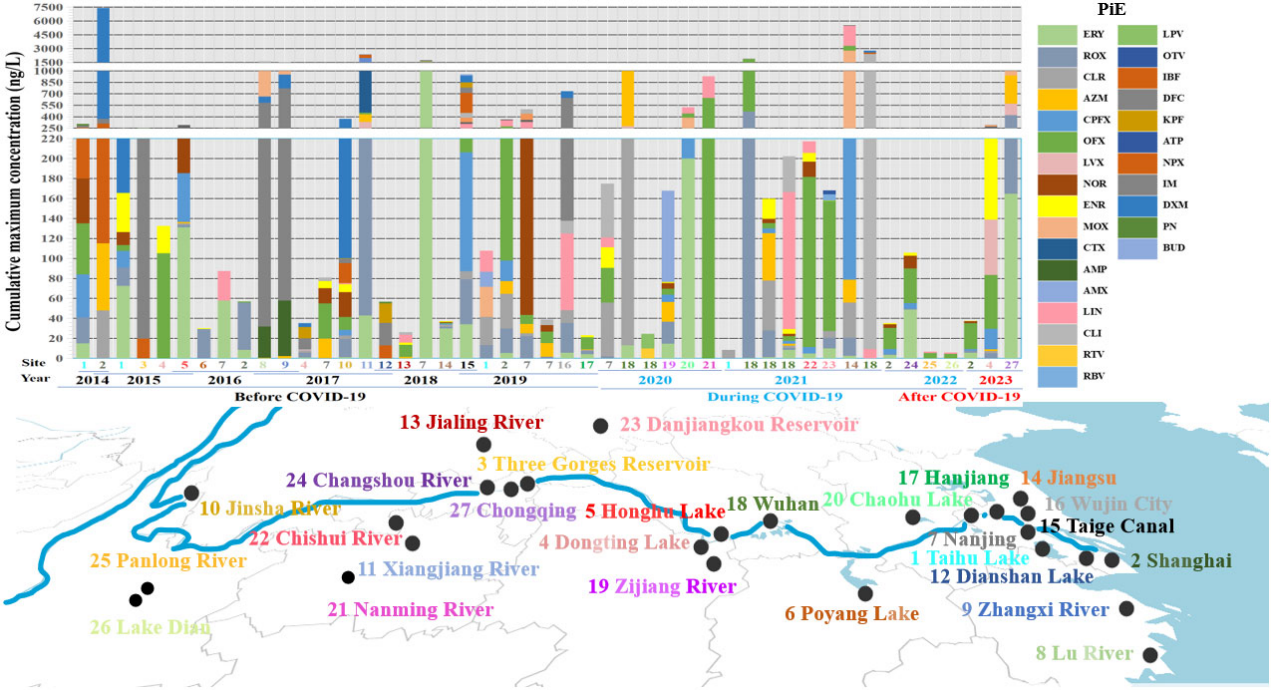
Cumulative maximum residue concentrations of anti-COVID-19 drugs collected at representative sampling point in the YzRB.

Figure 3 shows the cumulative maximum concentrations of anti-COVID-19 drugs at representative sites in the YzRB over the three periods. Results indicated that the residual levels of individual PiE at the same location varied noticeably across periods, reflecting highly dynamic contamination patterns. The highest cumulative concentration (approximately 7,500 ng/L) was recorded in Shanghai in 2014 during the pre-pandemic phase [66]. During the pandemic, the peak cumulative concentration (nearly 6,000 ng/L) occurred in Jiangsu Province [64], with most values ranging from 1,000 to 1,500 ng/L [67]. After the pandemic, cumulative concentrations in Dongting Lake remained above 250 ng/L in 2023, which were generally higher than pre-pandemic levels [68]. In Wuhan, cumulative maximum concentrations were higher in 2021 than in 2020 [65, 69].

### 2.2. Spatialtemporal variations in the occurrence of anti-COVID-19 drugs in PRB

As the second-largest river system in China in terms of water discharge, the Pearl River consists of the North River, West River, East River and the Pearl River Delta, and environmental contaminants entering the PRB eventually discharge into the South China Sea. The PRB covers an area of approximately 453,700 km^2^ and encompasses numerous highly urbanized and industrialize cities [70]. Urban rivers have been recognized as major contributors to pharmaceutical pollution in the main Pearl River channel [71]. To date, the PRB has suffered substantial PiE contamination driven by direct and indirect anthropogenic inputs [70, 72].

**Fig. 4:**
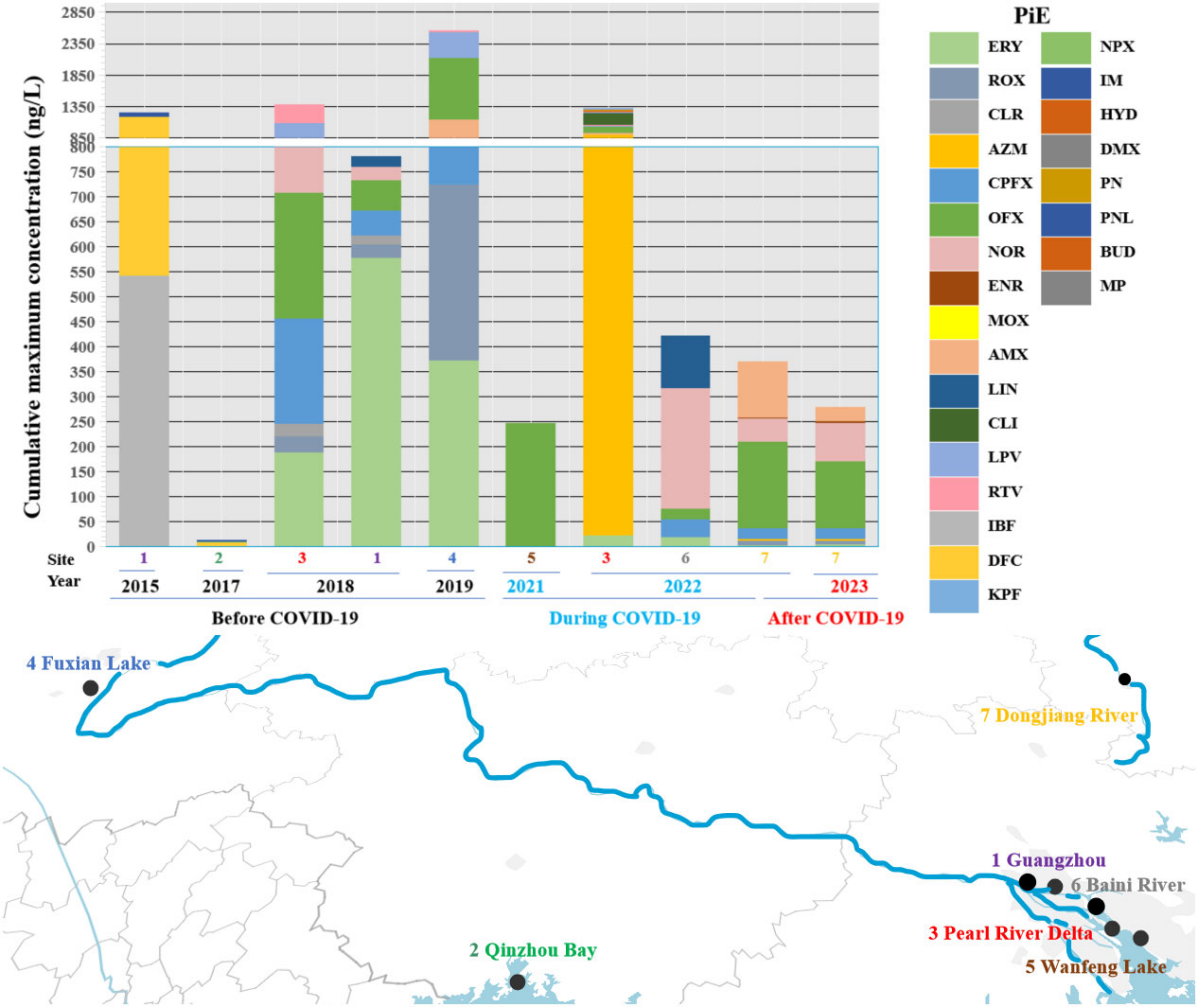
Cumulative maximum residue concentrations of anti-COVID-19 drugs collected at representative sampling point in the PRB.

As summarized in Table S2, ERY, AZM, and OFX were the dominant antibacterial contaminants in PRB surface freshwater before, during, and after the pandemic, with peak concentrations of 577, 877.52, and 133.75 ng/L, respectively. In the pre-pandemic period, ERY was the sole antibacterial detected above 100 ng/L [73]. During the pandemic, multiple antibacterials, including AZM, OFX, NOR, AMX, LIN, and CLI, exhibited maximum concentrations exceeding 100 ng/L. Among these, OFX and NOR showed clear upward trends over time. The maximum concentration of OFX was 60.3 ng/L in 2018 [73], peaked at 246.96 ng/L during the pandemic [74], and remained elevated at 133.75 ng/L in the post-pandemic period [75]. A similar temporal pattern was observed for NOR. Regarding anti-inflammatory corticosteroids, the maximum concentrations of PN, PNL, and MP increased from 2.3, 1.8, and 3.8 ng/L (pre-COVID-19) to 16.15, 5.16, and 6.4 ng/L (during COVID-19), respectively. In contrast, DXM decreased from a pre-pandemic maximum of 3.5 ng/L to 0.66 ng/L during the pandemic. Temporal variations in anti-COVID-19 drug occurrence at representative sampling sites (Fig. 4) further demonstrate pronounced dynamic shifts in the pharmaceutical contamination profile of PRB surface water.

### 2.3. Spatialtemporal variations in the occurrence of anti-COVID-19 drugs in the YwRB

As China’s second-longest river, the Yellow River has a mainstem length of 5,464 km and originates in the westernmost region of the country. It flows eastward through nine provinces in northern China, draining a basin area of 7.95 × 10^5^ km^2^ [76]. The aquatic environment of the YwRB receives continuous pollutant inputs from both point and non-point sources associated with agricultural, industrial, and domestic human activities [77].

**Fig. 5:**
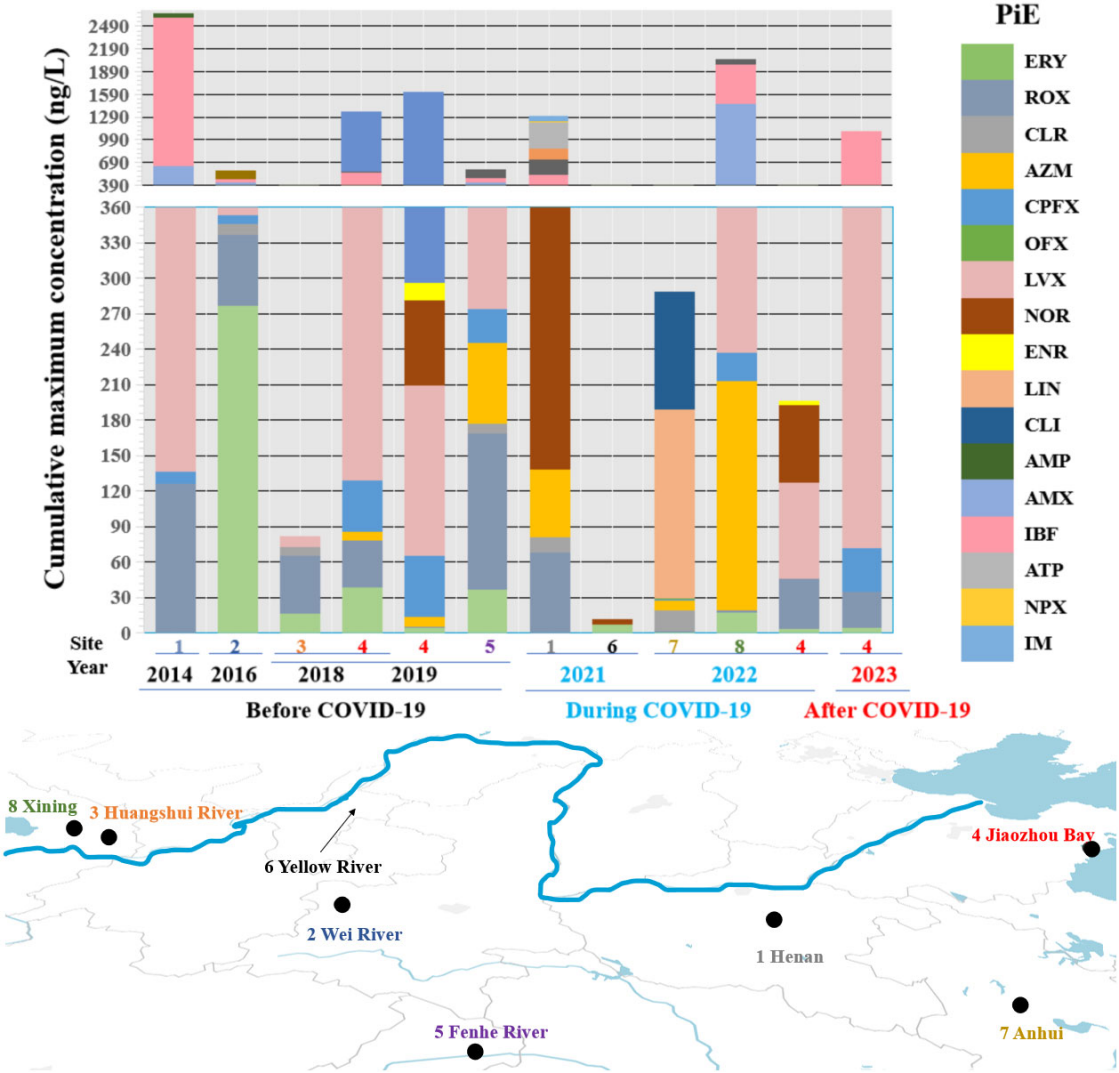
Cumulative maximum residue concentrations of anti-COVID-19 drugs collected at representative sampling point in the YwRB.

In contrast to the YzRB and PRB where ERY was the predominant antibacterial PiE, AMX exhibited the highest residual level (up to 1,334 ng/L) in the YwRB during the pre-COVID-19 period (Table S3) [78]. During the pandemic, OFX became the primary antibacterial contaminant in YwRB freshwater. In freshwater samples collected from the Yellow River source region on the Qinghai-Tibet Plateau in March 2022, OFX concentrations ranged from 0.54 to 1,220.86 ng/L, with a corresponding risk value of 108.04 [79]. This peak OFX concentration during the pandemic represented an approximate 5-fold increase relative to pre-pandemic levels. Only one study reported post-pandemic occurrence of anti-COVID-19 antibacterials in the YwRB, including ERY, ROX, CPFX, OFX, NOR, and ENR [80]. Among these, NOR exhibited the highest residual level of 730 ng/L, followed by OFX (306 ng/L). Notably, this study collected water samples during two periods: November–December 2022 (during the pandemic) and July–August 2023 (post-pandemic) [80]. Maximum concentrations of NOR and OFX increased by approximately 11.1- and 4.5-fold, respectively, from the pandemic to post-pandemic period, which may be attributed to strong adsorption of QNs to particulate matter and their subsequent release from sediment [80]. Occurrence data for antivirals and anti-inflammatory corticosteroids in the YwRB remain limited, while NSAIDs were only detected during the pandemic. Thus, a full temporal comparison for these three classes of PiEs could not be performed in the basin. Figure 5 depicts the cumulative maximum concentrations of target PiE at eight sampling sites across the YwRB, demonstrating clear spatial and temporal differences in contamination profiles among the three periods. The highest cumulative concentration (exceeding 1,900 ng/L) was observed in surface water from Xining urban wetland in 2022 (during the pandemic) [79].

## 3. Implication for dynamic EPV

The occurrence analysis of anti-COVID-19 drugs in the remaining river basins in China is provided in Additional file 3. As summarized in Table 1, although not fully comprehensive, the available monitoring data clearly demonstrate substantial spatio-temporal variations in the profiles of anti-COVID-19 PiE across China’s seven major river basins during the pre-, during-, and post-pandemic periods. Such dramatic shifts in PiE composition, specifically triggered by the COVID-19 public health emergency, pose considerable challenges to the timely and effective implementation of traditional EPV measures.

**Table 1:** Maximum residual concentrations of dominant anti-COVID-19 drugs in surface freshwater samples from seven basins.

**Basin**	**Location**	**Basin’s total length** **(km)**	**Area** **(km^2^)**	**Number of population** **(million)**	**Before the pandemic** **(ng/L)**	**During the pandemic** **(ng/L)**	**After the pandemic** **(ng/L)**
YzRB	Western, central and eastern China	6,363	18 × 10^5^	500	ERY (1,490)	CPFX (2,717.31)	AZM (368.2)
YwRB	Western, central and eastern China	5,464	7.95 × 10^5^	113.68	AMX (1,334)	OFX (1,220.86)	NOR (730)
PRB	Southern China	2,320	4.54 × 10^5^	87.66	ERY (577)	AZM (877.52)	OFX (133.75)
HaRB	Northern China	-	0.32 × 10^5^	28	ATP (3,577)	ERY (201.68)	-
HuRB	Eastern China	1,000	0.27 × 10^5^	190	ROX (566.79)	KPF (1,715.4)	CPFX (1,409)
LRB	Northeast China	1,430	2.3 × 10^5^	4.4	AMX (128.59)	ROX (9.20)	-
SRB	Northeast China	1,927	0.54 × 10^5^	53.53	AMX (134)	CPFX (159.38)	ROX (153.82)

### 3.1. Potential temporal lag between priority PiE identification and source-control implementation in the traditional EPV system

In the EPV practices implemented in leading regions such as Europe, the United States, Japan, and Australia, the identification and listing of potentially hazardous PiE of concern as priority environmental substances, subsequently mandated to undergo Environmental Risk Assessment (ERA) and targeted source control of their discharge, represent the commonly adopted EPV framework [47, 81]. For example, owing to environmental risks identified in the 1990s associated with the veterinary use of diclofenac, a common NSAID, on exposed Gyps vulture populations [82], the EU listed diclofenac as a priority hazardous substance under the Water Framework Directive in 2011 and proposed close monitoring of its environmental concentrations across Europe [83]. Subsequently, an environmental quality standard for diclofenac in European surface water bodies was established and legally enforced at 0.050 µg/L [84]. Since then, multiple EPV measures have been applied in practice to reduce the excessive use of diclofenac at the sources. To date, diclofenac has been banned for veterinary use in India, Nepal, Pakistan, the Europe, the United States, Canada, New Zealand, Australia, Japan, and many other countries [35, 47, 85]. In addition, green alternatives such as meloxicam have been identified and adopted for the treatment of livestock diseases. These EPV measures have been shown to contribute to the effective recovery of Gyps vulture populations [85, 86]. Timely identification of priority PiE enables the prompt optimization of targeted management strategies for upstream anthropogenic activities associated with relevant pharmaceuticals at the source. These strategies include promoting the rational use and appropriate prescription of human and veterinary medicines to prevent misuse or overuse and reduce related emissions, as well as prioritizing targeted interventions and improving supporting regulatory frameworks and policies [81]. However, a time lag typically exists between the identification of high-priority hazardous PiE and the subsequent implementation of source control measures.

To effectively address the risks posed by PiE through EPV strategies, it is essential to account for their dynamic occurrence and exposure patterns, thereby integrating a dynamic mechanism into the traditional EPV system. Targeted EPV measures are often designed and implemented with a certain delay following the identification of priority PiE; thus, close attention should be paid to potential temporal changes in the environmental PiE profile. Given that major epidemics such as COVID-19 can significantly alter the occurrence of PiE, flexible responses are required, with upstream EPV strategies dynamically adjusted based on real-time pharmaceutical pollution assessments.

### 3.2. Insights from dynamic pharmacovigilance for the development of dynamic EPV

As an emerging branch of pharmacovigilance (PV) aimed at monitoring and mitigating the adverse effects of PiE, EPV is considered to benefit from drawing experience from PV development and practice [4, 47, 49]. According to the WHO definition, PV is a scientific discipline within pharmacy administration that encompasses various activities for detecting, assessing, understanding, and preventing adverse drug reactions (ADRs) [87]. The first PV-related study was published in 1974, and over the past two decades, the role and system of PV have developed and expanded rapidly [49, 87]. Especially in developed countries, PV policies and regulations have been established and strictly enforced, and PV has been widely integrated into clinical and pharmaceutical practice [88]. Given the critical role of timely and efficient data collection and processing in ADR management, dynamic PV has been proposed as an optimized reactive framework that emphasizes the real-time detection of PV signals and rapid response to emerging drug safety risks [89–92]. The capacity to effectively capture, analyse, and act on highly heterogenous data in a timely manner is widely recognized as the core of PV [93, 94]. Rapid responses to the growing volume of complex and variable drug safety signals, such as the so-called “PV black swan” events characterized by unpredictability, retrospective bias, and widespread, severe consequences, highlight the urgent need for dynamic PV approaches [93, 94].

As a key element in the operating mechanism of robust PV systems [95], dynamic PV emphasizes the continuous integration of post-marketing information on medicines, enabling up-to-date benefit–risk assessments of approved products [91, 96–99]. In PV practice, the benefit-safety profiles of medicines are dynamic and evolve over time as new ADR signals and related data emerge [100]. Under the EU Guidelines on PV for Medicinal Products for Human Use, a European Risk Management Plan (EU-RMP) must be submitted during the market authorization application for any pharmaceutical product. This plan is characterized by its dynamic nature, which is reflected in the continuous revision of the EU-RMP at any time during the post-authorization period, based on an increasingly comprehensive understanding of the product’s benefit-safety profile derived from new data from post-marketing surveillance or specific post-marketing studies [96]. In recent years, computational approaches have gained prominence due to their flexibility and sensitivity in detecting early ADR signals in PV datasets through temporal data mining, thereby supporting the implementation of dynamic PV [101, 102].

In response to the spatiotemporal dynamic nature of PiE occurrence, evidenced by the changing profiles of anti-COVID-19 drugs in China’s aquatic systems before, during, and after the pandemic, we preliminarily propose here that, based on the experience of dynamic PV, EPV should strive to dynamically and rapidly respond to potential adverse events associated with PiE.

## 4. Limitations and outlook

### 4.1. Limitations of this study

This review did not incorporate Chinese-language databases, which may compromise its comprehensiveness. Furthermore, this study focused on the maximum concentration values of anti-COVID-19 drugs to investigate the spatiotemporal variations in PiE profiles. Although this approach can facilitate the identification of high-priority hazardous PiE, it may overrepresent extreme values. In addition, to facilitate a dedicated spatialtemporal analysis, freshwater environments are specifically targeted in this study. Given that seawater acts as an important final sink for PiEs transported from rivers and wastewater effluents, future studies are strongly encouraged to extend monitoring to coastal and marine systems, to improve the understanding of the full environmental fate of these PiEs, and to consider their dynamic EPV management from broader aquatic perspectives.

Another limitation of this study is the insufficient consideration of COVID-19’s potential impacts on freshwater systems. For instance, previous studies have highlighted that human activities and industrial discharge significantly decreased during the pandemic [103, 104]. Natural factors, such as seasonal climate variability and hydrological conditions, may also influence PiE concentrations in freshwater environment [105]. In particular, it remains unclear whether COVID-19 case numbers are correlated with PiE occurrence in freshwater environments during the pandemic. Further research is needed to identify the specific factors underlying the observed differences in PiE concentrations across river basins.

Moreover, the availability and completeness of post-pandemic data were uneven across regions and periods, which may introduce temporal and spatial biases into the analysis. Heterogeneity existed in monitoring standards, data collection protocols, and analytical methods among different data sources, potentially reducing the comparability of the integrated dataset. Additionally, this research relied heavily on secondary data rather than primary field investigations, which limited our control over variable definitions, sampling strategies, and measurement accuracy. These limitations suggest that future studies should adopt more consistent monitoring frameworks and multi-source data validation to improve robustness and generalizability.

### 4.2. Proposal for a dynamic EPV framework

To better address the spatiotemporally variable occurrence and ecological risks of PiE, we herein propose a dynamic EPV framework that focuses on the continuous and real-time detection, evaluation, understanding, and prevention of adverse effects associated with PiE. This framework aims to tackle the dynamic PiE profile identified in this review. Specifically, the traditional EPV framework, originally developed to detect, evaluate, understand, and prevent the adverse environmental effects of pharmaceuticals at the source, needs to be upgraded to a context-adaptive dynamic implementation mode to provide targeted solutions for managing event-driven pharmaceutical pollution. Drawing on dynamic PV principles and integrating the specific findings of this review, we suggest the following measures for the proposed dynamic EPV framework, which differs from general environmental monitoring principles by incorporating pandemic-induced PiE dynamics:

(1) Establish a region-specific dynamic watch-list mechanism for PiE: A dynamic watch-list mechanism is essential for ongoing environmental monitoring and the early identification of high-priority hazardous PiE in specific areas, ensuring the timely refinement of EPV interventions tailored to meet the unique requirements of the moment. Particularly in light of the impacts of epidemic diseases such as COVID-19 on PiE profile in freshwater environments, as identified in this study, a watch-list mechanism is urgently needed in epidemic epicenter regions, based on the dominance patterns specific to different pandemic phases (e.g., prioritizing CPFX, OFX, and AZM in post-pandemic monitoring, as clarified in this study).

(2) Clarify stakeholder roles with actionable responsibilities: Drawing on PV experience, pharmaceutical manufacturers and hospitals are obligated to conduct timely spontaneous reporting and intensive monitoring of ADRs [92]. Similarly, key stakeholders in EPV practices, including pharmaceutical manufacturers, hospitals, and environmental protection agencies, should make significant contributions to the real-time monitoring of pharmaceutical pollution and emissions [106], thereby providing adequate and timely data to support the rapid identification of priority PiE. For instance, following the outbreak of epidemic diseases such as COVID-19, pharmaceutical manufacturers should be required to report real-time production and distribution data of epidemic-relevant drugs (e.g., antivirals, antibacterials, and anti-inflammatory agents during the COVID-19 pandemic) to environmental protection agencies; hospitals could implement standardized monitoring of pharmaceutical-containing wastewater discharges, particularly for the dominant PiE identified in real time; and environmental protection agencies would coordinate cross-sector data sharing and regularly update monitoring priorities based on PiE dynamics.

(3) Advance policy and technological support tailored to dynamic PiE management: Technological advancements and supportive policies are critical to facilitating the timely utilization of real-world data and maintaining fit-for-purpose EPV measures. At the policy level, adaptive environmental quality standards for dominant PiE need to be developed. Moreover, the PV experience of computer-aided temporal dynamic signal detection of ADRs [101] offers a valuable reference for dynamic EPV. To date, a variety of software platforms and databases have been employed for the rapid prediction and comprehensive assessment of environmental risks posed by PiE [2]. It can be expected that, with further investigation and standardization of computer-aided ERA procedures, dynamic EPV may be gradually put into practice by integrating dynamic mechanisms into the current EPV system.

In essence, EPV is a continuous and dynamic process that requires the ongoing incorporation of real-time data from environmental monitoring and the risk assessment of PiE to effectively reduce pharmaceutical pollution [47, 81, 107]. Nevertheless, further research is needed to precisely determine how to implement dynamic EPV strategies in practice.

## 5. Conclusions

This study comparatively analyzed the occurrence of four categories of anti-COVID-19 drugs (i.e. antivirals, antibacterials, NSAIDs, and corticosteroids) in freshwater environments across China’s seven basins, focusing specifically on temporal variations in PiE profiles before, during, and after the COVID-19 pandemic. Based on data synthesized from 89 studies (2014–2025), this review provides insights advancing current understanding of PiE and EPV as follows: First, the COVID-19 pandemic induced a distinct shift in the PiE profile of Chinese freshwater matrices. Specifically, our analysis indicates that the pre-pandemic PiE profile was dominated by the antibacterials ERY, AMX, ROX, and the NSAID ATP; during the pandemic, CPFX, OFX, AZM, and KPF emerged as the top-priority anti-COVID-19 PiE, reflecting increased clinical use of these antimicrobial and anti-inflammatory agents during the public health emergency; in the post-pandemic period, AZM, NOR, OFX, CPFX, and ROX remained dominant, potentially indicating lingering impacts of pandemic-related pharmaceutical use on freshwater ecosystems. Importantly, this study reveals that existing EPV frameworks lack adaptability to event-driven PiE shifts (e.g., those induced by pandemics), highlighting an urgent need for a dynamic EPV approach that departs from traditional monitoring and source-control paradigms. Drawing on dynamic PV experience, dynamic EPV can be facilitated through establishing a region-specific dynamic PiE watch-list mechanism, clarifying stakeholder roles with actionable responsibilities, and advancing supportive policies and technological innovation tailored to dynamic PiE management. However, despite the observed spatiotemporal variations in PiE across the seven basins, their underlying drivers (e.g., correlation between COVID-19 case numbers and PiE concentrations, interactive effects of human activity changes and natural factors on PiE dynamics) remain unclear, representing a critical knowledge gap that needs to be addressed to further refine the dynamic EPV framework and enhance its practical applicability.

## Abbreviations

PiE: Pharmaceuticals in the environment
EPV: Eco-pharmacovigilance
COVID-19: Coronavirus disease 2019
SARS-CoV-2: Syndrome coronavirus 2
NSAIDs: Nonsteroidal anti-inflammatory drugs
EU: European Union
LOECs: Lowest observed effect concentrations
NIR: Nirmatrelvir
RTV: Ritonavir
MOL: Molnupiravir
RDV: Remdesivir
RBV: Ribavirin
LPV: Lopinavir
OTV: Oseltamivir
IVE: Ivermectin
DVR: Darunavir
FPV: Favipravir
AZV: Azvudine
IBF: Ibuprofen
DFC: Diclofenac
KPF: Ketoprofen
ATP: Acetaminophen
NPX: Naproxen
IM: Indomethacin
DXM: Dexamethasone
PN: Prednisone
PNL: Prednisolone
HYD: Hydrocortisone
MP: Methylprednisolone
BUD: Budesonide
AZM: Azithromycin
QNs: Quinolones
MLs: Macrolides
β-Ls: β-lactams
LCMs: Lincosamides
OFX: Ofloxacin
LVX: Levofloxacin
NOR: Norfloxacin
MOX: Moxifloxacin
CPFX: Ciprofloxacin
ENR: Enrofloxacin
ERY: Erythromycin
ROX: Roxithromycin
CLR: Clarithromycin
CTX: Cefotaxime
CFM: Cefixime
CEC: Cefaclor
CAZ: Ceftazidime
CRO: Ceftriaxone
CXM: Cefuroxime
AMP: Ampicillin
AMX: Amoxicillin
LIN: Lincomycin
CLI: Clindamycin
YzRB: Yangtze River Basin
YwRB: Yellow River Basin
PRB: Pearl River Basin
HaRB: Hai River Basin
HuRB: Huai River Basin
LRB: Liao River Basin
SRB: Songhua River Basin
ND: Not detected
ERA: Environmental Risk Assessment
PV: Pharmacovigilance
ADRs: Adverse drug reactions
EU-RMP: European Risk Management Plan
PRISMA: Preferred Reporting Items for Systematic Reviews and Meta-Analyses

## Supplementary information

**Additional file 1:** Detailed occurrence data of anti-COVID-19 drugs as PiE reported in all the included studies.

**Additional file 2:** **Table S1** Occurrence of anti-COVID-19 drugs in surface freshwater samples collected from the YzRB, China. **Table S2** Occurrence of anti-COVID-19 drugs in surface freshwater samples collected from the PRB, China. **Table S3** Occurrence of anti-COVID-19 drugs in surface freshwater samples collected from the YwRB, China.

**Additional file 3:** Occurrence analysis of four classes of anti-COVID-19 drugs in the HaRB, HuRB, LRB and SRB.

## Declarations

### Availability of data and material

Data will be made available on request.
