# Supplementary material for: Occurrence of antibacterials, antivirals, and anti-inflammatory pharmaceuticals for COVID-19 treatment as emerging contaminants in the Chinese freshwater environment before, during and after the pandemic: the need for dynamic eco-pharmacovigilance
Source: Environ Health Prev Med. 2026 Jul 3;31:44. doi: 10.1265/ehpm.25-00395 (PMC13366171; doi:10.1265/ehpm.25-00395)
Supplement: Supplementary file 1 — Additional file 1: Detailed occurrence data of anti-COVID-19 drugs as PiE reported in all the included studies. [file ehpm-31-044-s001.docx]

**Additional file 1:** Detailed occurrence data of anti-COVID-19 drugs as PiE reported in all the included studies [1-89].

| **Group** | **Sampling sites** | | **Sampling time** | **Studied anti-COVID-19 PiE** | | **Range**  **(ng/L)** | **Mean**  **(ng/L)** | **Median**  **(ng/L)** | **Frequency**  **(%)** | **Reference** |
| --- | --- | --- | --- | --- | --- | --- | --- | --- | --- | --- |
| Pre-COVID-19  Pre-COVID-19  Pre-COVID-19 | YzRB  YzRB  YzRB  YzRB  YzRB  YzRB  YzRB | Lake Taihu | Dec 2014 | Antibacterials | ERY | ND-15 | - | 1.5 | 78 | [1] |
|  |  |  |  |  | ROX | <LOQ-26 | - | 8.1 | 100 |  |
|  |  |  |  |  | CPFX | 2.9-43 | - | 9.5 | 100 |  |
|  |  |  |  |  | OFX | <LOQ-51 | - | 19 | 100 |  |
|  |  |  |  |  | NOR | 5.2-45 | - | 19 | 100 |  |
|  |  |  |  | Antiviral drugs | - | - | - | - | - |  |
|  |  |  |  | NSAIDs | DFC | ND-26 | - | 4.4 | 88 |  |
|  |  |  |  |  | IBF | 2.0-77 | - | 22 | 100 |  |
|  |  |  |  |  | NPX | ND-22 | - | <LOQ | 50 |  |
|  |  |  |  | Anti-inflammatory corticosteroids | - | - | - | - | - |  |
|  |  | Urban river in Shanghai | 2014-2015 | Antibacterials | AZM | ND-67 | 17 | - | - | [2] |
|  |  |  |  |  | CLR | ND-48 | 12 | - | - |  |
|  |  |  |  | Antiviral drugs | - | - | - | - | - |  |
|  |  |  |  | NSAIDs | IBF | ND-195 | 61 | - | - |  |
|  |  |  |  |  | ATP | 2-7024 | 1132 | - | - |  |
|  |  |  |  |  | DFC | ND-64 | 20 | - | - |  |
|  |  |  |  | Anti-inflammatory corticosteroids | - | - | - | - | - |  |
|  |  | Three Gorges Reservoir | Aug 2015 | Antibacterials | OFX | 10.81-105.23 | 31.12 | - | 100 | [3] |
|  |  |  |  |  | NOR | ND | ND | - | 0 |  |
|  |  |  |  |  | ENR | 9.04-27.36 | 19.32 | - | 100 |  |
|  |  |  |  | Antiviral drugs | - | - | - | - | - |  |
|  |  |  |  | NSAIDs | - | - | - | - | - |  |
|  |  |  |  | Anti-inflammatory corticosteroids | - | - | - | - | - |  |
|  |  | Lake Taihu | Jun and Dec 2015 | Antibacterials | ERY | 12.1-72.6 | 38.6 | 35.5 | 100 | [4] |
|  |  |  |  |  | ROX | <LOQ-18.4 | 14.4 | 15.5 | 17 |  |
|  |  |  |  |  | CPFX | 1.9-16.6 | 5.2 | 8.4 | 86 |  |
|  |  |  |  |  | OFX | <LOQ-5.7 | 2.9 | 2.4 | 71 |  |
|  |  |  |  |  | NOR | 9.2-13.2 | 11 | 11.1 | 100 |  |
|  |  |  |  |  | ENR | <LOQ-39.2 | 16 | 9.7 | 71 |  |
|  |  |  |  | Antiviral drugs | - | - | - | - | - |  |
|  |  |  |  | NSAIDs | - | - | - | - | - |  |
|  |  |  |  | Anti-inflammatory corticosteroids | - | - | - | - | - |  |
|  |  | Dongting Lake | Apr 2015 | Antibacterials | - | - | - | - | - | [5] |
|  |  |  |  | Antiviral drugs | - | - | - | - | - |  |
|  |  |  |  | NSAIDs | IBF | ND-19.8 | - | - | - |  |
|  |  |  |  |  | KPF | ND | - | - | - |  |
|  |  |  |  |  | NPX | ND-3.9 | - | - | - |  |
|  |  |  |  |  | Pre-COVID-19DFC | 2.1-230.4 | - | - | - |  |
|  |  |  |  |  | IM | ND | - | - | - |  |
|  |  |  |  | Anti-inflammatory corticosteroids | - | - | - | - | - |  |
|  |  | Honghu Lake | Dec 2015 and Aug 2016 | Antibacterials | ERY | <LOQ-131.2 | 24.7 | - | 78 | [6] |
|  |  |  |  |  | ROX | <LOQ-2.3 | 1.1 | - | 56 |  |
|  |  |  |  |  | CLR | <LOQ-1.6 | 1.1 | - | 22 |  |
|  |  |  |  |  | AZM | <LOQ-1.5 | 0.6 | - | 44 |  |
|  |  |  |  |  | CPFX | <LOQ-48.7 | 33.7 | - | 67 |  |
|  |  |  |  |  | NOR | <LOQ-53.5 | 51.9 | - | 22 |  |
|  |  |  |  |  | ENR | <LOQ-52.1 | 43.6 | - | 33 |  |
|  |  |  |  |  | ERY | <LOQ-17.4 | 4.2 | - | 75 |  |
|  |  |  |  |  | ROX | <LOQ-0.3 | 0.3 | - | 5 |  |
|  |  |  |  |  | CLR | <LOQ-<LOQ | 0 | - | 0 |  |
|  |  |  |  |  | AZM | <LOQ-0.2 | 0.2 | - | 5 |  |
|  |  |  |  |  | CPFX | <LOQ-28.2 | 13.4 | - | 30 |  |
|  |  |  |  |  | NOR | <LOQ-31 | 22.4 | - | 20 |  |
|  |  |  |  |  | ENR | <LOQ-9.9 | 8.1 | - | 15 |  |
|  |  |  |  | Antiviral drugs | - | - | - | - | - |  |
|  |  |  |  | NSAIDs | - | - | - | - | - |  |
|  |  |  |  | Anti-inflammatory corticosteroids | - | - | - | - | - |  |
|  |  | Poyang Lake | Aug and Dec 2016 | Antibacterials | ERY | ND-<LOQ | - | - | 61.5 | [7] |
|  |  |  |  |  | ROX | ND-29.3 | - | - | 100 |  |
|  |  |  |  |  | ENR | ND-1.08 | - | - | 92.3 |  |
|  |  |  |  | Antiviral drugs | - | - | - | - | - |  |
|  |  |  |  | NSAIDs | - | - | - | - | - |  |
|  |  |  |  | Anti-inflammatory corticosteroids | - | - | - | - | - |  |
|  |  | Nanjing | Nov 2016 | Antibacterials | ERY | 3.3-58 | - | - | - | [8] |
|  |  |  |  |  | ROX | ND | - | - | - |  |
|  |  |  |  |  | CLR | ND | - | - | - |  |
|  |  |  |  |  | CPFX | ND | - | - | - |  |
|  |  |  |  |  | OFX | ND | - | - | - |  |
|  |  |  |  |  | NOR | ND | - | - | - |  |
|  |  |  |  |  | LIN | 11.3-29.4 | - | - | - |  |
|  |  |  |  | Antiviral drugs | - | - | - | - | - |  |
|  |  |  |  | NSAIDs | - | - | - | - | - |  |
|  |  |  |  | Anti-inflammatory corticosteroids | - | - | - | - | - |  |
|  |  | Lu River | 2016-2017 | Antibacterials | AZM | ND-0.57 | 0.38 | - | - | [9] |
|  |  |  |  |  | AMP | 21-68.7 | 31.5 | - | - |  |
|  |  |  |  | Antiviral drugs | - | - | - | - | - |  |
|  |  |  |  | NSAIDs | DFC | ND-552 | 204 | - | - |  |
|  |  |  |  |  | ATP | ND-81 | 6.24 | - | - |  |
|  |  |  |  | Anti-inflammatory corticosteroids | DXM | 48.3-686 | 219 | - | - |  |
|  |  |  |  |  | PN | ND-298 | 67.1 | - | - |  |
|  |  | Zhangxi River |  | Antibacterials | AZM | ND-2.32 | 0.41 | - | - |  |
|  |  |  |  |  | AMP | 20.6-55.6 | 27.4 | - | - |  |
| Pre-COVID-19 |  |  |  | Antiviral drugs | - | - | - | - | - |  |
|  |  |  |  | NSAIDs | DFC | ND-715 | 127 | - | - |  |
|  |  |  |  |  | ATP | ND-178 | 5.47 | - | - |  |
|  |  |  |  | Anti-inflammatory corticosteroids | DXM | 25.1-313 | 127 | - | - |  |
|  |  |  |  |  | PN | ND-276 | 45.2 | - | - |  |
|  |  | Huangpu River and Yangtze River in Shanghai | 2016-2017 | Antibacterials | ERY | 0.1-8.5 | 1.3 | - | 100 | [10] |
|  |  |  |  |  | ROX | 0.1-47.5 | 14.1 | - | 100 |  |
|  |  |  |  |  | OFX | 0.1-1.1 | 0.2 | - | 33 |  |
|  |  |  |  | Antiviral drugs | - | - | - | - | - |  |
|  |  |  |  | NSAIDs | - | - | - | - | - |  |
|  |  |  |  | Anti-inflammatory corticosteroids | - | - | - | - | - |  |
|  |  | Jinsha River | Dec 2017 | Antibacterials | ERY | ND-1.12 | 0.15 | ND | 41.82 | [11] |
|  |  |  |  |  | ROX | ND-18.45 | 1.04 | 0.68 | 80 |  |
|  |  |  |  |  | CLR | ND-3.42 | 0.55 | 0.57 | 83.64 |  |
|  |  |  |  |  | CPFX | ND-5.5 | 0.2 | ND | 3.64 |  |
|  |  |  |  |  | OFX | ND-13.1 | 0.56 | ND | 29.09 |  |
|  |  |  |  |  | NOR | ND-24.77 | 0.87 | ND | 7.27 |  |
|  |  |  |  |  | ENR | ND-7.85 | 1.52 | 1.33 | 60 |  |
|  |  |  |  |  | LIN | ND-1.2 | 0.37 | 0.42 | 67.27 |  |
|  |  |  |  | Antiviral drugs | - | - | - | - | - |  |
|  |  |  |  | NSAIDs | IBF | ND-19.97 | 0.51 | ND | 34.55 |  |
|  |  |  |  |  | DFC | ND-4.2 | 0.08 | ND | 3.64 |  |
|  |  |  |  |  | KPF | ND-0.62 | 0.02 | ND | 3.64 |  |
|  |  |  |  |  | ATP | ND-273.47 | 10.66 | 4.5 | 74.55 |  |
|  |  |  |  | Anti-inflammatory corticosteroids | - | - | - | - | - |  |
|  |  | Dongting Lake | Jul 2017 | Antibacterials | ERY | ND-0.78 | 0.09 | - | - | [12] |
|  |  |  |  |  | ROX | ND-4.87 | 1.06 | - | - |  |
|  |  |  |  |  | CLR | ND-1.72 | 0.42 | - | - |  |
|  |  |  |  |  | LIN | ND-0.31 | 0.09 | - | - |  |
|  |  |  |  |  | CLI | ND-1.29 | 0.24 | - | - |  |
|  |  |  |  | Antiviral drugs | - | - | - | - | - |  |
|  |  |  |  | NSAIDs | DFC | ND-11.1 | 6.71 | - | - |  |
|  |  |  |  |  | KPF | ND-11.3 | 4.67 | - | - |  |
|  |  |  |  |  | ATP | ND-3.88 | 0.56 | - | - |  |
|  |  |  |  | Anti-inflammatory corticosteroids | - | - | - | - | - |  |
|  |  | Xiangjiang River | 2017 | Antibacterials | ERY | <LOQ-43 | 5.6 | 3.1 | 100 | [13] |
|  |  |  |  |  | ROX | 1.4-190 | 12 | 6.1 | 100 |  |
|  |  |  |  |  | CLR | 0.55-100 | 7.9 | 2.8 | 100 |  |
|  |  |  |  |  | AZM | 2.2-99 | 8.8 | 4.7 | 100 |  |
|  |  |  |  |  | CPFX | ND | ND | ND | ND |  |
|  |  |  |  |  | OFX | ND-23 | <LOQ | <LOQ | 81 |  |
| Pre-COVID-19 |  |  |  |  | NOR | ND | ND | ND | ND |  |
|  |  |  |  |  | ENR | ND | ND | ND | ND |  |
|  |  |  |  |  | AMX | 4.6-710 | 52 | 23 | 100 |  |
|  |  |  |  |  | CTX | 3.8-830 | 69 | 33 | 100 |  |
|  |  |  |  | Antiviral drugs | - | - | - | - | - |  |
|  |  |  |  | NSAIDs | IBF | 2.4-320 | 69 | 26 | 100 |  |
|  |  |  |  |  | DFC | ND-32 | 2.9 | 1.9 | 76 |  |
|  |  |  |  |  | IM | ND-2.7 | <LOQ | <LOQ | 47 |  |
|  |  |  |  | Anti-inflammatory corticosteroids | - | - | - | - | - |  |
|  |  | Nanjing section of Yangtze River | Dec 2017 | Antibacterials | ERY | ND | ND | - | ND | [14] |
|  |  |  |  |  | ROX | ND-0.44 | 0.03 | - | 6.25 |  |
|  |  |  |  |  | CLR | ND | ND | - | ND |  |
|  |  |  |  |  | AZM | ND-19.28 | 2.12 | - | 56.25 |  |
|  |  |  |  |  | CPFX | ND | ND | - | ND |  |
|  |  |  |  |  | OFX | ND-35.2 | 3.14 | - | 31.25 |  |
|  |  |  |  |  | NOR | ND-15.4 | 0.96 | - | 6.25 |  |
|  |  |  |  |  | ENR | ND-7.04 | 0.44 | - | 6.25 |  |
|  |  |  |  |  | LIN | ND | ND | - | ND |  |
|  |  |  |  |  | CLI | ND-3.71 | 2.22 | - | 81.25 |  |
|  |  |  |  |  | AMP | ND | ND | - | ND |  |
|  |  |  | Nov 2018 |  | ERY | ND | ND | - | ND |  |
|  |  |  |  |  | ROX | ND | ND | - | ND |  |
|  |  |  |  |  | CLR | ND | ND | - | ND |  |
|  |  |  |  |  | AZM | ND-1.37 | 0.37 | - | 64.29 |  |
|  |  |  |  |  | CPFX | ND | ND | - | ND |  |
|  |  |  |  |  | OFX | ND-12.26 | 2.16 | - | 57.14 |  |
|  |  |  |  |  | NOR | ND | ND | - | ND |  |
|  |  |  |  |  | ENR | ND-2.3 | 0.38 | - | 21.43 |  |
|  |  |  |  |  | LIN | ND-7.74 | 4.39 | - | 92.86 |  |
|  |  |  |  |  | CLI | ND-2.59 | 1.39 | - | 92.86 |  |
|  |  |  |  |  | AMP | ND | ND | - | ND |  |
|  |  |  | Mar 2019 |  | ERY | ND | ND | - | ND |  |
|  |  |  |  |  | ROX | 0.53-1.74 | 1.05 | - | 100 |  |
|  |  |  |  |  | CLR | ND | ND | - | ND |  |
|  |  |  |  |  | AZM | ND-13.5 | 9.2 | - | 87.5 |  |
|  |  |  |  |  | CPFX | ND | ND | - | ND |  |
|  |  |  |  |  | OFX | ND-11.57 | 4.8 | - | 93.75 |  |
|  |  |  |  |  | NOR | ND-6.56 | 1.44 | - | 37.5 |  |
|  |  |  |  |  | ENR | ND | ND | - | ND |  |
|  |  |  |  |  | LIN | ND | ND | - | ND |  |
|  |  |  |  |  | CLI | ND-5.53 | 3.78 | - | 87.5 |  |
| Pre-COVID-19 |  |  |  |  | AMP | ND | ND | - | ND |  |
|  |  |  |  | Antiviral drugs | - | - | - | - | - |  |
|  |  |  |  | NSAIDs | - | - | - | - | - |  |
|  |  |  |  | Anti-inflammatory corticosteroids | - | - | - | - | - |  |
|  |  | Dianshan Lake | 2017-2018 | Antibacterials | - | - | - | - | - | [15] |
|  |  |  |  | Antiviral drugs | - | - | - | - | - |  |
|  |  |  |  | NSAIDs | DFC | ND-22.67 | 4.6 | 0.39 | 50 |  |
|  |  |  |  |  | NPX | ND-1.59 | 0.34 | 0 | 42.5 |  |
|  |  |  |  |  | KPF | ND-19.45 | 2.97 | 0 | 25 |  |
|  |  |  |  |  | IBF | ND-13.02 | 2.04 | 0 | 25 |  |
|  |  |  |  | Anti-inflammatory corticosteroids | - | - | - | - | - |  |
|  |  | Yangtze River and Jialing River | Mar and Sep 2018 | Antibacterials | ERY | ND-1490 | 263.72 | - | 91.67 | [16] |
|  |  |  |  |  | ROX | 2.63-41.22 | 4.65 | - | 100 |  |
|  |  |  |  |  | CPFX | 12.98-14.82 | 13.88 | - | 100 |  |
|  |  |  |  |  | OFX | 6.7-117.71 | 100 | - | 100 |  |
|  |  |  |  |  | NOR | 16.27-26.52 | 19.32 | - | 100 |  |
|  |  |  |  |  | ENR | 9.85-10.54 | 10.14 | - | 100 |  |
|  |  |  |  | Antiviral drugs | - | - | - | - | - |  |
|  |  |  |  | NSAIDs | - | - | - | - | - |  |
|  |  |  |  | Anti-inflammatory corticosteroids | - | - | - | - | - |  |
|  |  | Jiangsu section of the lower Yangtze River | Sep 2018 | Antibacterials | ERY | ND-30 | 4.67 | - | 58 | [17] |
|  |  |  |  |  | ROX | ND-1.75 | 0.44 | - | 50 |  |
|  |  |  |  |  | CLR | ND-2.19 | 0.26 | - | 25 |  |
|  |  |  |  |  | CPFX | ND-0.94 | 0.34 | - | 67 |  |
|  |  |  |  |  | OFX | ND-0.82 | 0.32 | - | 75 |  |
|  |  |  |  |  | NOR | ND-0.82 | 0.32 | - | 67 |  |
|  |  |  |  |  | ENR | ND-0.89 | 0.40 | - | 83 |  |
|  |  |  |  | Antiviral drugs | - | - | - | - | - |  |
|  |  |  |  | NSAIDs | - | - | - | - | - |  |
|  |  |  |  | Anti-inflammatory corticosteroids | - | - | - | - | - |  |
|  |  | Taige Canal Basin | 2018-2019 | Antibacterials | ERY | 6.57-34.3 | 17.8 | - | - | [18] |
|  |  |  |  |  | ROX | 0.505-44.6 | 7.22 | - | - |  |
|  |  |  |  |  | CLR | 0.146-8.42 | 1.74 | - | - |  |
|  |  |  |  |  | CPFX | 7.1-119 | 38.2 | - | - |  |
|  |  |  |  |  | OFX | 1.04-14.3 | 4.71 | - | - |  |
|  |  |  |  |  | NOR | 1.47-85.4 | 13 | - | - |  |
|  |  |  |  |  | ENR | 0.258-23.6 | 3.57 | - | - |  |
|  |  |  |  |  | LIN | 1.26-58.8 | 8.47 | - | - |  |
|  |  |  |  |  | CLI | 222.3-63.7 | 37.9 | - | - |  |
|  |  |  |  | Antiviral drugs | - | - | - | - | - |  |
|  |  |  |  | NASIDs | IBF | 49.5-259 | 107 | - | - |  |
| Pre-COVID-19 |  |  |  |  | ATP | 12.4-88.4 | 42.7 | - | - |  |
|  |  |  |  |  | DFC | 12.7-69.9 | 38.4 | - | - |  |
|  |  |  |  |  | KPF | 7.43-66.2 | 36 | - | - |  |
|  |  |  |  |  | IM | 1.09-20.4 | 5.87 | - | - |  |
|  |  |  |  | Anti-inflammatory corticosteroids | - | - | - | - | - |  |
|  |  | Wujin city of Taihu Lake basin | Nov 2019 | Antibacterials | ERY | ND-5.68 | 0.45 | 0.07 | 74.26 | [19] |
|  |  |  |  |  | ROX | ND-29.8 | 2.36 | 0.9 | 99.16 |  |
|  |  |  |  |  | CLR | ND-12.9 | 2.61 | 0.3 | 84.39 |  |
|  |  |  |  |  | LIN | ND-76.48 | 8.04 | 2.75 | 74.68 |  |
|  |  |  |  |  | CLI | ND-12.9 | 2.59 | 1.18 | 75.11 |  |
|  |  |  |  | Antiviral drugs | - | - | - | - | - |  |
|  |  |  |  | NSAIDs | DFC | ND-506.92 | 22.65 | 5.73 | 81.43 |  |
|  |  |  |  |  | ATP | ND-89.49 | 10.14 | 5 | 86.08 |  |
|  |  |  |  | Anti-inflammatory corticosteroids | - | - | - | - | - |  |
|  |  | Lake Taihu | 2019 | Antibacterials | ROX | ND-13.3 | 1.9 | 0.4 | 79.5 | [20] |
|  |  |  |  |  | CLR | ND-28.3 | 0.2 | 0.2 | 29.5 |  |
|  |  |  |  | Antibacterials | ROX | 35.1-55.9 | 45.5 | 45.5 | - |  |
|  |  |  |  |  | CLR | 9.3-16.3 | 12.8 | 12.8 | - |  |
|  |  |  |  | Antiviral drugs | - | - | - | - | - |  |
|  |  |  |  | NSAIDs | - | - | - | - | - |  |
|  |  |  |  | Anti-inflammatory corticosteroids | - | - | - | - | - |  |
|  |  | Shanghai | May 2019 | Antibacterials | ERY | 0.1-5.32 | 1.43 | 1.03 | 0.78 | [21] |
|  |  |  |  |  | ROX | 0.29-24.55 | 2.75 | 1.11 | 0.85 |  |
|  |  |  |  |  | CLR | 0.06-34.90 | 2.08 | 0.69 | 0.85 |  |
|  |  |  |  |  | AZM | 0.13-12.50 | 1.87 | 0.68 | 0.61 |  |
|  |  |  |  |  | CPFX | 0.88-20.76 | 12.900 | 15.92 | 0.15 |  |
|  |  |  |  |  | OFX | 0.5-177.00 | 13.15 | 6.43 | 1.00 |  |
|  |  |  |  |  | NOR | 14.81-78.68 | 32.53 | 30.09 | 0.20 |  |
|  |  |  |  |  | ENR | 1.00-10.94 | 6.24 | 7.01 | 0.11 |  |
|  |  |  |  |  | MOX | 1.64-30.21 | 10.45 | 8.76 | 0.87 |  |
|  |  |  |  |  | AMX | 5.73-14.8 | 9.77 | 9.08 | 0.35 |  |
|  |  |  |  |  | LIN | 0.3-21.13 | 4.77 | 4.19 | 1/00 |  |
|  |  |  |  | Antiviral drugs | - | - | - | - | - |  |
|  |  |  |  | NSAIDs | - | - | - | - | - |  |
|  |  |  |  | Anti-inflammatory corticosteroids | - | - | - | - | - |  |
|  |  | Hanjiang River | 2019 | Antibacterials | ERY | 0.2-4.4 | - | - | 100 | [22] |
|  |  |  |  |  | ROX | ND-3.1 | - | - | 81.3 |  |
|  |  |  |  |  | CLR | ND-0.9 | - | - | 87.5 |  |
|  |  |  |  |  | AZM | ND-0.6 | - | - | 62.5 |  |
|  |  |  |  |  | CPFX | ND-0 | - | - | 0 |  |
|  |  |  |  |  | OFX | ND-12.2 | - | - | 31.3 |  |
| Pre-COVID-19 |  |  |  |  | NOR | ND-0 | - | - | 0 |  |
|  |  |  |  |  | ENR | ND-2 | - | - | 31/3 |  |
|  |  |  |  | Antibacterials | ERY | ND-2.2 | - | - | 80.4 |  |
|  |  |  |  |  | ROX | ND-3.2 | - | - | 66.1 |  |
|  |  |  |  |  | CLR | ND-0.6 | - | - | 69.6 |  |
|  |  |  |  |  | AZM | ND-0.4 | - | - | 42.9 |  |
|  |  |  |  |  | CPFX | ND-3.6 | - | - | 3.6 |  |
|  |  |  |  |  | OFX | ND-7.8 | - | - | 10.7 |  |
|  |  |  |  |  | NOR | ND-4.7 | - | - | 1.8 |  |
|  |  |  |  |  | ENR | NDD-7.8 | - | - | 19/6 |  |
|  |  |  |  | Antiviral drugs | - | - | - | - | - |  |
|  |  |  |  | NSAIDs | - | - | - | - | - |  |
|  |  |  |  | Anti-inflammatory corticosteroids | - | - | - | - | - |  |
|  |  | Nanjing section of Yangtze River | Nov and Dec 2019 | Antibacterials | ROX | ND-22.36 | 2.76 | - | 78.57 | [23] |
|  |  |  |  |  | CLR | ND-2.93 | 0.46 | - | 28.57 |  |
|  |  |  |  |  | AZM | 2.69-9.12 | 4.43 | - | 57.14 |  |
|  |  |  |  |  | OFX | ND-9.12 | 1.54 | - | 28.57 |  |
|  |  |  |  |  | NOR | 146.72-290.2 | 180.93 | - | 100 |  |
|  |  |  |  |  | ENR | 11.53-26.24 | 20.28 | - | 100 |  |
|  |  |  |  |  | LIN | 11.66-80.18 | 32.02 | - | 100 |  |
|  |  |  |  |  | CLI | 5.84-58.44 | 17.95 | - | 100 |  |
|  |  |  |  | Antiviral drugs | - | - | - | - | - |  |
|  |  |  |  | NSAIDs | - | - | - | - | - |  |
|  |  |  |  | Anti-inflammatory corticosteroids | - | - | - | - | - |  |
|  | PRB | Urban rivers, Guangzhou | Mar and Jul 2015 | Antibacterials | - | - | - | - | - | [24] |
|  |  |  |  | Antiviral drugs | - | - | - | - | - |  |
|  |  |  |  | NSAIDs | NPX | ND-4.28 | 1.11 | 0.63 | 83 |  |
|  |  |  |  |  | IBF | ND-542 | 127 | 58.2 | 92 |  |
|  |  |  |  |  | DFC | ND-645 | 105 | 25.2 | 92 |  |
|  |  |  |  |  | IM | ND-69.6 | 11.6 | 2.61 | 83 |  |
|  |  |  |  | Anti-inflammatory corticosteroids | - | - | - | - | - |  |
|  |  | Qinzhou Bay | Aug and Dec 2017 | Antibacterials | - | - | - | - | - | [25] |
|  |  |  |  | Antiviral drugs | - | - | - | - | - |  |
|  |  |  |  | NSAIDs | DFC | ND-7.71 | 0.824 | ND | 37.5 |  |
|  |  |  |  |  | NPX | ND-0.954 | 0.119 | ND | 12.5 |  |
|  |  |  |  |  | KPF | ND-0.694 | 0.022 | ND | 3.13 |  |
|  |  |  |  |  | IM | ND-4.71 | 0.228 | ND | 6.25 |  |
|  |  |  |  | Anti-inflammatory corticosteroids | - | - | - | - | - |  |
|  |  | Pearl River Delta | Jan and Jul 2018 | Antibacterials | - | - | - | - | - | [26] |
|  |  |  |  | Antiviral drugs | - | - | - | - | - |  |
|  |  |  |  | NSAIDs | - | - | - | - | - |  |
| Pre-COVID-19 |  |  |  | Anti-inflammatory corticosteroids | DXM | <0.3-3.5 | 0.61 | 0.44 | - |  |
|  |  |  |  |  | MP | 0.44-3.8 | 1.0 | 0.6 | - |  |
|  |  |  |  |  | PNL | <0.39-1. | 0.65 | 0.57 | - |  |
|  |  |  |  |  | PN | <0.2-2.3 | 0.44 | 0.24 | - |  |
|  |  |  |  |  | BUD | 0.4-10 | 2.7 | 1.9 | - |  |
|  |  | Pearl River in Guangzhou | Aug and Dec 2018 | Antibacterials | ERY | ND-577 | 137 | 93.1 | 87 | [27] |
|  |  |  |  |  | ROX | 4.03-26.7 | 13.1 | 12.5 | 100 |  |
|  |  |  |  |  | CLR | ND-19.4 | 1.74 | ND | 21 |  |
|  |  |  |  |  | CPFX | 6.31-49.9 | 14.4 | 12.2 | 100 |  |
|  |  |  |  |  | OFX | 2.41-60.3 | 13.3 | 9.89 | 100 |  |
|  |  |  |  |  | NOR | 9.23-27.2 | 13.7 | 12.1 | 100 |  |
|  |  |  |  |  | LIN | 0.35-21.3 | 6.52 | 4.49 | 100 |  |
|  |  |  |  | Antiviral drugs | - | - | - | - | - |  |
|  |  |  |  | NSAIDs | - | - | - | - | - |  |
|  |  |  |  | Anti-inflammatory corticosteroids | - | - | - | - | - |  |
|  |  | Fuxian Lake | Jul 2019 | Antibacterials | ERY | ND-15.05 | 1.98 | - | 52 | [28] |
|  |  |  |  |  | ROX | ND-1.53 | 0.27 | - | 96 |  |
|  |  |  |  |  | CPFX | ND | ND | - | 0 |  |
|  |  |  |  |  | OFX | 0.77-7.3 | 3.4 | - | 100 |  |
|  |  |  |  |  | NOR | ND | ND | - | 0 |  |
|  |  |  |  |  | ENR | ND | ND | - | 0 |  |
|  |  |  |  | Antiviral drugs | - | - | - | - | - |  |
|  |  |  |  | NSAIDs | - | - | - | - | - |  |
|  |  |  |  | Anti-inflammatory corticosteroids | - | - | - | - | - |  |
|  | HaRB  HaRB  HaRB  HaRB | Urban area of Beijing | Jul 2013 to Jun 2014 | Antibacterials | ERY | ND-372 | 20.8 | 4.6 | 98 | [29] |
|  |  |  |  |  | ROX | ND-352 | 26.7 | 3.83 | 98 |  |
|  |  |  |  |  | CPFX | ND-414 | 9.87 | 2.23 | 98 |  |
|  |  |  |  |  | OFX | 0.34-990 | 93.5 | 11.1 | 100 |  |
|  |  |  |  |  | NOR | 0.87-403 | 27.6 | 7.65 | 100 |  |
|  |  |  |  |  | ENR | ND-28.8 | 0.31 | ND | 8 |  |
|  |  |  |  | Antiviral drugs | - | - | - | - | - |  |
|  |  |  |  | NSAIDs | - | - | - | - | - |  |
|  |  |  |  | Anti-inflammatory corticosteroids | - | - | - | - | - |  |
|  |  | Beiyun River, Beijing | Jul and Nov 2015 | Antibacterials | ERY | ND-1320 | - | - | - | [30] |
|  |  |  |  |  | CLR | ND-96.9 | - | - | - |  |
|  |  |  |  | Antiviral drugs | - | - | - | - | - |  |
|  |  |  |  | NSAIDs | DFC | 1.8-121.6 | - | - | - |  |
|  |  |  |  |  | IM | ND-74.9 | - | - | - |  |
|  |  |  |  |  | KPF | ND-65 | - | - | - |  |
|  |  |  |  |  | ATP | ND-3577 | - | - | - |  |
|  |  |  |  | Anti-inflammatory corticosteroids | - | - | - | - | - |  |
| Pre-COVID-19 |  | Haihe River sub-catchment | Oct 2016 and Aug 2017 | Antibacterials | ERY | 38.4-227 | 92.8 | - | 100 | [31] |
|  |  |  |  |  | ROX | 43.9-235 | 84.1 | - | 100 |  |
|  |  |  |  |  | AZM | <LOQ-37.3 | 9 | - | 76 |  |
|  |  |  |  |  | CPFX | 20.9-84.9 | 37.4 | - | 200 |  |
|  |  |  |  |  | OFX | 36.6-374 | 101 | - | 100 |  |
|  |  |  |  |  | NOR | 27.5-188 | 54.5 | - | 100 |  |
|  |  |  |  |  | ENR | 51.6-184 | 78.4 | - | 100 |  |
|  |  |  |  | Antiviral drugs | - | - | - | - | - |  |
|  |  |  |  | NSAIDs | - | - | - | - | - |  |
|  |  |  |  | Anti-inflammatory corticosteroids | - | - | - | - | - |  |
|  |  | Beiyun River, Beijing | Nov and Dec 2017 | Antibacterials | ERY | ND-364 | - | 64 | - | [32] |
|  |  |  |  |  | ROX | ND-327 | - | 37 | - |  |
|  |  |  |  |  | CLR | ND-347 | - | 33.2 | - |  |
|  |  |  |  |  | LIN | 0.13-152 | - | 11.3 | - |  |
|  |  |  |  |  | CLI | 0.16-65.8 | - | 15.6 | - |  |
|  |  |  |  | Antiviral drugs | - | - | - | - | - |  |
|  |  |  |  | NSAIDs | DFC | 0.69-128 | - | 16.7 | - |  |
|  |  |  |  |  | KPF | 3.52-219 | - | 51.9 | - |  |
|  |  |  |  |  | ATP | 6.2-2110 | - | 156 | - |  |
|  |  |  |  |  | IM | 0.48-68.7 | - | 2.54 | - |  |
|  |  |  |  | Anti-inflammatory corticosteroids | - | - | - | - | - |  |
|  |  | Chaobai River, Beijing | Dec 2017 | Antibacterials | ERY | ND-782 | 95.3 | - | 95.5 | [33] |
|  |  |  |  |  | ROX | ND-24.2 | 4.71 | - | 81.8 |  |
|  |  |  |  |  | CPFX | ND-11.4 | 0.89 | - | 4.6 |  |
|  |  |  |  |  | OFX | ND-41.8 | 9.21 | - | 63.6 |  |
|  |  |  |  |  | NOR | ND-105 | 23.1 | - | 31.8 |  |
|  |  |  |  |  | ENR | ND-60.4 | 11.9 | - | 50 |  |
|  |  |  |  | Antiviral drugs | - | - | - | - | - |  |
|  |  |  |  | NSAIDs | - | - | - | - | - |  |
|  |  |  |  | Anti-inflammatory corticosteroids | - | - | - | - | - |  |
|  |  | Four rivers in Beijing | Dec 2017 | Antibacterials | ERY | 58.6-299 | - | - | 97 | [34] |
|  |  |  |  |  | AMP | <0.43-18 | - | - | 37 |  |
|  |  |  |  |  | CTX | 4.74-12.4 | - | - | 83 |  |
|  |  |  |  | Antiviral drugs | - | - | - | - | - |  |
|  |  |  |  | NSAIDs | - | - | - | - | - |  |
|  |  |  |  | Anti-inflammatory corticosteroids | - | - | - | - | - |  |
|  |  | Baiyangdian Lake | Mar 2017 | Antibacterials | ERY | ND-107.26 | 37.89 | - | - | [35] |
|  |  |  |  |  | AZM | ND-215.06 | 45.27 | - | - |  |
|  |  |  |  |  | OFX | ND-64.71 | 27.94 | - | - |  |
|  |  |  |  |  | LIN | ND-407.12 | 107.13 | - | - |  |
|  |  |  |  | Antiviral drugs | - | - | - | - | - |  |
| Pre-COVID-19 |  |  |  | NSAIDs | ATP | ND-71.95 | 31.46 | - | - |  |
|  |  |  |  | Anti-inflammatory corticosteroids | - | - | - | - | - |  |
|  |  | Guanting Reservoir | Aug 2017 | Antibacterials | ERY | ND-27.6 | 11.6 | - | 85.7 | [36] |
|  |  |  |  |  | AZM | ND-16.7 | 9.55 | - | 85.7 |  |
|  |  |  |  |  | OFX | 2.1-26.4 | 13.8 | - | 100 |  |
|  |  |  |  |  | LIN | Nd-40.5 | 23.5 | - | 92.9 |  |
|  |  |  |  | Antiviral drugs | - |  |  |  |  |  |
|  |  |  |  | NSAIDs | ATP | 28.6-507 | 155 | - | 100 |  |
|  |  |  |  | Anti-inflammatory corticosteroids | - | - | - | - | - |  |
|  |  | Wenyu River, Beijing | Jan 2018 | Antibacterials | ERY | ND-84 | - | - | 86 | [37] |
|  |  |  |  |  | ROX | ND-69 | - | - | 86 |  |
|  |  |  |  |  | CPFX | ND-36.9 | - | - | 86 |  |
|  |  |  |  |  | OFX | 80.9-1270 | - | - | 100 |  |
|  |  |  |  |  | NOR | ND-113 | - | - | 71 |  |
|  |  |  |  |  | ENR | ND | - | - | 0 |  |
|  |  |  |  |  | ERY | ND-1.21 | - | - | 57 |  |
|  |  |  |  |  | ROX | ND | - | - | 0 |  |
|  |  |  |  |  | CPFX | ND-4.1 | - | - | 71 |  |
|  |  |  |  |  | OFX | ND-13.2 | - | - | 57 |  |
|  |  |  |  |  | NOR | ND-3.6 | - | - | 57 |  |
|  |  |  |  |  | ENR | ND | - | - | 0 |  |
|  |  |  |  | Antiviral drugs | - | - | - | - | - |  |
|  |  |  |  | NSAIDs | - | - | - | - | - |  |
|  |  |  |  | Anti-inflammatory corticosteroids | - | - | - | - | - |  |
|  |  | Baiyangdian Lake | Apr and Aug 2018 | Antibacterials | CPFX | ND-148 | 124 | - | 13.3 | [38] |
|  |  |  |  |  | OFX | 8.86-428 | 89.2 | - | 100 |  |
|  |  |  |  |  | NOR | ND | ND | - | 0 |  |
|  |  |  |  |  | ENR | ND-111 | 34.3 | - | 33.3 |  |
|  |  |  |  | Antiviral drugs | - | - | - | - | - |  |
|  |  |  |  | NSAIDs | - | - | - | - | - |  |
|  |  |  |  | Anti-inflammatory corticosteroids | - | - | - | - | - |  |
|  |  | Beiyun River, Beijing | Mar, Jul and Oct 2019 | Antibacterials | ERY | 0-642.3 | 24.5 | - | - | [39] |
|  |  |  |  |  | CPFX | 0-232 | 8.8 | - | - |  |
|  |  |  |  |  | OFX | 0-623.6 | 25.1 | - | - |  |
|  |  |  |  |  | NOR | 0-466.3 | 20.2 | - | - |  |
|  |  |  |  | Antiviral drugs | - | - | - | - | - |  |
|  |  |  |  | NSAIDs | KPF | 0-17.3 | 4.5 | - | - |  |
|  |  |  |  |  | DFC | 0-986.5 | 31.9 | - | - |  |
|  |  |  |  |  | IM | 0-52.6 | 3.2 | - | - |  |
|  |  |  |  | Anti-inflammatory corticosteroids | - | - | - | - | - |  |
|  |  | Baiyangdian Lake | May and Nov 2019 | Antibacterials | ERY | 0.18-273 | 29.7 | 16.1 | 100 | [40] |
| Pre-COVID-19 |  |  |  |  | ROX | 0.14-526 | 45.4 | 10.9 | 100 |  |
|  |  |  |  |  | CLR | 0.29-118 | 11.4 | 2.26 | 100 |  |
|  |  |  |  |  | AZM | 0.45-1060 | 46.0 | 1.00 | 100 |  |
|  |  |  |  |  | CPFX | 1.25-29.8 | 6.58 | 4.96 | 100 |  |
|  |  |  |  |  | OFX | 0.33-1000 | 79.8 | 1.48 | 100 |  |
|  |  |  |  |  | NOR | 2.26-123 | 25.4 | 15.7 | 93.9 |  |
|  |  |  |  |  | ENR | <LOQ-182 | 5.29 | 0.79 | 100 |  |
|  |  |  |  | Antiviral drugs | - | - | - | - | - |  |
|  |  |  |  | NSAIDs | - | - | - | - | - |  |
|  |  |  |  | Anti-inflammatory corticosteroids | - | - | - | - | - |  |
|  |  | Xiongan New Area | Jun 2019 | Antibacterials | ERY | ND-0.9 | - | - | 15 | [41] |
|  |  |  |  |  | AZM | ND-2.47 | - | - | 31 |  |
|  |  |  |  |  | CLR | ND-4.95 | - | - | 18 |  |
|  |  |  |  |  | CPFX | ND-15.77 | - | - | 85 |  |
|  |  |  |  |  | OFX | ND-76.25 | - | - | 69 |  |
|  |  |  |  |  | NOR | ND-30.66 | - | - | 67 |  |
|  |  |  |  |  | ENR | ND-2.82 | - | - | 51 |  |
|  |  |  |  |  | AMP | ND-2.35 | - | - | 17 |  |
|  |  |  |  |  | AMX | ND-0.51 | - | - | 46 |  |
|  |  |  |  |  | ERY | ND | - | - | 0 |  |
|  |  |  |  |  | AZM | ND | - | - | 0 |  |
|  |  |  |  |  | CLR | ND | - | - | 0 |  |
|  |  |  |  |  | CPFX | ND-6.38 | - | - | 29 |  |
|  |  |  |  |  | OFX | ND-1.6 | - | - | 11 |  |
|  |  |  |  |  | NOR | ND-0.18 | - | - | 3 |  |
|  |  |  |  |  | ENR | ND-0.24 | - | - | 9 |  |
|  |  |  |  |  | AMP | ND–2.32 |  | - | 62 |  |
|  |  |  |  |  | AMX | ND–103.97 | - | - | 97 |  |
|  |  |  |  | Antiviral drugs | - | - | - | - | - |  |
|  |  |  |  | NSAIDs | - | - | - | - | - |  |
|  |  |  |  | Anti-inflammatory corticosteroids | - | - | - | - | - |  |
|  | YwRB  YwRB | Yellow River in Henan section | 2014 | Antibacterials | ROX | 2.11-126 | 8.77 | 2.56 | 100 | [42] |
|  |  |  |  |  | CPFX | ND-10.1 | 2.96 | 3.46 | 72 |  |
|  |  |  |  |  | OFX | ND-511 | 25 | 3.07 | 80 |  |
|  |  |  |  |  | NOR | ND-1953 | 87 | 4.74 | 96 |  |
|  |  |  |  |  | AMP | ND-15.1 | 1.32 | ND | 32 |  |
|  |  |  |  | Antiviral drugs | - | - | - | - | - |  |
|  |  |  |  | NSAIDs | - | - | - | - | - |  |
|  |  |  |  | Anti-inflammatory corticosteroids | - | - | - | - | - |  |
|  |  | Wei River, Xi 'an | May 2016 | Antibacterials | ERY | 23.3-276.6 | 83.99 | 53.07 | 100 | [43] |
|  |  |  |  |  | ROX | 1.57-59.49 | 25.52 | 23.98 | 100 |  |
| Pre-COVID-19 |  |  |  |  | CLR | ND-10.10 | 9.11 | 9.11 | 25 |  |
|  |  |  |  |  | CPFX | ND-7.32 | 7.32 | 7.32 | 12.5 |  |
|  |  |  |  |  | NOR | ND-39.21 | 18.21 | 8.14 | 37.5 |  |
|  |  |  |  |  | LIN | 3.63-125.33 | 23.81 | 9.1 | 100 |  |
|  |  |  |  | Antiviral drugs | - | - | - | - | - |  |
|  |  |  |  | NSAIDs | - | - | - | - | - |  |
|  |  |  |  | Anti-inflammatory corticosteroids | - | - | - | - | - |  |
|  |  | Huangshui River | 2019 | Antibacterials | ERY | 0.3-16.6 | 4.16 | 2.82 | - | [44] |
|  |  |  |  |  | ROX | 0.12-48.7 | 7.1 | 3.71 | - |  |
|  |  |  |  |  | CLR | 0.06-7.75 | 1.43 | 1.03 | - |  |
|  |  |  |  |  | CPFX | - | - | - | - |  |
|  |  |  |  |  | OFX | ND-8.33 | 0.94 | 0.24 | - |  |
|  |  |  |  |  | NOR | - | - | - | - |  |
|  |  |  |  |  | ENR | - | - | - | - |  |
|  |  |  |  | Antiviral drugs | - | - | - | - | - |  |
|  |  |  |  | NSAIDs | - | - | - | - | - |  |
|  |  |  |  | Anti-inflammatory corticosteroids | - | - | - | - | - |  |
|  |  | Jiaozhou Bay estuary | 2018-2019 | Antibacterials | ERY | ND-38.41 | 13.67 | 9.5 | 67 | [45] |
|  |  |  |  |  | ROX | ND-39.46 | 20.61 | 17.8 | 42 |  |
|  |  |  |  |  | AZM | ND-7.95 | 5.1 | 5.68 | 58 |  |
|  |  |  |  |  | CPFX | ND-42.74 | 26.6 | 27.02 | 67 |  |
|  |  |  |  |  | OFX | 3.87-254.74 | 102.6 | 104.97 | 100 |  |
|  |  |  |  |  | NOR | 7.45-173.44 | 54.68 | 36.2 | 100 |  |
|  |  |  |  |  | ENR | 7.9-18.81 | 13.5 | 13.62 | 100 |  |
|  |  |  |  |  | AMX | 34.6-786.4 | 173.8 | 107.66 | 100 |  |
|  |  |  |  | Antiviral drugs | - | - | - | - | - |  |
|  |  |  |  | NSAIDs | - | - | - | - | - |  |
|  |  |  |  | Anti-inflammatory corticosteroids | - | - | - | - | - |  |
|  |  | Jiaozhou Bay estuary | May to Oct 2019 | Antibacterials | ERY | 1.59-4.02 | - | 2.59 | 100 | [46] |
|  |  |  |  |  | ROX | <LOQ-1.34 | - | <LOQ | 15 |  |
|  |  |  |  |  | AZM | 4.51-8.32 | - | 6.19 | 100 |  |
|  |  |  |  |  | CPFX | 2.19-51.8 | - | 20.7 | 100 |  |
|  |  |  |  |  | OFX | <LOQ-144 | - | 1.92 | 100 |  |
|  |  |  |  |  | NOR | 7.94-71.3 | - | 26.6 | 100 |  |
|  |  |  |  |  | ENR | <LOQ-15.2 | - | <LOQ | 15 |  |
|  |  |  |  |  | AMX | 12.03-1334 | - | 371 | 100 |  |
|  |  |  |  | Antiviral drugs | - | - | - | - | - |  |
|  |  |  |  | NSAIDs | - | - | - | - | - |  |
|  |  |  |  | Anti-inflammatory corticosteroids | - | - | - | - | - |  |
|  |  | Fenhe River | Nov 2019 | Antibacterials | ERY | 7.33-36.8 | 18.54 | - | 100 | [47] |
|  |  |  |  |  | ROX | 6.53-131.49 | 35.42 | - | 100 |  |
| Pre-COVID-19  Pre-COVID-19 |  |  |  |  | CLR | <1.58-8.26 | 0.44 | - | 17 |  |
|  |  |  |  |  | AZM | 4.18-68.86 | 12.18 | - | 100 |  |
|  |  |  |  |  | CPFX | 9.9-28.58 | 14.75 | - | 100 |  |
|  |  |  |  |  | OFX | <1.77-154.79 | 7.68 | - | 22 |  |
|  |  |  |  |  | NOR | 8.66-53.28 | 14.89 | - | 100 |  |
|  |  |  |  |  | ENR | 6.39-117.9 | 15.42 | - | 100 |  |
|  |  |  |  | Antiviral drugs | - | - | - | - | - |  |
|  |  |  |  | NSAIDs | - | - | - | - | - |  |
|  |  |  |  | Anti-inflammatory corticosteroids | - | - | - | - | - |  |
|  | HuRB | Huai River | Dec 2018 | Antibacterials | CPFX | ND-16 | 2 | - | 75 | [48] |
|  |  |  |  |  | OFX | ND-5.7 | 0.94 | - | 56.25 |  |
|  |  |  |  |  | NOR | ND-52 | 14 | - | 90.625 |  |
|  |  |  |  |  | ENR | ND-1.4 | 0.36 | - | 87.5 |  |
|  |  |  |  | Antiviral drugs | - | - | - | - | - |  |
|  |  |  |  | NSAIDs | - | - | - | - | - |  |
|  |  |  |  | Anti-inflammatory corticosteroids | - | - | - | - | - |  |
|  |  | Huai River in Henan section | 2013-2014 | Antibacterials | ROX | ND-33.9 | 3.7 | 2.14 | 75 | [42] |
|  |  |  |  |  | CPFX | ND-6.86 | 0.69 | ND | 6.25 |  |
|  |  |  |  |  | OFX | ND-19.3 | 1.21 | ND | 6.25 |  |
|  |  |  |  |  | NOR | ND-8.29 | 0.73 | ND | 6.25 |  |
|  |  |  |  |  | AMP | 3.93-54.2 | 20.4 | 15.3 | 87.5 |  |
|  |  |  |  | Antiviral drugs | - | - | - | - | - |  |
|  |  |  |  | NSAIDs | - | - | - | - | - |  |
|  |  |  |  | Anti-inflammatory corticosteroids | - | - | - | - | - |  |
|  |  | Luoma Lake | Dec 2019 | Antibacterials | - | - | - | - | - | [49] |
|  |  |  |  | Antiviral drugs | - | - | - | - | - |  |
|  |  |  |  | NSAIDs | IBF | ND-18.95 | 0.56 | - | 5 |  |
|  |  |  |  |  | NPX | ND-33.99 | 5.41 | - | 77 |  |
|  |  |  |  |  | KPF | 3.47-531.43 | 125.85 | - | 100 |  |
|  |  |  |  |  | IM | ND-8.3 | 3.59 | - | 84 |  |
|  |  |  |  | Anti-inflammatory corticosteroids | - | - | - | - | - |  |
|  |  | Luoma Lake, Zhongyun River and Yihe River, Jiangsu | Nov 2019 | Antibacterials | ERY | <LOQ | <LOQ | <LOQ | 0 | [50] |
|  |  |  |  |  | ROX | <LOQ-566.78 | 40.87 | 15.30 | 54,6 |  |
|  |  |  |  |  | CLR | <LOQ-66.7 | 25.65 | 8.18 | 6.8 |  |
|  |  |  |  |  | AZM | <LOQ-5.69 | 2.41 | 2.14 | 11.4 |  |
|  |  |  |  |  | CPFX | <LOQ-12.5 | 11.3 | 11.3 | 4.6 |  |
|  |  |  |  |  | OFX | <LOQ-22.79 | 7.50 | 3.20 | 9.1 |  |
|  |  |  |  |  | NOR | <LOQ | <LOQ | <LOQ | 0 |  |
|  |  |  |  |  | ENR | <LOQ | <LOQ | <LOQ | 0 |  |
|  |  |  |  |  | LIN | <LOQ-81.34 | 10.83 | 4.58 | 79.6 |  |
|  |  |  |  |  | CLI | <LOQ-216.38 | 10.02 | 2.01 | 72.7 |  |
|  |  |  |  | Antiviral drugs | - | - | - | - | - |  |
|  |  |  |  | NSAIDs | - | - | - | - | - |  |
|  |  |  |  | Anti-inflammatory corticosteroids | - | - | - | - | - |  |
|  | LRB  LRB | Liaohe River basin | Jun 2019 | Antibacterials | CPFX | ND-5.02 | 0.17 | - | 3.45 | [51] |
|  |  |  |  |  | OFX | ND-27.21 | 3.68 | - | 27.59 |  |
|  |  |  |  |  | NOR | ND-13.65 | 0.65 | - | 6.9 |  |
|  |  |  |  |  | ENR | ND-5.47 | 0.35 | - | 13.79 |  |
|  |  |  |  |  | AMX | ND-128.59 | 21.32 | - | 72.41 |  |
|  |  |  |  | Antiviral drugs | - | - | - | - | - |  |
|  |  |  |  | NSAIDs | - | - | - | - | - |  |
|  |  |  |  | Anti-inflammatory corticosteroids | - | - | - | - | - |  |
|  | SRB | Main stream of Songhua River | 2016 | Antibacterials | ROX | 0.2-11.5 | - | - | 100 | [52] |
|  |  |  |  |  | AZM | 0.06-5.14 | - | - | 100 |  |
|  |  |  |  |  | CLR | ND-4.17 | - | - | 96.8 |  |
|  |  |  |  |  | OFX | 0.01-1.8 | - | - | 100 |  |
|  |  |  |  |  | NOR | ND-2.4 | - | - | 84.3 |  |
|  |  |  |  |  | CTX | ND-5.25 | - | - | 72 |  |
|  |  |  |  | Antiviral drugs | - | - | - | - | - |  |
|  |  |  |  | NSAIDs | - | - | - | - | - |  |
|  |  |  |  | Anti-inflammatory corticosteroids | - | - | - | - | - |  |
|  |  | Songhua River | 2018-2019 | Antibacterials | ERY | ND-37.3 | 5.1 | 3.6 | 92.6 | [53] |
|  |  |  |  |  | CPFX | ND-35.5 | 6.2 | 2.5 | 77.8 |  |
|  |  |  |  |  | OFX | <LOQ-26.2 | 6 | 2.8 | 100 |  |
|  |  |  |  |  | NOR | ND-93.4 | 27.4 | 25.4 | 96.3 |  |
|  |  |  |  |  | AMX | 15.9-134 | 46.1 | 29.1 | 100 |  |
|  |  |  |  |  | LIN | ND-84.4 | 6.8 | 3.4 | 88.9 |  |
|  |  |  |  | Antiviral drugs | - | - | - | - | - |  |
|  |  |  |  | NSAIDs | IBF | 2.2-53.8 | 17 | 15.1 | 100 |  |
|  |  |  |  |  | ATP | ND-65.8 | 18 | 13.8 | 88.9 |  |
|  |  |  |  |  | NPX | 0.4-15.4 | 1.7 | 0.8 | 100 |  |
|  |  |  |  |  | DFC | 1.2-45 | 9 | 5 | 100 |  |
|  |  |  |  | Anti-inflammatory corticosteroids | - | - | - | - | - |  |
| During COVID-19  During COVID-19  During COVID-19 | YzRB  YzRB | Lakes in Wuhan | Jun and Oct 2020 | Antibacterials | ERY | 0.91-12.9 | - | 1.15 | 78.9 | [54] |
|  |  |  |  |  | CLR | 0.18-266 | - | 5.93 | 89.5 |  |
|  |  |  |  |  | AZM | 3.14-935 | - | 0.49 | 100 |  |
|  |  |  |  |  | CPFX | ND | - | - | - |  |
|  |  |  |  |  | OFX | 39-172 | - | 106 | 10.5 |  |
|  |  |  |  |  | NOR | 0.49-4.51 | - | 2.5 | 5.3 |  |
|  |  |  |  |  | ENR | ND | - | - | - |  |
|  |  |  |  |  | MOX | ND | - | - | - |  |
|  |  |  |  | Antiviral drugs | RBV | 1.04-52.2 | - | 4.36 | 89.5 |  |
|  |  |  |  | NSAIDs | - | - | - | - | - |  |
|  |  |  |  | Anti-inflammatory corticosteroids | PNL | - | - | - | - |  |
|  |  |  |  |  | BUD | 6.65-15.5 | - | 11.1 | 10.5 |  |
|  |  | Wuhan | Apr, Jul and Dec 2020 | Antiviral drugs | RTV | 2.0-10.0 | 4.2 | - | - | [55] |
|  |  |  |  |  | LPV | ND-14.5 | 4.7 | - | - |  |
|  |  |  |  |  | RTV | 4.0-5.5 | 4.8 | - | - |  |
|  |  |  |  |  | LPV | 29.2-52.7 | 41 | - | - |  |
|  |  |  |  | Antibacterials | - | - | - | - | - |  |
|  |  |  |  | NSAIDs | - | - | - | - | - |  |
|  |  |  |  | Anti-inflammatory corticosteroids | - | - | - | - | - |  |
|  |  | Zijiang River | Nov 2020 | Antibacterials | ERY | <LOQ-14.74 | 7.97 | 6.34 | 85 | [56] |
|  |  |  |  |  | ROX | <LOQ-21.92 | 8.58 | 3.06 | 74 |  |
|  |  |  |  |  | AZM | <LOQ-19.75 | 5.70 | 4.36 | 93 |  |
|  |  |  |  |  | CPFX | <LOQ-7.25 | 3.41 | 2.99 | 96 |  |
|  |  |  |  |  | OFX | <LOQ-6.08 | 3.76 | 3.08 | 74 |  |
|  |  |  |  |  | NOR | <LOQ-5.51 | 2.96 | 1.99 | 74 |  |
|  |  |  |  |  | ENR | <LOQ-1.32 | 1.06 | 0.85 | 67 |  |
|  |  |  |  |  | AMX | 26.98-91.35 | 51.87 | 49.61 | 100 |  |
|  |  |  |  | Antiviral drugs | - | - | - | - | - |  |
|  |  |  |  | NSAIDs | - | - | - | - | - |  |
|  |  |  |  | Anti-inflammatory corticosteroids | - | - | - | - | - |  |
|  |  | Chaohu Lake | Jul to Aug2020 | Antibacterials | ERY | ND-200 | 7.01 | ND | 0.12 | [57] |
|  |  |  |  |  | CPFX | ND- 187.23 | 13.5 | ND | 0.23 |  |
|  |  |  |  |  | OFX | ND-55.10 | 4.55 | ND | 0.11 |  |
|  |  |  |  |  | NOR | ND-80.63 | 1.79 | ND | 0.04 |  |
|  |  |  |  | Antiviral drugs | - | - | - | - | - |  |
|  |  |  |  | NSAIDs | - | - | - | - | - |  |
|  |  |  |  | Anti-inflammatory corticosteroids | - | - | - | - | - |  |
|  |  | Nanjing section of Yangtze River | Jan 2020 | Antibacterials | ROX | ND-2.06 | 1.32 | - | 83.33 | [22] |
|  |  |  |  |  | CLR | 9.88-53.9 | 1.54 | - | 100 |  |
|  |  |  |  |  | AZM | ND | ND | - | 0 |  |
|  | YzRB  YzRB  YzRB |  |  |  | OFX | ND-34.66 | 6.82 | - | 75 |  |
|  |  |  |  |  | NOR | ND | ND | - | 0 |  |
|  |  |  |  |  | ENR | 4.25-20.51 | 11.17 | - | 100 |  |
|  |  |  |  |  | LIN | 4.02-9.85 | 7.3 | - | 100 |  |
|  |  |  |  |  | CLI | 9.88-53.9 | 22.23 | - | 100 |  |
|  |  |  |  | Antiviral drugs | - | - | - | - | - |  |
|  |  |  |  | NSAIDs | - | - | - | - | - |  |
|  |  |  |  | Anti-inflammatory corticosteroids | - | - | - | - | - |  |
|  |  | Nanming River | Oct 2020 | Antibacterials | OFX | ND-645.1 | 136.6 | 88.8 | 91.2 | [58] |
|  |  |  |  |  | NOR | ND-285.4 | 36.6 | 17.5 | 61.8 |  |
|  |  |  |  | Antiviral drugs | - | - | - | - | - |  |
|  |  |  |  | NSAIDs | - | - | - | - | - |  |
|  |  |  |  | Anti-inflammatory corticosteroids | - | - | - | - | - |  |
|  |  | Chishui River | Apr and Aug 2021 | Antibacterials | ERY | ND-4.84 | 0.9 | - | 20 | [59] |
|  |  |  |  |  | CPFX | ND-6.62 | 2.17 | - | 11 |  |
| During COVID-19 |  |  |  |  | OFX | 43.21-170.26 | 75.74 | - | 100 |  |
|  |  |  |  |  | NOR | ND-15.22 | 0.61 | - | 4 |  |
|  |  |  |  |  | ENR | ND-8.66 | 4.76 | - | 60 |  |
|  |  |  |  |  | LIN | 3.21-11.72 | 6.17 | - | 100 |  |
|  |  |  |  | Antiviral drugs | - | - | - | - | - |  |
|  |  |  |  | NSAIDs | - | - | - | - | - |  |
|  |  |  |  | Anti-inflammatory corticosteroids | - | - | - | - | - |  |
|  |  | Wuhan section of Yangtze River | 2020-2021 | Antibacterials | ERY | <LOQ-23.3 | 2.01 | - | 14.8 | [60] |
|  |  |  |  |  | ROX | <LOQ-26.78 | 2.04 | - | 85.2 |  |
|  |  |  |  |  | AZM | <LOQ-47.34 | 2.55 | - | 79.6 |  |
|  |  |  |  |  | CLR | <LOQ-49.72 | 2.66 | - | 90.7 |  |
|  |  |  |  |  | CPFX | <LOQ-4.99 | 1.75 | - | 3.7 |  |
|  |  |  |  |  | OFX | <LOQ-5.25 | 0.81 | - | 14.8 |  |
|  |  |  |  |  | NOR | <LOQ-4.23 | 1.12 | - | 3.7 |  |
|  |  |  |  |  | ENR | <LOQ-20.41 | 3.08 | - | 29.6 |  |
|  |  |  |  | Antiviral drugs | - | - | - | - | - |  |
|  |  |  |  | NSAIDs | - | - | - | - | - |  |
|  |  |  |  | Anti-inflammatory corticosteroids | - | - | - | - | - |  |
|  |  | Danjiangkou Reservoir | Jun 2021 | Antibacterials | ERY | 0.10-9.98 | 0.94 | 0.17 | 100 | [61] |
|  |  |  |  |  | ROX | 0-10.74 | 0.60 | 0 | 35.71 |  |
|  |  |  |  |  | CLR | 0-6.78 | 0.35 | 0 | 32.14 |  |
|  |  |  |  |  | OFX | 0-130.31 | 5.64 | 0 | 21.43 |  |
|  |  |  |  |  | ENR | 0-0.25 | 0.03 | 0 | 3.57 |  |
|  |  |  |  | Antiviral drugs | RBV | 0-6.06 | 0.62 | 0 | 28.57 |  |
|  |  |  |  |  | OTV | 0-4.10 | 0.38 | 0.03 | 78.57 |  |
|  |  |  |  | NSAIDs | - | - | - | - | - |  |
|  |  |  |  | Anti-inflammatory corticosteroids | - | - | - | - | - |  |
|  |  | Lake Taihu | 2021 | Antibacterials | ROX | ND-0.4 | 2.6 | 0.7 | 95.6 | [20] |
|  |  |  |  |  | CLR | ND-8.1 | 1 | 0.1 | 70.6 |  |
|  |  |  |  | Antibacterials | ROX | 9.3-31.3 | 20.3 | 20.3 | - |  |
|  |  |  |  |  | CLR | 2.7-5.5 | 4.1 | 4.1 | - |  |
|  |  |  |  | Antiviral drugs | - | - | - | - | - |  |
|  |  |  |  | NSAIDs | - | - | - | - | - |  |
|  |  |  |  | Anti-inflammatory corticosteroids | - | - | - | - | - |  |
|  |  | Wuhan section of Yangtze River | Jun 2021 | Antibacterials | ERY | ND-0.46 | 0.13 | - | 55.56 | [62] |
|  |  |  |  |  | ROX | 0.86-469.30 | 32.61 | - | 100 |  |
|  |  |  |  |  | OFX | 20.06-1395.88 | 259.36 | - | 100 |  |
|  |  |  |  |  | ENR | 0.42-1.44 | 0.53 | - | 100 |  |
|  |  |  |  | Antiviral drugs | - | - | - | - | - |  |
|  |  |  |  | NSAIDs | - | - | - | - | - |  |
|  |  |  |  | Anti-inflammatory corticosteroids | - | - | - | - | - |  |
| During COVID-19 |  | Wuhan section of Yangtze River | 2021-2022 | NSAIDs | DFC | ND | ND | - | 0 | [63] |
|  |  |  |  |  | IBF | ND-164.9 | 30.10 | - | 88.8 |  |
|  |  |  |  |  | NPX | ND | ND | - | 0 |  |
|  |  |  |  |  | ATP | ND-211.8 | 11.83 | - | 88.8 |  |
|  |  |  |  | Antibacterials | - | - | - | - | - |  |
|  |  |  |  | Antiviral drugs | - | - | - | - | - |  |
|  |  |  |  | Anti-inflammatory corticosteroids | - | - | - | - | - |  |
|  |  | Jiangsu section of the lower Yangtze River | Apr and Sep 2021 | Antibacterials | ERY | ND-2.58 | - | - | 15.8 | [64] |
|  |  |  |  |  | ROX | ND-18.06 | - | - | 84.2 |  |
|  |  |  |  |  | CLR | 2.48-35.32 | - | - | 100 |  |
|  |  |  |  |  | AZM | ND-22.76 | - | - | 42.1 |  |
|  |  |  |  |  | CPFX | 17.31-2717.31 | - | - | 100 |  |
|  |  |  |  |  | OFX | 8..04- 493.73 | - | - | 100 |  |
|  |  |  |  |  | NOR | 89.7- 2178.01 | - | - | 100 |  |
|  |  |  |  |  | ENR | 7.06-81.74 | - | - | 100 |  |
|  |  |  |  |  | LIN | 0.3-9.26 | - | - | 100 |  |
|  |  |  |  |  | CLI | 3.01-2406.09 | - | - | 100 |  |
|  |  |  |  | Antiviral drugs | - | - | - | - | - |  |
|  |  |  |  | NSAIDs | - | - | - | - | - |  |
|  |  |  |  | Anti-inflammatory corticosteroids | - | - | - | - | - |  |
|  |  | Zijiang River | Apr 2021 | Antibacterials | ERY | <LOQ-8.59 | 4.45 | 3.26 | 70 | [65] |
|  |  |  |  |  | ROX | <LOQ-10.32 | 5.73 | 2.03 | 56 |  |
|  |  |  |  |  | AZM | <LOQ-3.73 | 2.21 | 1.36 | 78 |  |
|  |  |  |  |  | CPFX | <LOQ | <LOQ | <LOQ | 33 |  |
|  |  |  |  |  | OFX | <LOQ | <LOQ | <LOQ | 22 |  |
|  |  |  |  |  | NOR | <LOQ-5.47 | 2.3 | 0.98 | 56 |  |
|  |  |  |  |  | ENR | <LOQ-4.08 | 2.59 | <LOQ | 26 |  |
|  |  |  |  |  | AMX | 3.18-34.73 | 13.81 | 11.12 | 100 |  |
|  |  |  |  | Antiviral drugs | - | - | - | - | - |  |
|  |  |  |  | NSAIDs | - | - | - | - | - |  |
|  |  |  |  | Anti-inflammatory corticosteroids | - | - | - | - | - |  |
|  |  | Changshou Lake | Jan, May and Sep 2022 | Antibacterials | ERY | 12.6-48.9 | 33.7 | 31.9 | 100 | [56] |
|  |  |  |  |  | ROX | 0.219-0.892 | 0.5 | 0.46 | 100 |  |
|  |  |  |  |  | CPFX | 2.21 - 5.50 | 3.48 | 3.22 | 100 |  |
|  |  |  |  |  | OFX | ND - 34.8 | 4.45 | 0.399 | 91 |  |
|  |  |  |  |  | NOR | 3.81 - 12.6 | 6.44 | 6.1 | 100 |  |
|  |  |  |  |  | ENR | 0.473 - 3.19 | 1.25 | 1.25 | 100 |  |
|  |  |  |  |  | ERY | 19.2 - 142 | 46 | 34.4 | 100 |  |
|  |  |  |  |  | ROX | 0.047 - 2.39 | 0.452 | 0.209 | 100 |  |
|  |  |  |  |  | CPFX | 1.34 - 55.3 | 7.06 | 2.26 | 100 |  |
|  |  |  |  |  | OFX | ND - 20.4 | 2.35 | 0.601 | 73 |  |
| During COVID-19 |  |  |  |  | NOR | 0.234 - 220 | 20.6 | 0.444 | 100 |  |
|  |  |  |  |  | ENR | 0.472 - 5.87 | 1.64 | 1.24 | 100 |  |
|  |  |  |  | Antiviral drugs | - | - | - | - | - |  |
|  |  |  |  | NSAIDs | - | - | - | - | - |  |
|  |  |  |  | Anti-inflammatory corticosteroids | - | - | - | - | - |  |
|  |  | The Panlong River | Apr 2022 | Antibacterials | OFX | ND-5.47 | 2.43 | 3.68 | 75 | [66] |
|  |  |  |  |  | LIN | 0.71-1.37 | 1.10 | 1.13 | 100 |  |
|  |  |  |  | Antiviral drugs | - | - | - | - | - |  |
|  |  |  |  | NSAIDs | - | - | - | - | - |  |
|  |  |  |  | Anti-inflammatory corticosteroids | - | - | - | - | - |  |
|  |  | Lake Dianchi |  | Antibacterials | OFX | ND-4.55 | 1.72 | 1.32 | 50 |  |
|  |  |  |  |  | LIN | ND-1.51 | 0.66 | 0.80 | 62.5 |  |
|  |  |  |  | Antiviral drugs | - | - | - | - | - |  |
|  |  |  |  | NSAIDs | - | - | - | - | - |  |
|  |  |  |  | Anti-inflammatory corticosteroids | - | - | - | - | - |  |
|  |  | The river confluence in Shanghai | Aug 2022 | Antibacterials | ERY | 0.24-3.33 | 0.65 | - | 100 | [67] |
|  |  |  |  |  | ROX | 0.25-1.01 | 0.36 | - | 100 |  |
|  |  |  |  |  | CPFX | <LOQ-5.07 | 0.96 | - | 100 |  |
|  |  |  |  |  | OFX | 0.66-21.22 | 1.25 | - | 100 |  |
|  |  |  |  |  | NOR | <LOQ-3.52 | 1.38 | - | 100 |  |
|  |  |  |  | Antiviral drugs | - | - | - | - | - |  |
|  |  |  |  | NSAIDs | - | - | - | - | - |  |
|  |  |  |  | Anti-inflammatory corticosteroids | - | - | - | - | - |  |
|  | PRB  PRB  PRB | Wanfeng Lake | Mar 2021 | Antibacterials | OFX | ND- 246.96 | 169.48 | 149.58 | 100 | [68] |
|  |  |  |  |  | LIN | ND | ND | ND | ND |  |
|  |  |  |  | Antiviral drugs | - | - | - | - | - |  |
|  |  |  |  | NSAIDs | - | - | - | - | - |  |
|  |  |  |  | Anti-inflammatory corticosteroids | - | - | - | - | - |  |
|  |  | Pearl River Delta | Jul 2022 | Antibacterials | ERY | 5.05-22.53 | 6.95 | - | - | [69] |
|  |  |  |  |  | AZM | ND-877.52 | 3.66 | - | - |  |
|  |  |  |  |  | CPFX | 0.7-33.39 | 2.13 | - | - |  |
|  |  |  |  |  | OFX | ND-95.26 | 0.69 | - | - |  |
|  |  |  |  |  | NOR | 0.7-20.54 | 1.82 | - | - |  |
|  |  |  |  |  | ENR | 0.19-3.77 | 0.53 | - | - |  |
|  |  |  |  |  | MOX | 0.18-5.5 | 1.31 | - | - |  |
|  |  |  |  |  | CLI | ND-183.52 | 1.66 | - | - |  |
|  |  |  |  | Antiviral drugs | LPV | 0.59-21.54 | 1.5 | - | - |  |
|  |  |  |  |  | RTV | 1.04-9.47 | 3.16 | - | - |  |
|  |  |  |  | NSAIDs | - | - | - | - | - |  |
|  |  |  |  | Anti-inflammatory corticosteroids | PN | ND-16.15 | 3.01 | - | - |  |
|  |  |  |  |  | PNL | 0.04-5.16 | 1.753.01 | - | - |  |
|  |  |  |  |  | HYD | ND-9.64 | 2.9 | - | - |  |
|  |  |  |  |  | MP | ND-6.4 | 1.07 | - | - |  |
|  |  |  |  |  | DXM | ND-0.66 | 0.25 | - | - |  |
|  |  | Baini River | May 2022 | Antibacterials | ERY | ND-17.99 | - | - | - | [70] |
|  |  |  |  |  | CPFX | ND-36.55 | - | - | - |  |
|  |  |  |  |  | OFX | ND-20.89 | - | - | - |  |
|  |  |  |  |  | NOR | 22.21-242.07 | - | - | - |  |
|  |  |  |  |  | ENR | ND | - | - | - |  |
|  |  |  |  |  | LIN | 21.41-105.36 | - | - | - |  |
|  |  |  |  | Antiviral drugs | - | - | - | - | - |  |
| During COVID-19 |  |  |  | NSAIDs | - | - | - | - | - |  |
|  |  |  |  | Anti-inflammatory corticosteroids | - | - | - | - | - |  |
|  |  | Dongjiang River | Sep 2022 | Antibacterials | ERY | 1.98-2.52 | 2.21 | 2.19 | - | [71] |
|  |  |  |  |  | ROX | ND-8.46 | 2.71 | 1.52 | - |  |
|  |  |  |  |  | AZM | 0.35-4.25 | 1.77 | 1.84 | - |  |
|  |  |  |  |  | CPFX | ND-21.98 | 5.85 | 2.93 | - |  |
|  |  |  |  |  | OFX | ND-172.15 | 13.64 | 5.95 | - |  |
|  |  |  |  |  | NOR | ND-46.63 | 13.19 | 5.82 | - |  |
|  |  |  |  |  | ENR | 0.63-2.69 | 1.42 | 1.37 | - |  |
|  |  |  |  |  | AMX | ND-112.7 | 12.98 | 6.48 |  |  |
|  |  |  |  | Antiviral drugs | - | - | - | - | - |  |
|  |  |  |  | NSAIDs | - | - | - | - | - |  |
|  |  |  |  | Anti-inflammatory corticosteroids | - | - | - | - | -- |  |
|  |  | Yuqiao Reservoir, Tianjin | Mar to Jul 2020 | Antibacterials | ERY | 0.93-2.01 | 1.36 | - | 100 | [72] |
|  |  |  |  |  | ROX | 0.52-0.94 | 0.66 | - | 100 |  |
|  |  |  |  |  | AZM | 0.27-1.21 | 0.53 | - | 100 |  |
|  |  |  |  |  | CPFX | ND | ND | - | ND |  |
|  |  |  |  |  | OFX | ND | ND | - | ND |  |
|  |  |  |  |  | NOR | ND | ND | - | ND |  |
|  |  |  |  |  | ENR | ND | ND | - | ND |  |
|  |  |  |  | Antiviral drugs | - | - | - | - | - |  |
|  |  |  |  | NSAIDs | - | - | - | - | - |  |
|  |  |  |  | Anti-inflammatory corticosteroids | - | - | - | - | -- |  |
|  | HaRB  HaRB | Urban river, Beijing | Nov 2020 | Antibacterials | ERY | 0.08-51.21 | 6.12 | 0.68 | 100 | [73] |
|  |  |  |  |  | ROX | <LOQ-96.83 | 2.56 | 0.27 | 95 |  |
|  |  |  |  |  | CLR | <LOQ-20.82 | 2.3 | 0.22 | 86.7 |  |
|  |  |  |  |  | CPFX | <LOQ-4.27 | 0.2 | <LOQ | 16.7 |  |
|  |  |  |  |  | OFX | <LOQ-106.77 | 6.44 | <LOQ | 38.3 |  |
|  |  |  |  |  | ENR | 1.33-31.93 | 4.97 | 3.8 | 100 |  |
|  |  |  |  | Antiviral drugs | RBV | <LOQ-64.07 | 11.84 | 8.31 | 88.3 |  |
|  |  |  |  |  | OTV | <LOQ-49.76 | 1.91 | 0.04 | 80 |  |
|  |  |  |  | NSAIDs | - | - | - | - | - |  |
|  |  |  |  | Anti-inflammatory corticosteroids | - | - | - | - | - |  |
|  |  |  | Apr 2021 | Antibacterials | ERY | <LOQ-201.68 | 18.28 | 1.22 | 95 |  |
|  |  |  |  |  | ROX | <LOQ-96.83 | 11.4 | 0.41 | 90 |  |
|  |  |  |  |  | CLR | <LOQ-55.58 | 6.28 | 0.22 | 70 |  |
|  |  |  |  |  | CPFX | <LOQ-8.21 | 0.63 | <LOQ | 35 |  |
|  |  |  |  |  | OFX | <LOQ-99.98 | 8.52 | 0.37 | 56.7 |  |
|  |  |  |  |  | ENR | 0.23-3.43 | 1.86 | 1.98 | 100 |  |
|  |  |  |  | Antiviral drugs | RBV | 0.07-24.38 | 9.66 | 9.87 | 100 |  |
|  |  |  |  |  | OTV | <LOQ-69.92 | 2.44 | <LOQ | 48.3 |  |
| During COVID-19  During COVID-19 |  |  |  | NSAIDs | - | - | - | - | - |  |
|  |  |  |  | Anti-inflammatory corticosteroids | - | - | - | - | -- |  |
|  |  | Beiyun River, Beijing | Nov 2021 | Antibacterials | ERY | ND-29.6 | 4.42 | - | 86.6 | [74] |
|  |  |  |  |  | ROX | ND-47.8 | 12.7 | - | 97.6 |  |
|  |  |  |  |  | CLR | ND-49.5 | 11.8 | - | 67.1 |  |
|  |  |  |  |  | AZM | ND-83.2 | 20.9 | - | 98.8 |  |
|  |  |  |  |  | CPFX | ND-13 | 2.14 | - | 31.7 |  |
|  |  |  |  |  | OFX | <0.16-114 | 18.7 | - | 100 |  |
|  |  |  |  |  | NOR | ND-193 | 40 | - | 90.2 |  |
|  |  |  |  |  | ENR | ND-18.7 | 0.97 | - | 31.7 |  |
|  |  |  |  |  | LIN | ND-45.8 | 5.74 | - | 62.2 |  |
|  |  |  |  |  | AMP | ND | ND | - | 0 |  |
|  |  |  |  | Antiviral drugs | RTV | ND-18.0 | 1.64 | - | 59.8 |  |
|  |  |  |  | NSAIDs | DFC | ND-13 | 2.14 | - | 31.7 |  |
|  |  |  |  | Anti-inflammatory corticosteroids | - | - | - | - | - |  |
|  |  | Tianjin | Jun 2021 | Antibacterials | ERY | 0.25-54 | 9.99 | 8.03 | 100 | [75] |
|  |  |  |  |  | ROX | <LOQ-8.3 | 1.76 | 1.07 | 100 |  |
|  |  |  |  |  | AZM | ND-166 | 6.96 | 0.33 | 94.7 |  |
|  |  |  |  |  | CPFX | 0.35-14.4 | 1.97 | 1.29 | 100 |  |
|  |  |  |  |  | OFX | <LOQ-44.4 | 3.3 | 1.44 | 100 |  |
|  |  |  |  |  | NOR | 1.42-16.6 | 5.76 | 5.1 | 100 |  |
|  |  |  |  | Antiviral drugs | - | - | - | - | - |  |
|  |  |  |  | NSAIDs | - | - | - | - | - |  |
|  |  |  |  | Anti-inflammatory corticosteroids | - | - | - | - | -- |  |
|  |  | Beijing section of Beiyun River | Oct 2022 | Antibacterials | ERY | ND-16.1 | 6.05 | - | 94.7 | [76] |
|  |  |  |  |  | ROX | ND-5.77 | 2.51 | - | 78.9 |  |
|  |  |  |  |  | CLR | 1.26-12.0 | 3.96 | - | 100 |  |
|  |  |  |  |  | AZM | ND-84.1 | 24.2 | - | 97.4 |  |
|  |  |  |  |  | CPFX | ND-8.48 | 2.39 | - | 73.7 |  |
|  |  |  |  |  | OFX | ND-17.0 | 3.84 | - | 68.4 |  |
|  |  |  |  |  | NOR | ND-31.2 | 12.9 | - | 76.3 |  |
|  |  |  |  |  | ENR | ND-3.68 | 0.93 | - | 92.1 |  |
|  |  |  |  |  | AMP | ND | ND | - | 0 |  |
|  |  |  |  |  | LIN | ND-2.91 | 0.42 | - | 65.8 |  |
|  |  |  |  | Antiviral drugs | RTV | 20.5-81.7 | 46.2 | - | 100 |  |
|  |  |  |  |  | LPV | ND-2.46 | 1.04 | - | 97.4 |  |
|  |  |  |  | NSAIDs | DFC | 16.9-43.8 | 28.9 | - | 100 |  |
|  |  |  |  | Anti-inflammatory corticosteroids | - | - | - | - | - |  |
|  | YwRB  YwRB  YwRB | Henan section of the middle and lower Yellow River | Jul-Dec 2021 | Antibacterials | ROX | 0.1240 - 67.64 | 9.626 | 3.781 | 100 | [77] |
|  |  |  |  |  | CLR | ND - 13.6 | 1.377 | 0.5012 | 94 |  |
|  |  |  |  |  | AZM | ND - 56.72 | 2.07 | 0.3179 | 94 |  |
|  |  |  |  |  | NOR | 6.144 - 382.3 | 43.47 | 23.84 | 100 |  |
|  |  |  |  |  | ENR | 8.825 - 206.7 | 27.35 | 18.63 | 100 |  |
|  |  |  |  | Antiviral drugs | - | - | - | - | - |  |
|  |  |  |  | NSAIDs | IBF | 0.7834 - 146.7 | 29.51 | 15.81 | 100 |  |
|  |  |  |  |  | NPX | ND - 17.52 | 3.915 | 2.571 | 94 |  |
|  |  |  |  |  | ATP | 3.15 - 341.6 | 47.15 | 26.03 | 100 |  |
|  |  |  |  |  | IM | ND - 76.59 | 15.87 | 12.08 | 91 |  |
|  |  |  |  | Anti-inflammatory corticosteroids | - | - | - | - | - |  |
|  |  | Yellow River | May 2021 | Antibacterials | ERY | ND-7.14 | 2.29 | - | 87.88 | [78] |
|  |  |  |  |  | CLR | ND | ND | - | 0 |  |
|  |  |  |  |  | NOR | ND-2.13 | 0.41 | - | 39.39 |  |
|  |  |  |  |  | ENR | ND | ND | - | 0 |  |
|  |  |  |  | Antiviral drugs | - | - | - | - | - |  |
|  |  |  |  | NSAIDs | - | - | - | - | - |  |
| During COVID-19 |  |  |  | Anti-inflammatory corticosteroids | - | - | - | - | - |  |
|  |  | Anhui | 2022 | Antibacterials | ERY | ND-0.51 | 0.01 | - | - | [79] |
|  |  |  |  |  | ROX | ND-0.73 | 0.01 | - | - |  |
|  |  |  |  |  | AZM | ND-8.85 | 0.27 | - | - |  |
|  |  |  |  |  | CLR | ND-17.66 | 0.81 | - | - |  |
|  |  |  |  |  | CPFX | ND-0.84 | 0.01 | - | - |  |
|  |  |  |  |  | OFX | ND-0.20 | 0.01 | - | - |  |
|  |  |  |  |  | LVX | ND-0.25 | 0.01 | - | - |  |
|  |  |  |  |  | NOR | ND | ND | - | - |  |
|  |  |  |  |  | ENR | ND-0.11 | 0.01 | - | - |  |
|  |  |  |  |  | LIN | ND-159.38 | 8.02 | - | - |  |
|  |  |  |  |  | CLI | ND-100.45 | 7.38 | - | - |  |
|  |  |  |  |  | ERY | ND-1.72 | 0.06 | - | - |  |
|  |  |  |  |  | ROX | ND | ND | - | - |  |
|  |  |  |  |  | AZM | ND | ND | - | - |  |
|  |  |  |  |  | CLR | ND | ND | - | - |  |
|  |  |  |  |  | CPFX | ND-0.84 | 0.03 | - | - |  |
|  |  |  |  |  | OFX | ND-1.07 | 0.10 | - | - |  |
|  |  |  |  |  | LVX | ND-0.96 | 0.09 | - | - |  |
|  |  |  |  |  | NOR | ND-2.28 | 0.09 | - | - |  |
|  |  |  |  |  | ENR | ND-0.28 | 0.02 | - | - |  |
|  |  |  |  |  | MOX | ND-0.25 | 0.01 | - | - |  |
|  |  |  |  |  | LIN | ND-2.42 | 0.01 | - | - |  |
|  |  |  |  |  | CLI | ND-0.21 | 0.01 | - | - |  |
|  |  |  |  | Antiviral drugs | - | - | - | - | - |  |
|  |  |  |  | NSAIDs | - | - | - | - | - |  |
|  |  |  |  | Anti-inflammatory corticosteroids | - | - | - | - | - |  |
|  |  | Xining urban wetlands on the Qinghai-Tibet Plateau | Mar 2022 | Antibacterials | ERY | 1.04-17 | 9.24 | 7.63 | 44.44 | [80] |
|  |  |  |  |  | ROX | ND-1.62 | 1.62 | 1.62 | 11.11 |  |
|  |  |  |  |  | AZM | 171.34-193.82 | 173.81 | 172.32 | 94.44 |  |
|  |  |  |  |  | CPFX | 1.92-24.5 | 16 | 21.58 | 16.67 |  |
|  |  |  |  |  | OFX | 0.54-1220.86 | 180.21 | 0.54 | 61.11 |  |
|  |  |  |  |  | NOR | 1.9- 520.86 | 56.98 | 6.29 | 100 |  |
|  |  |  |  |  | ENR | 2.46-27.06 | 4.63 | 2.77 | 100 |  |
|  |  |  |  | Antiviral drugs | - | - | - | - | - |  |
|  |  |  |  | NSAIDs | - | - | - | - | - |  |
|  |  |  |  | Anti-inflammatory corticosteroids | - | - | - | - | -- |  |
|  |  | Jiaozhou Bay estuarine region | Jul-Aug 2023 | Antibacterials | ERY | ND−3.61 | 0.368 | - | - | [81] |
|  |  |  |  |  | ROX | ND−43.0 | 5.79 | - | - |  |
|  |  |  |  |  | CPFX | ND | ND | - | - |  |
|  |  |  |  |  | OFX | ND−80.6 | 13.2 | - | - |  |
| During COVID-19 |  |  |  |  | NOR | ND−65.6 | 4.20 | - | - |  |
|  |  |  |  |  | ENR | ND−0.99 | 0.098 | - | - |  |
|  |  |  |  | Antiviral drugs | - | - | - | - | - |  |
|  |  |  |  | NSAIDs | - | - | - | - | - |  |
|  |  |  |  | Anti-inflammatory corticosteroids | - | - | - | - | -- |  |
|  | HuRB  HuRB | Luoma Lake | Apr 2020 | Antibacterials | - | - | - | - | - | [49] |
|  |  |  |  | Antiviral drugs | - | - | - | - | - |  |
|  |  |  |  | NSAIDs | IBF | ND-130.58 | 26.89 | - | 65 |  |
|  |  |  |  |  | NPX | ND-1070.4 | 441.5 | - | 72 |  |
|  |  |  |  |  | KPF | ND-1715.4 | 640.26 | - | 72 |  |
|  |  |  |  |  | IM | ND-197.12 | 73.99 | - | 77 |  |
|  |  |  |  | Anti-inflammatory corticosteroids | - | - | - | - | -- |  |
|  |  | Luoma Lake | Mar-Jul 2020 | Antibacterials | ERY | ND | ND | - | ND | [72] |
|  |  |  |  |  | ROX | ND | ND | - | ND |  |
|  |  |  |  |  | AZM | ND | ND | - | ND |  |
|  |  |  |  |  | CPFX | 0.03-0.09 | 0.06 | - | 100 |  |
|  |  |  |  |  | OFX | 0.00-0.03 | 0.02 | - | 100 |  |
|  |  |  |  |  | NOR | 0.05-0.18 | 0.008 | - | 100 |  |
|  |  |  |  |  | ENR | 0.00-0.04 | 0.003 | - | 100 |  |
|  |  |  |  | Antiviral drugs | - | - | - | - | - |  |
|  |  |  |  | NSAIDs | - | - | - | - | - |  |
|  |  |  |  | Anti-inflammatory corticosteroids | - | - | - | - | -- |  |
|  |  | Anqing city | Oct 2020 | Antibacterials | ERY | 6.35–29.5 | 12.3 | - | 100 | [82] |
|  |  |  |  |  | ROX | ND-1.16 | 1.20 | - | 75 |  |
|  |  |  |  |  | AZM | ND-7.82 | 0.98 | - | 12.5 |  |
|  |  |  |  |  | CPFX | 12.8–99.5 | 42.9 | - | 100 |  |
|  |  |  |  |  | OFX | 6.72–24.5 | 11.1 | - | 100 |  |
|  |  |  |  |  | NOR | 4.16–48.6 | 30.4 | - | 100 |  |
|  |  |  |  |  | ENR | 6.88–43.6 | 17.2 | - | 100 |  |
|  |  |  |  |  | CLI | ND-6.42 | 1.76 | - | 50 |  |
|  |  |  |  | Antiviral drugs | - | - | - | - | - |  |
|  |  |  |  | NSAIDs | - | - | - | - | - |  |
|  |  |  |  | Anti-inflammatory corticosteroids | - | - | - | - | -- |  |
|  |  | Guohe River, Bozhou City | Oct 2022 | Antibacterials | ERY | <LOQ-13.92 | 4.6203 | - | 97.78 | [83] |
|  |  |  |  |  | ROX | <LOQ-16.89 | 5.9785 | - | 97.78 |  |
|  |  |  |  |  | AZM | <LOQ-6.59 | 0.5662 | - | 22.22 |  |
|  |  |  |  |  | CLR | 4.47-7.68 | 5.5522 | - | 100 |  |
|  |  |  |  |  | CPFX | <LOQ-11.12 | 0.6592 | - | 8.89 |  |
|  |  |  |  |  | OFX | <LOQ-16.02 | 0.5366 | - | 8.89 |  |
|  |  |  |  |  | ENR | <LOQ-90.87 | 6.1195 | - | 20 |  |
|  |  |  |  | Antiviral drugs | - | - | - | - | - |  |
| During COVID-19 |  |  |  | NSAIDs | - | - | - | - | - |  |
|  |  |  |  | Anti-inflammatory corticosteroids | - | - | - | - | - |  |
|  | LRB | Dahuofang reservoir | Mar-Jul 2020 | Antibacterials | ERY | 2.10-8.90 | 4.05 | - | 100 | [72] |
|  |  |  |  |  | ROX | 1.90-9.20 | 3.72 | - | 100 |  |
|  |  |  |  |  | AZM | 0.90-2.00 | 1.16 | - | 100 |  |
|  |  |  |  |  | CPFX | ND | ND | - | ND |  |
|  |  |  |  |  | OFX | 0.00-1.80 | 0.27 | - | 90 |  |
|  |  |  |  |  | NOR | ND | ND | - | ND |  |
|  |  |  |  |  | ENR | 0.00-2.60 | 0.74 | - | 100 |  |
|  |  |  |  | Antiviral drugs | - | - | - | - | - |  |
|  |  |  |  | NSAIDs | - | - | - | - | - |  |
|  |  |  |  | Anti-inflammatory corticosteroids | - | - | - | - | -- |  |
|  | SRB  SRB | Songhua Lake | Mar-Jul 2020 | Antibacterials | ERY | 1.2-4.2 | 1.92 | - | 100 |  |
|  |  |  |  |  | ROX | 0.9-2.5 | 1.21 | - | 100 |  |
|  |  |  |  |  | AZM | 0.8-2.00 | 1.10 | - | 100 |  |
|  |  |  |  |  | CPFX | ND | ND | ND | ND |  |
|  |  |  |  |  | OFX | ND | ND | ND | ND |  |
|  |  |  |  |  | NOR | ND | ND | ND | ND |  |
|  |  |  |  |  | ENR | ND | ND | ND | ND |  |
|  |  |  |  | Antiviral drugs | - | - | - | - | - |  |
|  |  |  |  | NSAIDs | - | - | - | - | - |  |
|  |  |  |  | Anti-inflammatory corticosteroids | - | - | - | - | -- |  |
|  |  | Xiaoxingkai Lake | Mar-Jul 2020 | Antibacterials | ERY | 1.70-2.50 | 2.02 | - | 100 |  |
|  |  |  |  |  | ROX | 0.8-1.20 | 0.88 | - | 100 |  |
|  |  |  |  |  | AZM | 1.00-3.90 | 1.54 | - | 100 |  |
|  |  |  |  |  | CPFX | ND | ND | - | ND |  |
|  |  |  |  |  | OFX | 0.00-1.70 | 1.2 | - | 84.62 |  |
|  |  |  |  |  | NOR | ND | ND | - | ND |  |
|  |  |  |  |  | ENR | ND | ND | - | ND |  |
|  |  |  |  | Antiviral drugs | - | - | - | - | - |  |
|  |  |  |  | NSAIDs | - | - | - | - | - |  |
|  |  |  |  | Anti-inflammatory corticosteroids | - | - | - | - | -- |  |
|  |  | Jilin segment of the Songliao basin | Jan and Jul 2022 | Antibacterials | ERY | 6.50-113.30 | 46.25 | 42.08 | - | [84] |
|  |  |  |  |  | ROX | 5.77-143.50 | 46.08 | 23.78 | - |  |
|  |  |  |  |  | CPFX | 24.27-159.38 | 66.20 | 65.95 | - |  |
|  |  |  |  |  | OFX | 1.74-39.8 | 9.57 | 4.47 | - |  |
|  |  |  |  |  | AMP | 0.77-6.44 | 1.8 | 1.65 | - |  |
|  |  |  |  |  | AMX | 58.12-117.76 | 69.41 | 60.94 | - |  |
|  |  |  |  | Antiviral drugs | - | - | - | - | - |  |
|  |  |  |  | NSAIDs | - | - | - | - | - |  |
|  |  |  |  | Anti-inflammatory corticosteroids | - | - | - | - | -- |  |
| Post-COVID-19  Post-COVID-19 | YzRB  YzRB | Chongqing section of Yangtze River | Feb and Sep 2023 | Antibacterials | ERY | 0.94-164.7 | 39.24 | 39.06 | 40.74 | [85] |
|  |  |  |  |  | ROX | 0.46-257.56 | 24.59 | 22.76 | 100 |  |
|  |  |  |  |  | CLR | 0.06-149.54 | 28.76 | 1.71 | 44.44 |  |
|  |  |  |  |  | AZM | 22.5-368.2 | 189.31 | 177.22 | 11.11 |  |
|  |  |  |  |  | CPFX | 6.8-94.92 | 55.65 | 65.22 | 11.11 |  |
|  |  |  |  |  | OFX | 0.02-28.34 | 9,7 | 4.28 | 33.33 |  |
|  |  |  |  |  | LVX | 0-28.02 | 14.39 | 14.41 | 18.52 |  |
|  |  |  |  |  | NOR | 0-59.96 | 9.84 | 0 | 33 |  |
|  |  |  |  |  | ENR | 41.08-48.92 | 44.93 | 10.66 | 11.1 |  |
|  |  |  |  |  | MOX | 1.68-3.36 | 2.3 | 1.36 | 14.81 |  |
|  |  |  |  |  | LIN | 0.1-138.48 | 19.13 | 1.45 | 66.67 |  |
|  |  |  |  |  | CLI | 0.2-90.04 | 12.2 | 2.33 | 96.3 |  |
|  |  |  |  |  | CTX | 7.2-14.66 | 10.8 | 10.66 | 14.81 |  |
|  |  |  |  | Antiviral drugs | - | - | - | - | - |  |
|  |  |  |  | NSAIDs | - | - | - | - | - |  |
|  |  |  |  | Anti-inflammatory corticosteroids | - | - | - | - | -- |  |
|  |  | Dongting Lake | Mar 2023 | Antibacterials | ROX | ND-5.16 | 1.11 | 0.65 | 95.45 | [86] |
|  |  |  |  |  | AZM | ND-1.78 | 0.34 | 0 | 40.91 |  |
|  |  |  |  |  | CPFX | ND-20.5 | 2.7 | 0 | 22.73 |  |
|  |  |  |  |  | CLR | ND-2.10 | 0.68 | 0.44 | 86.36 |  |
|  |  |  |  |  | OFX | ND-53.90 | 3.99 | 1.59 | 50 |  |
|  |  |  |  |  | LVX | ND-55.48 | 3.46 | 0 | 31.82 |  |
|  |  |  |  |  | MOX | ND | ND | ND | ND |  |
|  |  |  |  |  | LIN | ND-17.46 | 4.7 | 2.48 | 68.18 |  |
|  |  |  |  |  | CLI | ND-7.22 | 2.28 | 1.13 | 86.36 |  |
|  |  |  |  |  | CTX | ND-4.26 | 0.37 | 0 | 9.09 |  |
|  |  |  |  | Antiviral drugs | - | - | - | - | - |  |
|  |  |  |  | NSAIDs | - | - | - | - | - |  |
|  |  |  |  | Anti-inflammatory corticosteroids | - | - | - | - | -- |  |
|  |  | The river confluence in Shanghai | Jan 2023 | Antibacterials | ERY | <LOQ-5.68 | 1.96 | - | 100 | [87] |
|  |  |  |  |  | ROX | 0.27-2.31 | 0.65 | - | 100 |  |
|  |  |  |  |  | CPFX | <LOQ-1.35 | 0.79 | - | 100 |  |
|  |  |  |  |  | OFX | 0.46-25.91 | 1.19 | - | 100 |  |
|  |  |  |  |  | NOR | <LOQ-1.95 | 1.03 | - | 100 |  |
|  |  |  |  |  | ENR | <LOQ-0.46 | 0.2 | - | 100 |  |
|  |  |  |  | Antiviral drugs | - | - | - | - | - |  |
|  |  |  |  | NSAIDs | - | - | - | - | - |  |
|  |  |  |  | Anti-inflammatory corticosteroids | - | - | - | - | -- |  |
|  | PRB | Dongjiang River | Mep 2023 | Antibacterials | ERY | 1.96-4.89 | 2.29 | 2.18 | - | [71] |
|  |  |  |  |  | ROX | ND-6.34 | 2.53 | 2.34 | - |  |
|  |  |  |  |  | AZM | 1.09-4.07 | 2.74 | 2.46 | - |  |
|  |  |  |  |  | CPFX | ND-22.02 | 5.38 | 3.25 | - |  |
|  |  |  |  |  | OFX | ND-133.75 | 17.02 | 5.37 | - |  |
|  |  |  |  |  | NOR | ND-76.24 | 23.9 | 22.1 | - |  |
|  |  |  |  |  | ENR | 0.89-3.80 | 1.43 | 1.22 | - |  |
|  |  |  |  |  | AMX | ND-28.04 | 9.35 | 5.85 | - |  |
|  |  |  |  | Antiviral drugs | - | - | - | - | - |  |
|  |  |  |  | NSAIDs | - | - | - | - | - |  |
|  | HuRB |  |  | Anti-inflammatory corticosteroids | - | - | - | - | -- |  |
|  |  | Jiaozhou Bay estuarine region | Jul-Aug 2022 | Antibacterials | ERY | ND−4.65 | 0.362 | - | - | [81] |
|  |  |  |  |  | ROX | ND−30.2 | 3.73 | - | - |  |
|  |  |  |  |  | CPFX | ND-36.4 | 15.2 | - | - |  |
|  |  |  |  |  | OFX | ND-306 | 19.8 | - | - |  |
|  |  |  |  |  | NOR | ND−730 | 41.6 | - | - |  |
|  |  |  |  |  | ENR | ND | ND | - | - |  |
|  |  |  |  | Antiviral drugs | - | - | - | - | - |  |
|  |  |  |  | NSAIDs | - | - | - | - | - |  |
|  |  |  |  | Anti-inflammatory corticosteroids | - | - | - | - | -- |  |
|  | HuRB | Gaoyou Lake | Nov 2023 | Antibacterials | ERY | 5.69-6.77 | 6.12 | - | 100 | [88] |
|  |  |  |  |  | ROX | ND-2.89 | 2.1 | - | 73.3 |  |
|  |  |  |  |  | AZM | 5.48-10.8 | 8.39 | - | 100 |  |
|  |  |  |  |  | CLR | ND-6.26 | 4.85 | - | 86.7 |  |
|  |  |  |  |  | CPFX | ND-6.28 | 3.69 | - | 93.3 |  |
|  |  |  |  |  | NOR | ND-4.07 | 0.27 | - | 6.7 |  |
|  |  |  |  |  | ENR | ND-1.27 | 0.08 | - | 6.7 |  |
|  |  |  |  | Antiviral drugs | - | - | - | - | - |  |
| Post-COVID-19 |  |  |  | NSAIDs | - | - | - | - | - |  |
|  |  |  |  | Anti-inflammatory corticosteroids | - | - | - | - | -- |  |
|  |  | Nanwan reservoir | Jun and Mar 2023 | Antibacterials | ERY | 0.00-1.09 | 0.08 | 0.00 | 23 | [89] |
|  |  |  |  |  | ROX | 0.00-3.79 | 0.20 | 0.00 | 17 |  |
|  |  |  |  |  | CLR | 0.00-0.70 | 0.07 | 0.00 | 29 |  |
|  |  |  |  |  | CPFX | 0.00-1409.00 | 89.86 | 0.00 | 18 |  |
|  |  |  |  |  | OFX | 0.00-326.00 | 32.14 | 0.00 | 45 |  |
|  |  |  |  |  | ENR | 0.00-240.50 | 22.51 | 0.00 | 42 |  |
|  |  |  |  |  | LIN | 0-541.2 | 64.62 | 1.4 | 53 |  |
|  |  |  |  |  | CLI | 0-128.2 | 16.02 | 3.15 | 55 |  |
|  |  |  |  | Antiviral drugs | - | - | - | - | - |  |
|  |  |  |  | NSAIDs | - | - | - | - | - |  |
|  |  |  |  | Anti-inflammatory corticosteroids | - | - | - | - | -- |  |
|  | SRB | Jilin segment of the Songliao basin | Apr 2023 | Antibacterials | ERY | 7.57-141.22 | 40.19 | 27.80 | - | [84] |
|  |  |  |  |  | ROX | 20.87-153.82 | 71.28 | 59.64 | - |  |
|  |  |  |  |  | CPFX | 6.95-54.56 | 18.64 | 12.66 | - |  |
|  |  |  |  |  | OFX | 0.48-9.69 | 2.82 | 1.94 | - |  |
|  |  |  |  |  | AMP | 0.57-6.88 | 3.98 | 4.25 | - |  |
|  |  |  |  |  | AMX | 56.1-57.29 | 56.51 | 57.29 | - |  |
|  |  |  |  | Antiviral drugs | - | - | - | - | - |  |
|  |  |  |  | NSAIDs | - | - | - | - | - |  |
|  |  |  |  | Anti-inflammatory corticosteroids | - | - | - | - | - |  |

References

1. Xie Z, Lu G, Yan Z, Liu J, Wang P, Wang Y. Bioaccumulation and trophic transfer of pharmaceuticals in food webs from a large freshwater lake. Environ Pollut*.* 2017;222:356–366.
2. Zhou H, Ying T, Wang X, Liu J. Occurrence and preliminarily environmental risk assessment of selected pharmaceuticals in the urban rivers, China. Sci Rep. 2016;6:34928.
3. Yan M, Xu C, Huang Y, Nie H, Wang J. Tetracyclines, sulfonamides and quinolones and their corresponding resistance genes in the Three Gorges Reservoir, China. Sci Total Environ. 2018;631-632:840–848.
4. Hu XL, Bao YF, Hu JJ, Liu YY, Yin DQ. Occurrence of 25 pharmaceuticals in Taihu Lake and their removal from two urban drinking water treatment plants and a constructed wetland. Environ. Sci Pollut Res Int. 2017;24:14889–14902.
5. Ma R, Wang B, Lu S, Zhang Y, Yin L, Huang J, et al. Characterization of pharmaceutically active compounds in Dongting Lake, China: Occurrence, chiral profiling and environmental risk. T Sci Total Environ. 2016;557-558:268–275.
6. Tong L, Qin L, Guan C, Wilson ME, Li X, Cheng D, et al. Antibiotic resistance gene profiling in response to antibiotic usage and environmental factors in the surface water and groundwater of Honghu Lake, China. Environ Sci Pollut Res Int. 2020;27:31995–32005.
7. Liang X, Guan F, Chen B, Luo P, Guo C, Wu G, et al. Spatial and seasonal variations of antibiotic resistance genes and antibiotics in the surface waters of Poyang Lake in China. Ecotoxicol Environ Saf. 2020;196:110543.
8. Guo X, Xiao JL, Zhang A, Yan Z, Chen S, Wang N. Antibiotic contamination in a typical water-rich city in southeast China: a concern for drinking water resource safety. J Environ Sci Health B. 2020;55:193–209.
9. Tang J, Sun J, Wang W, Yang L, Xu Y. Pharmaceuticals in two watersheds in Eastern China and their ecological risks. Environ Pollut. 2021**;**277:116773.
10. Sun S, Chen Y, Lin Y, An D. Occurrence, spatial distribution, and seasonal variation of emerging trace organic pollutants in source water for Shanghai, China. Sci Total Environ. 2018; 639:1–7.
11. Liu S, Wang C, Wang P, Chen J, Wang X, Yuan Q. Anthropogenic disturbances on distribution and sources of pharmaceuticals and personal care products throughout the Jinsha River Basin, China. Environ Res. 2021;198:110449.
12. Wang Y, Liu Y, Lu S, Liu X, Meng Y, Zhang G, et al. Occurrence and ecological risk of pharmaceutical and personal care products in surface water of the Dongting Lake, China-during rainstorm period. Environ. Sci Pollut Res Int. 2019;26:28796–28807.
13. Lin H, Chen L, Li H, Luo Z, Lu J, Yang Z. Pharmaceutically active compounds in the Xiangjiang River, China: Distribution pattern, source apportionment, and risk assessment. Sci Total Environ*.* 2018;636:975–984.
14. Liu Y, Feng M, Wang B, Zhao X, Guo R, Bu Y, et al. Distribution and potential risk assessment of antibiotic pollution in the main drinking water sources of Nanjing, China.  Environ Sci Pollut Res Int. 2020;27:21429–21441.
15. Cao SS, Duan YP, Tu YJ, Tang Y, Liu J, Zhi WD, et al. Pharmaceuticals and personal care products in a drinking water resource of Yangtze River Delta Ecology and Greenery Integration Development Demonstration Zone in China: Occurrence and human health risk assessment. Sci Total Environ. 2020;721:137624.
16. Wang G, Zhou S, Han X, Zhang L, Ding S, Li Y, et al. Occurrence, distribution, and source track of antibiotics and antibiotic resistance genes in the main rivers of Chongqing city, Southwest China. J Hazard Mater*.* 2020;389:122110.
17. Zhang G, Lu S, Wang Y, Liu X, Liu Y, Xu J, et al. Occurrence of antibiotics and antibiotic resistance genes and their correlations in lower Yangtze River, China. Environ Pollut. 2020;257:113365.
18. Jiang X, Zhu Y, Liu L, Fan X, Bao Y, Deng S, et al. Occurrence and variations of pharmaceuticals and personal-care products in rural water bodies: A case study of the Taige Canal (2018-2019). Sci Total Environ. 2021;762:143138.
19. An W, Duan L, Zhang Y, Zhou Y, Wang B, Yu G. Pollution characterization of pharmaceutically active compounds (PhACs) in the northwest of Tai Lake Basin, China: Occurrence, temporal changes, riverine flux and risk assessment. J Hazard Mater. 2022;422:126889.
20. Huang J, Ding J, Jiang H, Wang Z, Zheng L, Song X, et al. Pharmaceuticals and Personal Care Products across Different Water Bodies in Taihu Lake Basin, China: Occurrence, Source, and Flux. Int J Environ Res Public Health. 2022;19:11135.
21. Zhu F, Wang S, Liu Y, Wu M, Wang H, Xu G. Antibiotics in the surface water of Shanghai, China: screening, distribution, and indicator selecting. Environ Sci Pollut Res Int. 2021;28:9836–9848.
22. Li Y, Tong L, Zhang J, Liu H, Li M, Wen Z. Distribution and risk assessment of antibiotics under water level fluctuation in the riparian zone of the Hanjiang River. Ecotoxicol Environ Saf. 2023;256:114833.
23. Liu Y, Chen Y, Feng M, Chen J, Shen W, Zhang S. Occurrence of antibiotics and antibiotic resistance genes and their correlations in river-type drinking water source, China. Environ. Sci Pollut Res Int. 2021;28:42339–42352.
24. Peng FJ, Pan CG, Zhang M, Zhang NS, Windfeld R, Salvito D, et al. Occurrence and ecological risk assessment of emerging organic chemicals in urban rivers: Guangzhou as a case study in China. Sci Total Environ. 2017;589:46–55.
25. Cui Y, Wang Y, Pan C, Li R, Xue R, Guo J, et al. Spatiotemporal distributions, source apportionment and potential risks of 15 pharmaceuticals and personal care products (PPCPs) in Qinzhou Bay, South China. Mar Pollut Bull. 2019;141,104–111.
26. Gong J, Lin C, Xiong X, Chen D, Chen Y, Zhou Y, et al. Occurrence, distribution, and potential risks of environmental corticosteroids in surface waters from the Pearl River Delta, South China. Environ Pollut. 2019;251:102–109.
27. Wang C, Zhao Y, Liu S, Xiao Q, Liang W, Song Y. Contamination, distribution, and risk assessment of antibiotics in the urban surface water of the Pearl River in Guangzhou, South China. Environ Monit Assess. 2021;193:98.
28. Zhao B, Xu J, Zhang G, Lu S, Liu X, Li L, et al. Occurrence of antibiotics and antibiotic resistance genes in the Fuxian Lake and antibiotic source analysis based on principal component analysis-multiple linear regression model. Chemosphere. 2021;262:127741.
29. Li W, Gao L, Shi Y, Liu J, Cai Y. Occurrence, distribution and risks of antibiotics in urban surface water in Beijing, China. Environ Sci: Processes Impacts. 2015;17:1611–1619.
30. Ma R, Wang B, Yin L, Zhang Y, Deng S, Huang J, et al. Characterization of pharmaceutically active compounds in Beijing, China: Occurrence pattern, spatiotemporal distribution and its environmental implication. J Hazard Mater. 2017;323:147–155.
31. Lei K, Zhu Y, Chen W, Pan HY, Cao YX, Zhang X, et al. Spatial and seasonal variations of antibiotics in river waters in the Haihe River Catchment in China and ecotoxicological risk assessment. Environ Int. 2019;130:104919.
32. Duan L, Zhang Y, Wang B, Cagnetta G, Deng S, Huang J, et al. Characteristics of pharmaceutically active compounds in surface water in Beijing, China: Occurrence, spatial distribution and biennial variation from 2013 to 2017.  Environ Pollut. 2020;264:114753.
33. Zhang Y, Chen H, Jing L, Teng Y. Ecotoxicological risk assessment and source apportionment of antibiotics in the waters and sediments of a peri-urban river. Sci Total Environ. 2020;731:139128.
34. Fang L, Wang L, Chen W, Sun J, Wang L. Identifying the impacts of natural and human factors on ecosystem service in the yangtze and yellow river basins. J Clean Prod. 2021;314:127995.
35. Zhang P, Zhou H, Li K, Zhao X, Liu Q, Li D, et al. Occurrence of pharmaceuticals and personal care products, and their associated environmental risks in Guanting Reservoir and its upstream rivers in north China. RSC Adv. 2018;8:4703–4712.
36. Zhang P, Zhou H, Li K, Zhao X, Liu Q, Li D, et al. Occurrence of pharmaceuticals and personal care products, and their associated environmental risks in a large shallow lake in north China. Environ Geochem Health. 2018;40:1525–1539.
37. Liu X, Zhang G, Liu Y, Lu S, Qin P, Guo X, et al. Occurrence and fate of antibiotics and antibiotic resistance genes in typical urban water of Beijing, China.  Environ Pollut. 2019;246:163–173.
38. Zhang L, Shen L, Qin S, Cui J, Liu Y. Quinolones antibiotics in the Baiyangdian Lake, China: Occurrence, distribution, predicted no-effect concentrations (PNECs) and ecological risks by three methods. Environ Pollut. 2020**;**256:113458.
39. Meng Y, Zhang J, Fiedler H, Liu W, Pan T, Cao Z, et al. Influence of land use type and urbanization level on the distribution of pharmaceuticals and personal care products and risk assessment in Beiyun River, China. Chemosphere. 2022;287:132075.
40. Yang L, Wang T, Zhou Y, Shi B, Bi R, Meng J. Contamination, source and potential risks of pharmaceuticals and personal products (PPCPs) in Baiyangdian Basin, an intensive human intervention area, China. Sci Total Environ. 2021;760:144080.
41. Fu C, Xu B, Chen H, Zhao X, Li G, Zheng Y, et al. Occurrence and distribution of antibiotics in groundwater, surface water, and sediment in Xiong'an New Area, China, and their relationship with antibiotic resistance genes. Sci Total Environ. 2022;807:151011.
42. Feng J, Liu Q, Ru X, Xi N, Sun J. Occurrence and distribution of priority pharmaceuticals in the Yellow River and the Huai River in Henan, China. Environ. Sci Pollut Res Int. 2020;27:16816–16826.
43. Wang J, Wei H, Zhou X, Li K, Wu W, Guo M. Occurrence and risk assessment of antibiotics in the Xi'an section of the Weihe River, northwestern China. Mar Pollut Bull. 2019;146:794–800.
44. Kuang Y, Guo X, Hu J, Li S, Zhang R, Gao Q, et al. Occurrence and risks of antibiotics in an urban river in northeastern Tibetan Plateau. Sci Rep. 2020;10:20054.
45. Lu S, Lin C, Lei K, Wang B, Xin M, Gu X, et al. Occurrence, spatiotemporal variation, and ecological risk of antibiotics in the water of the semi-enclosed urbanized Jiaozhou Bay in eastern China. Water Research. 2020;184:116187.
46. Lu S, Wang J, Wang B, Xin M, Lin C, Gu X, et al. Comprehensive profiling of the distribution, risks and priority of pharmaceuticals and personal care products: A large-scale study from rivers to coastal seas. Water Res. 2023;230,119591.
47. Wang L, Wang Y, Li H, Zhu Y, Liu R. Occurrence, source apportionment and source-specific risk assessment of antibiotics in a typical tributary of the Yellow River basin. J Environ Manage. 2022;305:114382.
48. Wu Y, Song S, Chen X, Shi Y, Cui H, Liu Y, et al. Source-specific ecological risks and critical source identification of PPCPs in surface water: Comparing urban and rural areas. Sci Total Environ. 2023;854:158792.
49. Wang N, Kang G, Hu G, Chen J, Qi D, Bi F, et al. Spatiotemporal distribution and ecological risk assessment of pharmaceuticals and personal care products (PPCPs) from Luoma Lake, an important node of the South-to-North Water Diversion Project. Environ Monit Assess. 2023;195:1330.
50. Kong M, Bu YQ, Zhang Q, Zhang SH, Xing LQ, Gao ZQ, et al. Distribution, abundance, and risk assessment of selected antibiotics in a shallow freshwater body used for drinking water, China. J Environ Manage. 2021;280:111738.
51. Guo H, Zhao F, Li R, Jin SC, Zhang HB, Zhang KY, et al. Occurrence and distribution of antibiotics and antibiotic resistance genes in water of Liaohe River Basin, China. J Environ Chem Eng. 2022;10:108297.
52. Wang W, Wang H, Zhang W, Liang H, Gao D. Occurrence, distribution, and risk assessment of antibiotics in the Songhua River in China. Environ Sci Pollut Res Int. 2017**;**24:19282–19292.
53. Zhang L, Du S, Zhang X, Lyu G, Dong D, Hua X, et al. Occurrence, distribution, and ecological risk of pharmaceuticals in a seasonally ice-sealed river: From ice formation to melting.  J Hazard Mater. 2020;389:122083.
54. Chen X, Lei L, Liu S, Han J, Li R, Men J, et al. Occurrence and risk assessment of pharmaceuticals and personal care products (PPCPs) against COVID-19 in lakes and WWTP-river-estuary system in Wuhan, China. Sci Total Environ. 2021;792:148352.
55. Zhang Z, Zhou Y, Han L, Guo X, Wu Z, Fang J, et al. Impacts of COVID-19 pandemic on the aquatic environment associated with disinfection byproducts and pharmaceuticals. Sci Total Environ. 2022;811:151409.
56. Mu Y, Tang B, Cheng X, Fu Y, Huang W, Wang J, et al. Source apportionment and predictable driving factors contribute to antibiotics profiles in Changshou Lake of the Three Gorges Reservoir area, China.  J Hazard Mater. 2024;466:133522.
57. Zho Q, Liu G, Arif M, Shi X, Wang S. Occurrence and risk assessment of antibiotics in the surface water of Chaohu Lake and its tributaries in China. Sci Total Environ. 2022;807:151040.
58. Linghu K, Wu Q, Zhang J, Wang Z, Zeng J, Gao S, et al. Occurrence, distribution and ecological risk assessment of antibiotics in Nanming river: Contribution from wastewater treatment plant and implications of urban river syndrome. Process Saf Environ Prot. 2023;169:428-436.
59. Wu T, Zhang Y, Wang B, Chen C, Cheng Z, Li Y, et al. Antibiotic resistance genes in Chishui River, a tributary of the Yangtze River, China: Occurrence, seasonal variation and its relationships with antibiotics, heavy metals and microbial communities. Sci Total Environ. 2022;846:157472.
60. Chen M, Hong Y, Jin X, Guo C, Zhao X, Liu N, et al. Ranking the risks of eighty pharmaceuticals in surface water of a megacity: A multilevel optimization strategy.  Sci Total Environ. 2023;878:163184.
61. Chen M, Jin X, Liu Y, Guo L, Ma Y, Guo C, et al. Human activities induce potential aquatic threats of micropollutants in Danjiangkou Reservoir, the largest artificial freshwater lake in Asia. Sci Total Environ. 2022;850:157843.
62. Jiang L, Zhai W, Wang J, Li G, Zhou Z, Li B, et al. Antibiotics and antibiotic resistance genes in the water sources of the Wuhan stretch of the Yangtze River: Occurrence, distribution, and ecological risks. Environ Res. 2023;231:117295.
63. Yin C, Tan Y, Chen Y, Gao S, Wu M, Zhang Z. Mass load and source apportionment of pharmaceutical and personal care product in the Wuhan section of the Yangtze River, China. Sci Total Environ. 2025;959:178222.
64. Wang N, Wang N, Qi D, Kang G, Wang W, Zhang C, et al. Comprehensive overview of antibiotic distribution, risk and priority: A study of large-scale drinking water sources from the lower Yangtze River. J Environ Manage. 2023;344:118705.
65. Li Y, Wang J, Lin C, Lian M, Wang A, He M, et al. Riverine antibiotic occurrence and potential ecological risks in a low-urbanized and rural basin of the middle Yangtze River: Socioeconomic, land use, and seasonal effects. Environ Res*.* 2023;228:115827.
66. Zeng Y, Duan L, Xu T, Hou P, Zhang X, Wang L, et al. Occurrence and risk assessment of antibiotics in urban river–wetland–lake systems in southwest China. Water. 2024;16(8):1124.
67. Ding F, Li Y, He T, Wang Y, Li Y, Huang Y, et al. Land use and spatial contiguity are key driven factors of antibiotic multimedia patterns in the megacity river network. Sci Total Environ. 2024;947:174727.
68. Zhang Y, Li J, Wu T, Ma K, Cheng Z, Yi Q, et al. Characteristics of antibiotic resistance genes and microbial community distribution in Wanfeng Lake, upper Pearl River, China. Environ Sci Pollut Res Int. 2023;30:83214–83230.
69. Wu H, Bin L, Guo P, Zhao Y, Chen C, Chen Z, et al. Ecological risk assessment of the typical anti-epidemic drugs in the Pearl River Delta by tracing their source and residual characteristics. J Hazard Mater. 2024;463:132914.
70. Jiang S, Shi B, Zhu D, Cheng X, Zhou Z, Xie J, et al. Cross-contamination and ecological risk assessment of antibiotics between rivers and surrounding open aquaculture ponds. Environ Pollut. 2024;344:123404.
71. Zhang H, Ouyang W, Lin C, Wang L, Guo Z, Pei J, et al. Anthropogenic activities drive the distribution and ecological risk of antibiotics in a highly urbanized river basin. Sci Total Environ. 2024;938:173596.
72. Qadeer A, Rui G, Yaqing L, Ran D, Liu C, Jing D, et al. A mega study of antibiotics contamination in Eastern aquatic ecosystems of China: occurrence, interphase transfer processes, ecotoxicological risks, and source modeling. J Hazard Mater. 2023;458:131980.
73. Chen Y, Tan Y, Wang Y, Ma Y, Li P, Du Z, et al. Estimating Sources, Fluxes, and Ecological Risks of Antibiotics in the Wuhan Section of the Yangtze River, China: A Year-Long Investigation. Environ Toxicol Chem. 2023;42:605–619.
74. Huangfu Y, Li Q, Yang W, Bu Q, Yang L, Tang J, et al. Occurrence, Source Apportionment, and Ecological Risk of Typical Pharmaceuticals in Surface Waters of Beijing, China.  Toxics. 2024;12:171.
75. Wu X, Yuan Z, Wang S. Prioritization, sources, and ecological risk of typical antibiotics in the Huai River, a Chinese major river: a warning about aquaculture. Environ Sci Pollut Res Int. 2023;30:64254–64264.
76. Huangfu Y, Shi Y, Yang W, Chang G, Li Q, Gao X, et al. Exploring the occurrence, spatial distribution, and ecological risk of pharmaceuticals in surface water from an urban catchment of Beijing based on DGT in situ measurement. ACS ES&T Water. 2025;5:3205–3215.
77. Yu X, Yu F, Li Z, Zhan J. Occurrence, distribution, and ecological risk assessment of pharmaceuticals and personal care products in the surface water of the middle and lower reaches of the Yellow River (Henan section). J Hazard Mater. 2023;443:130369.
78. Su Z, Wang K, Yang F, Zhuang T. Antibiotic pollution of the Yellow River in China and its relationship with dissolved organic matter: Distribution and Source identification. Water Research. 2023;235:119867.
79. Tian B, Zhang M, Zhu C, Yang R, Yin G, Hu S, et al. Contrastive cognition into the occurrence, source identification and risk assessment of antibiotics in various drinking water sources. Environ Pollut. 2025;374:126226.
80. Zhang F, Mao X, Song X, Yu H, Yan J, Kong D, et al. Ecological Risks of Antibiotics in Urban Wetlands on the Qinghai-Tibet Plateau, China. Int J Environ Res Public Health. 2023;20:1735.
81. Zhang Y, Liu L, Liu Y, Chen L, Wang J, Li Y, et al. Deciphering the natural and anthropogenic drivers on the fate and risk of antibiotics and antibiotic resistance genes (ARGs) in a typical river-estuary system, China. J Hazard Mater. 2024;480:136006.
82. Chen H, Zheng W, Shen X, Zhang F, Zhou X, Shen J, et al. Occurrence, Distribution, and Ecological Risk Assessment of Antibiotics in Different Environmental Media in Anqing, Anhui Province, China. Int J Environ Res Public Health. 2021;18:8112.
83. Wu H, Liu R, Liu G, He M, Arif M, Li F, et al. Unveiling antibiotic contamination in surface water: A study of the Huaihe River Basin's Huaibei Plain, a significant Chinese herbal medicine planting region. Sci Total Environ. 2024;933:173125.
84. Chen A, Zhang T, Cheng F, Yang H, Guo Z, Zhao S, et al. Comprehensive analysis and risk assessment of Antibiotic contaminants, antibiotic-resistant bacteria, and resistance genes: Patterns, drivers, and implications in the Songliao Basin. Environ Pollut. 2024;361:124852.
85. Zhao J, Guo C, Yang Q, Liu W, Zhang H, Luo Y, et al. Comprehensive monitoring and prioritizing for contaminants of emerging concern in the Upper Yangtze River, China: An integrated approach. J Hazard Mater*.* 2024;480:135835.
86. Luo Y, Jin X, Zhao J, Xie H, Guo X, Huang D, et al. Ecological implications and drivers of emerging contaminants in Dongting Lake of Yangtze River Basin, China: A multi-substance risk analysis. J Hazard Mater. 2024;472:134519.
87. Ding F, Li Y, He T, Wang Y, Li Y, Huang Y, et al. Land use and spatial contiguity are key driven factors of antibiotic multimedia patterns in the megacity river network. Sci Total Environ. 2024;947:174727.
88. Xu X, Yang C, Zou L, Leng J, Wang N, Zhang J. Occurrence, distribution, and ecological risks of antibiotics and antibiotic resistance genes in the surface waters of Gaoyou Lake, China. Environ Monit Assess. 2024;196:967.
89. Zhao J, Hou S, Zhang H, Sun S, Guo C, Zhang X, et al. Spatiotemporal variations and priority ranking of emerging contaminants in nanwan reservoir: A case study from the agricultural region in huaihe river basin in China. J Environ Manage. 2024;368:122195.
