# Supplementary material for: Occurrence of antibacterials, antivirals, and anti-inflammatory pharmaceuticals for COVID-19 treatment as emerging contaminants in the Chinese freshwater environment before, during and after the pandemic: the need for dynamic eco-pharmacovigilance
Source: Environ Health Prev Med. 2026 Jul 3;31:44. doi: 10.1265/ehpm.25-00395 (PMC13366171; doi:10.1265/ehpm.25-00395)
Supplement: Supplementary file 2 — Additional file 2: Table S1 Occurrence of anti-COVID-19 drugs in surface freshwater samples collected from the YzRB, China. Table S2 Occurrence of anti-COVID-19 drugs in surface freshwater samples collected from the PRB, China. Table S3 Occurrence of anti-COVID-19 drugs in surface freshwater samples collected from the YwRB, China. [file ehpm-31-044-s002.docx]

**Table S1** Occurrence of anti-COVID-19 drugs in surface freshwater samples collected from the YzRB, China.

| **Group** | **PiE** | **Before the pandemic** | | | **During the pandemic** | | | **After the pandemic** | | |
| --- | --- | --- | --- | --- | --- | --- | --- | --- | --- | --- |
|  |  | **Range** (ng/L) | **Mean/**  **Median** (ng/L) | **Reference** | **Range** (ng/L) | **Mean/**  **Median** (ng/L) | **Reference** | **Range** (ng/L) | **Mean/**  **Median** (ng/L) | **Reference** |
| Antibacterials | ERY | ND-15 | -/1.5 | [1] | <LOQ-23.3 | 2.01/- | [2] | <LOQ-5.68 | 1.96/- | [3] |
|  |  | 12.1-72.6 | 38.6/35.5 | [4] | 0.91-12.9 | -/1.15 | [5] | 0.94-164.7 | 39.24/39.06 | [6] |
|  |  | <LOQ-131.2 | 24.7/- | [7] | <LOQ-14.74 | 7.97/6.34 | [8] |  |  |  |
|  |  | ND-<LOQ | - | [9] | ND-200 | 7.01/ND | [10] |  |  |  |
|  |  | 0.1-8.5 | 1.3/- | [11] | ND-4.84 | 0.9/- | [12] |  |  |  |
|  |  | ND-1.12 | 0.15/ND | [13] | 0.10-9.98 | 0.94/0.17 | [14] |  |  |  |
|  |  | ND-0.78 | 0.09/- | [15] | ND-0.46 | 0.13/- | [16] |  |  |  |
|  |  | ND-1,490 | 263.72/- | [17] | ND-2.58 | - | [18] |  |  |  |
|  |  | ND-30 | 4.67/- | [19] | <LOQ-8.59 | 4.45/3.26 | [8] |  |  |  |
|  |  | 6.57-34.3 | 17.8/- | [20] | 12.6-48.9 | 33.8/31.9 | [21] |  |  |  |
|  |  | ND-5.68 | 0.45/0.07 | [22] | 0.24-3.33 | 0.65/- | [3] |  |  |  |
|  |  | 0.1-5.32 | 1.43/1.03 | [23] |  |  |  |  |  |  |
|  |  | 0.2-4.4 | - | [24] |  |  |  |  |  |  |
|  |  | <LOQ-43 | -/9.5 | [25] |  |  |  |  |  |  |
|  |  | 3.3-58 | - | [26] |  |  |  |  |  |  |
|  |  | ND | - | [27] |  |  |  |  |  |  |
|  | ROX | <LOQ-26 | -/8.1 | [1] | <LOQ-26.78 | 2.04/- | [27] | ND-5.16 | 1.11/0.65 | [28] |
|  |  | <LOQ-18.4 | 14.4/15.5 | [4] | <LOQ-21.92 | 8.58/3.06 | [8] | 0.27-2.31 | 0.65/- | [3] |
|  |  | <LOQ-2.3 | 1.1/- | [7] | 0-10.74 | 0.60/0 | [14] | 0.46-257.56 | 24.59/22.76 | [6] |
|  |  | ND-29.3 | - | [9] | ND-0.4 | 2.6/0.7 | [29] |  |  |  |
|  |  | 0.1-47.5 | 14.1/- | [11] | 0.86-469.30 | 32.61/- | [16] |  |  |  |
|  |  | ND-18.45 | 1.04/0.68 | [13] | ND-18.06 | - | [18] |  |  |  |
|  |  | ND-4.87 | 1.06/- | [15] | <LOQ-10.32 | 5.73/2.03 | [8] |  |  |  |
|  |  | 2.63-41.22 | 4.65/- | [17] | 0.219-0.892 | 0.5/0.46 | [21] |  |  |  |
|  |  | ND-1.75 | 0.44/- | [19] | 0.25-1.01 | 0.36/- | [3] |  |  |  |
|  |  | 0.505-44.6 | 7.22/- | [20] | ND-2.06 | 1.32/- | [30] |  |  |  |
|  |  | ND-29.8 | 2.36/0.9 | [22] |  |  |  |  |  |  |
|  |  | ND-13.3 | 1.9/0.4 | [29] |  |  |  |  |  |  |
|  |  | 0.29-24.55 | 2.75/1.11 | [23] |  |  |  |  |  |  |
|  |  | ND-3.1 | - | [24] |  |  |  |  |  |  |
|  |  | 1.4-190 | 12/6.1 | [25] |  |  |  |  |  |  |
|  |  | ND | - | [26] |  |  |  |  |  |  |
|  |  | ND-22.36 | 2.76/- | [30] |  |  |  |  |  |  |
|  |  | 0.53-1.74 | 1.05/- | [27] |  |  |  |  |  |  |
|  | CLR | ND-48 | 12/- | [31] | <LOQ-49.72 | 2.66/- | [2] | ND-2.10 | 0.68/0.44 | [28] |
|  |  | <LOQ-1.6 | 1.1/- | [7] | 0.18-266 | -/5.93 | [5] | 0.06-149.54 | 28.76/1.71 | [6] |
|  |  | ND-3.42 | 0.55/0.57 | [13] | 0-6.78 | 0.35/0 | [14] |  |  |  |
|  |  | ND-1.72 | 0.42/- | [15] | ND-8.1 | 1/0.1 | [29] |  |  |  |
|  |  | ND-2.19 | 0.26/- | [19] | 2.48-35.32 | - | [18] |  |  |  |
|  |  | 0.146-8.42 | 1.74/- | [20] | 9.88-53.9 | 1.54/- | [30] |  |  |  |
|  |  | ND-12.9 | 2.61/0.3 | [22] |  |  |  |  |  |  |
|  |  | ND-28.3 | 0.2/0.2 | [29] |  |  |  |  |  |  |
|  |  | 0.06-34.90 | 2.08/0.69 | [23] |  |  |  |  |  |  |
|  |  | ND-0.9 | - | [24] |  |  |  |  |  |  |
|  |  | 0.55-100 | 7.9/2.8 | [25] |  |  |  |  |  |  |
|  |  | ND | - | [26] |  |  |  |  |  |  |
|  |  | ND-2.93 | 0.46/- | [30] |  |  |  |  |  |  |
|  |  | ND-19.28 | 2.12/- | [27] |  |  |  |  |  |  |
|  | AZM | <LOQ-1.5 | 0.6/- | [7] | <LOQ-47.34 | 2.55/- | [2] | ND-1.78 | 0.34/0 | [28] |
|  |  | ND-67 | 17/- | [31] | 3.14-935 | -/0.49 | [5] | 22.5-368.2 | 189.31/177.22 | [6] |
|  |  | 0.13-12.5 | 1.87/0.68 | [23] | <LOQ-19.75 | 5.7/4.36 | [8] |  |  |  |
|  |  | ND-0.6 | - | [24] | ND-22.76 | - | [18] |  |  |  |
|  |  | ND-2.32 | 0.41/- | [32] | <LOQ-3.73 | 2.21/1.36 | [8] |  |  |  |
|  |  | 2.2-99 | 8.8/4.7 | [25] | ND | - | [30] |  |  |  |
|  |  | 2.69-9.12 | 4.43/- | [30] |  |  |  |  |  |  |
|  | CPFX | 2.9-43 | -/9.5 | [1] | <LOQ-4.99 | 1.75/- | [2] | ND-20.5 | 2.7/0 | [28] |
|  |  | 1.9-16.6 | 5.2/8.4 | [4] | ND | - | [5] | <LOQ-1.35 | 0.79/- | [3] |
|  |  | <LOQ-48.7 | 33.7/- | [7] | <LOQ-7.25 | 3.41/2.99 | [8] | 6.8-94.92 | 55.65/65.22 | [6] |
|  |  | ND-5.5 | 0.2/ND | [13] | ND- 187.23 | 13.5/ND | [10] |  |  |  |
|  |  | 12.98-14.82 | 13.88/- | [17] | ND-6.62 | 2.17/- | [12] |  |  |  |
|  |  | ND-0.94 | 0.34/- | [19] | 17.3-2,717.31 | - | [18] |  |  |  |
|  |  | 7.1-119 | 38.2/- | [20] | <LOQ | <LOQ | [8] |  |  |  |
|  |  | 0.88-20.76 | 12.9/15.92 | [23] | 2.21 - 5.50 | 3.48/3.22 | [21] |  |  |  |
|  |  | ND | - | [24] | <LOQ-5.07 | 0.96/- | [3] |  |  |  |
|  |  | ND | - | [25] | ND-34.66 | 6.82/- | [30] |  |  |  |
|  |  | ND | - | [26] |  |  |  |  |  |  |
|  | OFX | 10.81-105.23 | 31.12/- | [33] | <LOQ-5.25 | 0.81/- | [2] | ND-20.5 | 3.99/1.59 | [28] |
|  |  | <LOQ-5.7 | 2.9/2.4 | [4] | 39-172 | -/106 | [5] | 0.46-25.91 | 1.19/- | [3] |
|  |  | 0.1-1.1 | 0.2/- | [11] | <LOQ-6.08 | 3.76/3.08 | [8] | 0.02-28.34 | 9.7/4.28 | [6] |
|  |  | ND-13.1 | 0.56/ND | [13] | ND-55.10 | 4.55/ND | [10] |  |  |  |
|  |  | 6.7-117.71 | 100/- | [17] | 43.21-170.26 | 75.74/- | [12] |  |  |  |
|  |  | ND-0.82 | 0.32/- | [19] | 0-130.31 | 5.64/0 | [14] |  |  |  |
|  |  | 1.04-14.3 | 4.71/- | [20] | 20.1-1,395.9 | 259.36/- | [16] |  |  |  |
|  |  | 0.5-177.00 | 13.15/6.43 | [23] | 8..04- 493.73 | - | [18] |  |  |  |
|  |  | ND-12.2 | - | [24] | <LOQ | <LOQ | [8] |  |  |  |
|  |  | ND-23 | <LOQ | [25] | ND - 34.8 | 4.45/0.399 | [21] |  |  |  |
|  |  | ND | - | [26] | 0.66-21.22 | 1.25/- | [3] |  |  |  |
|  |  | ND-9.12 | 1.54/- | [30] | ND-5.47 | 2.43/3.68 | [34] |  |  |  |
|  |  | ND-35.2 | 3.14/- | [27] | ND-4.55 | 1.72/1.32 | [34] |  |  |  |
|  |  |  |  |  | ND-645.1 | 136.6/88.8 | [35] |  |  |  |
|  | LVX |  |  |  |  |  |  | ND-55.48 | 3.46/0 | [28] |
|  |  |  |  |  |  |  |  | 0-28.02 | 14.39/14.41 | [6] |
|  | NOR | 5.2-45 | -/19 | [1] | <LOQ-4.23 | 1.12/- | [2] | <LOQ-1.95 | 1.03/- | [3] |
|  |  | ND | - | [33] | 0.49-4.51 | -/2.5 | [5] | 0-59.96 | 9.84/0 | [6] |
|  |  | 9.2-13.2 | 11/11.1 | [4] | <LOQ-5.51 | 2.96/1.99 | [8] |  |  |  |
|  |  | <LOQ-53.5 | 51.9/- | [7] | ND-80.63 | 1.75/ND | [10] |  |  |  |
|  |  | ND-24.77 | 0.87/ND | [13] | ND-15.22 | 0.61/- | [12] |  |  |  |
|  |  | 16.27-26.52 | 19.32/- | [17] | 89.7- 2,178.01 | - | [18] |  |  |  |
|  |  | ND-0.82 | 0.32/- | [19] | <LOQ-5.47 | 2.3/0.98 | [8] |  |  |  |
|  |  | 1.47-85.4 | 13/- | [20] | 3.81 - 12.6 | 6.44/6.1 | [21] |  |  |  |
|  |  | 14.81-78.68 | 32.53/30.09 | [23] | <LOQ-3.52 | 1.38/- | [3] |  |  |  |
|  |  | ND | - | [24] | ND | - | [30] |  |  |  |
|  |  | ND | - | [25] | ND-285.4 | 36.6/17.5 | [35] |  |  |  |
|  |  | ND | - | [26] |  |  |  |  |  |  |
|  |  | 146.72-290.2 | 180.93/- | [30] |  |  |  |  |  |  |
|  | ENR | 9.04-27.36 | 19.32/- | [33] | <LOQ-20.41 | 3.08/- | [2] | <LOQ-0.46 | 0.2/- | [3] |
|  |  | <LOQ-39.2 | 16/9.7 | [4] | ND | - | [5] | 41.08-48.92 | 44.93/10.66 | [6] |
|  |  | <LOQ-52.1 | 43.6/- | [7] | <LOQ-1.32 | 1.06/0.85 | [8] |  |  |  |
|  |  | ND-1.08 | - | [9] | ND-8.66 | 4.76/- | [12] |  |  |  |
|  |  | ND-7.85 | 1.52/1.33 | [13] | 0-0.25 | 0.03/0 | [14] |  |  |  |
|  |  | 9.85-10.54 | 10.14/- | [17] | 0.42-1.44 | 0.53/- | [16] |  |  |  |
|  |  | ND-0.89 | 0.4/- | [19] | 7.06-81.74 | - | [18] |  |  |  |
|  |  | 0.258-23.6 | 3.57/- | [20] | <LOQ-4.08 | 2.59/<LOQ | [8] |  |  |  |
|  |  | 1.00-10.94 | 6.24/7.01 | [23] | 0.473 - 3.19 | 1.25/1.25 | [21] |  |  |  |
|  |  | ND-2 | - | [24] | 0.55-1.11 | 0.2/- | [3] |  |  |  |
|  |  | ND | - | [25] | 4.25-20.51 | 11.17/- | [30] |  |  |  |
|  |  | 11.53-26.24 | 20.28/- | [30] |  |  |  |  |  |  |
|  |  | ND-7.04 | 0.44/- | [27] |  |  |  |  |  |  |
|  | MOX | 1.64-30.21 | 10.45/8.76 | [23] | ND | - | [5] | ND | - | [28] |
|  |  | ND | - | [5] |  |  |  | 1.68-3.36 | 2.3/1.36 | [6] |
|  | CTX | 38-830 | 69/33 | [25] |  |  |  | ND-4.26 | 0.37/0 | [28] |
|  |  |  |  |  |  |  |  | 7.2-14.66 | 10.8/10.66 | [6] |
|  | AMP | 21-68.7 | 31.5/- | [32] |  |  |  |  |  |  |
|  | AMX | 5.73-14.8 | 9.77/9.08 | [23] | 26.98-91.35 | 51.87/49.61 | [8] |  |  |  |
|  |  | 4.6-710 | 52/23 | [25] | 3.18-34.73 | 13.81/11.12 | [8] |  |  |  |
|  | LIN | ND-1.2 | 0.37/0.42 | [13] | 3.21-11.72 | 6.17/- | [12] | ND-17.46 | 4.7/2.48 | [28] |
|  |  | ND-0.31 | 0.09/- | [15] | 0.3-9.26 | - | [18] | 0.1-138.48 | 19.13/1.45 | [6] |
|  |  | 1.26-58.8 | 8.47/- | [20] | 4.02-9.85 | 7.3/- | [30] |  |  |  |
|  |  | ND-76.48 | 8.04/2.75 | [22] | 0.71-1.37 | 1.1/1.13 | [34] |  |  |  |
|  |  | 0.3-21.13 | 4.77/4.19 | [23] | ND-1.51 | 0.66/0.8 | [34] |  |  |  |
|  |  | 11.3-29.4 | - | [26] |  |  |  |  |  |  |
|  |  | 11.66-80.18 | 32.02/- | [30] |  |  |  |  |  |  |
|  |  | ND-7.74 | 4.39/- | [27] |  |  |  |  |  |  |
|  | CLI | ND-1.29 | 0.24/- | [15] | 3.01-2,406.09 | - | [18] | ND-7.22 | 2.28/0 | [28] |
|  |  | 222.3-63.7 | 37.9/- | [20] | 9.88-53.9 | 22.23/- | [30] | 0.2-90.04 | 12.2/2.33 | [6] |
|  |  | ND-12.9 | 2.59/1.18 | [22] |  |  |  |  |  |  |
|  |  | 5.84-58.44 | 17.95/- | [30] |  |  |  |  |  |  |
|  |  | ND-5.53 | 3.78/- | [27] |  |  |  |  |  |  |
| Antiviral drugs | RTV | - |  |  | 2.0-10.0 | 4.2/- | [36] |  |  |  |
|  | RBV |  |  |  | 0-6.06 | 0.62/0 | [14] |  |  |  |
|  | LPV |  |  |  | ND-14.5 | 4.7/- | [36] |  |  |  |
|  | OTV |  |  |  | 0-4.10 | 0.38/0.03 | [14] |  |  |  |
| NSAIDs | IBF | 2.0-77 | -/22 | [1] | ND-164.9 | 30.1/- | [37] |  |  |  |
|  |  | ND-195 | 61/- | [31] |  |  |  |  |  |  |
|  |  | ND-19.8 | - | [38] |  |  |  |  |  |  |
|  |  | ND-19.97 | 0.51/ND | [13] |  |  |  |  |  |  |
|  |  | ND-13.02 | 2.04/0 | [39] |  |  |  |  |  |  |
|  |  | 49.5-259 | 107/- | [20] |  |  |  |  |  |  |
|  |  | 2.4-320 | 69/26 | [25] |  |  |  |  |  |  |
|  | DFC | ND-26 | -/4.4 | [1] | ND | - | [37] |  |  |  |
|  |  | ND-64 | 20/- | [31] |  |  |  |  |  |  |
|  |  | 2.1-230.4 | - | [38] |  |  |  |  |  |  |
|  |  | ND-4.2 | 0.08/ND | [13] |  |  |  |  |  |  |
|  |  | ND-11.1 | 6.71/- | [15] |  |  |  |  |  |  |
|  |  | ND-22.67 | 4.6/0.39 | [39] |  |  |  |  |  |  |
|  |  | 12.7-69.9 | 38.4/- | [20] |  |  |  |  |  |  |
|  |  | ND-506.92 | 22.65/5.73 | [22] |  |  |  |  |  |  |
|  |  | ND-715 | 127/- | [32] |  |  |  |  |  |  |
|  |  | ND-32 | 2.9/1.9 | [25] |  |  |  |  |  |  |
|  | KPF | ND-0.62 | 0.02/ND | [13] |  |  |  |  |  |  |
|  |  | ND | - | [40] |  |  |  |  |  |  |
|  |  | ND-11.3 | 4.67/- | [15] |  |  |  |  |  |  |
|  |  | ND-19.45 | 0.34/0 | [39] |  |  |  |  |  |  |
|  |  | 7.43-66.2 | 36/- | [20] |  |  |  |  |  |  |
|  | ATP | ND-273.47 | 10.66/4.5 | [13] | ND-211.8 | 11.83/- | [37] |  |  |  |
|  |  | ND-3.88 | 0.56/- | [15] |  |  |  |  |  |  |
|  |  | 7.1-71.7 | 10.14/5 | [4] |  |  |  |  |  |  |
|  |  | ND-89.49 | 1132/- | [22] |  |  |  |  |  |  |
|  |  | 2-7,024 | 42.7/- | [31] |  |  |  |  |  |  |
|  |  | 12.4-88.4 | - | [20] |  |  |  |  |  |  |
|  | NPX | ND-3.9 | -/<LOQ | [40] |  | - |  |  |  |  |
|  |  | ND-22 | 0.34/0 | [1] | ND | 11.83/- | [37] |  |  |  |
|  |  | ND-1.59 | - | [39] |  |  |  |  |  |  |
|  | IM | ND | 5.87/- | [38] |  |  |  |  |  |  |
|  |  | 1.09-20.4 | <LOQ | [20] |  |  |  |  |  |  |
|  |  | ND-2.7 | 219/- | [25] |  |  |  |  |  |  |
| Corticosteroids | DXM | 48.3-686 | 45.2/- | [32] |  |  |  |  |  |  |
|  | PN | ND-276 | -/22 | [32] |  |  |  |  |  |  |

**Table S2** Occurrence of anti-COVID-19 drugs in surface freshwater samples collected from the PRB, China.

| **Group** | **PiE** | **Before the pandemic** | | | **During the pandemic** | | | **After the pandemic** | | |
| --- | --- | --- | --- | --- | --- | --- | --- | --- | --- | --- |
|  |  | **Range** (ng/L) | **Mean/**  **Median** (ng/L) | **Reference** | **Range** (ng/L) | **Mean/**  **Median** (ng/L) | **Reference** | **Range** (ng/L) | **Mean/**  **Median** (ng/L) | **Reference** |
| Antibacterials | ERY | ND-577 | 137/93.1 | [41] | 5.05-22.53 | 6.95/- | [42] | 1.96-4.89 | 2.29/2.18 | [43] |
|  |  | ND-15.05 | 1.98/- | [44] | ND-17.99 | - | [45] |  |  |  |
|  |  |  |  |  | 1.98-2.52 | 2.21/2.19 | [43] |  |  |  |
|  |  |  |  |  | 0.93-2.01 | 1.36/- | [46] |  |  |  |
|  | ROX | 4.03-26.7 | 13.1/12.5 | [41] | ND-8.46 | 2.71/1.52 | [43] | ND-6.34 | 2.53/2.34 | [43] |
|  |  | ND-1.53 | 0.27/- | [44] | 0.52-0.94 | 0.66/- | [46] |  |  |  |
|  | CLR | ND-19.4 | 1.74/ND | [41] |  |  |  |  |  |  |
|  | AZM |  |  |  | ND-877.52 | 3.66/- | [42] | 1.09-4.07 | 2.74/2.46 | [43] |
|  |  |  |  |  | 0.35-4.25 | 1.77/1.84 | [43] |  |  |  |
|  |  |  |  |  | 0.27-1.21 | 0.53/- | [46] |  |  |  |
|  | CPFX | 6.31-49.9 | 14.4/12.2 | [41] | 0.7-33.39 | 2.13/- | [42] | ND-22.02 | 5.38/3.25 | [43] |
|  |  | ND | ND/- | [44] | ND-36.55 | - | [45] |  |  |  |
|  |  |  |  |  | ND-21.98 | 5.85/2.93 | [43] |  |  |  |
|  |  |  |  |  | ND | ND/- | [46] |  |  |  |
|  | OFX | 2.41-60.3 | 13.3/9.89 | [41] | ND- 246.96 | 169.48/149.58 | [47] | ND-133.75 | 17.02/5.37 | [43] |
|  |  | 0.77-7.3 | 3.4/- | [44] | ND-95.26 | 0.69/- | [42] |  |  |  |
|  |  |  |  |  | ND-20.89 | - | [45] |  |  |  |
|  |  |  |  |  | ND-172.15 | 13.64/5.95 | [43] |  |  |  |
|  |  |  |  |  | ND | ND/- | [46] |  |  |  |
|  | NOR | 9.23-27.2 | 13.7/12.1 | [41] | 0.7-20.54 | 1.82/- | [42] | ND-76.24 | 23.9/22.1 | [43] |
|  |  | ND | ND/- | [44] | 22.21-242.07 | - | [45] |  |  |  |
|  |  |  |  |  | ND-46.63 | 13.19/5.82 | [43] |  |  |  |
|  |  |  |  |  | ND | ND/- | [46] |  |  |  |
|  | ENR | ND | ND/- | [44] | 0.19-3.77 | 0.53/- | [42] | 0.89-3.80 | 1.43/1.22 | [43] |
|  |  |  |  |  | ND | ND/- | [45] |  |  |  |
|  |  |  |  |  | 0.63-2.69 | 1.42/1.37 | [43] |  |  |  |
|  |  |  |  |  | ND | ND/- | [46] |  |  |  |
|  | MOX |  |  |  | 0.18-5.5 | 1.31/- | [42] |  |  |  |
| Antiviral drugs | RTV |  |  |  | 1.04-9.47 | 3.16/- | [42] |  |  |  |
|  | LPV |  |  |  | 0.59-21.54 | 1.5/- | [42] |  |  |  |
| NSAIDs | IBF | ND-542 | 127/58.2 | [48] |  |  |  |  |  |  |
|  | DFC | ND-645 | 105/25.2 | [48] |  |  |  |  |  |  |
|  |  | ND-7.71 | 0.824/ND | [49] |  |  |  |  |  |  |
|  | KPF | ND-0.694 | 0.022/ND | [49] |  |  |  |  |  |  |
|  | NPX | ND-4.28 | 1.11/0.63 | [48] |  |  |  |  |  |  |
|  |  | ND-0.954 | 0.119/ND | [49] |  |  |  |  |  |  |
|  | IM | ND-69.6 | 11.6/2.61 | [48] |  |  |  |  |  |  |
|  |  | ND-4.71 | 0.228/ND | [49] |  |  |  |  |  |  |
| Corticosteroids | DXM | <0.3-3.5 | 0.61/0.44 | [50] | ND-0.66 | 0.25/- | [42] |  |  |  |
|  | PN | <0.2-2.3 | 0.44/0.24 | [50] | ND-16.15 | 3.01/- | [42] |  |  |  |
|  | PNL | <0.39-1.8 | 0.65/0.57 | [50] | 0.04-5.16 | 1.75/- | [42] |  |  |  |
|  | HYD |  |  | [50] | ND-9.64 | 2.9/- | [42] |  |  |  |
|  | MP | 0.44-3.8 | 1/0.6 | [50] | ND-6.4 | 1.07/- | [42] |  |  |  |
|  | BUD | 0.4-10 | 2.7/1.9 | [50] |  |  |  |  |  |  |

**Table S3** Occurrence of anti-COVID-19 drugs in surface freshwater samples collected from the YwRB, China.

| **Group** | **PiE** | **Before the pandemic** | | | **During the pandemic** | | | **After the pandemic** | | |
| --- | --- | --- | --- | --- | --- | --- | --- | --- | --- | --- |
|  |  | **Range** (ng/L) | **Mean/**  **Median** (ng/L) | **Reference** | **Range** (ng/L) | **Mean/**  **Median** (ng/L) | **Reference** | **Range** (ng/L) | **Mean/**  **Median** (ng/L) | **Reference** |
| Antibacterials | ERY | 23.3-276.6 | 83.99/53.07 | [51] | ND-7.14 | 2.29/- | [52] | ND−4.65 | 0.362/- | [53] |
|  |  | 7.33-36.8 | 18.54/- | [54] | ND-0.51 | 0.01/- | [55] |  |  |  |
|  |  | ND-38.41 | 13.67/9.5 | [56] | 1.04-17 | 9.24/7.63 | [57] |  |  |  |
|  |  | 1.59-4.02 | -/2.59 | [58] | ND-3.61 | 0.368/- | [53] |  |  |  |
|  |  | 0.3-16.6 | 4.16/2.82 | [59] |  |  |  |  |  |  |
|  | ROX | 1.57-59.49 | 25.52/23.98 | [51] | 0.12-67.64 | 9.626/3.781 | [60] | ND-30.2 | 3.73/- | [53] |
|  |  | 6.53-131.49 | 35.42/- | [54] | ND-0.73 | 0.01/- | [55] |  |  |  |
|  |  | 2.11-126 | 8.77/2.56 | [61] | ND-1.62 | 1.62/1.62 | [57] |  |  |  |
|  |  | ND-39.46 | 20.61/17.8 | [56] | ND-43 | 5.79/- | [53] |  |  |  |
|  |  | <LOQ-1.34 | -/<LOQ | [58] |  |  |  |  |  |  |
|  |  | 0.12-48.7 | 7.1/3.71 | [59] |  |  |  |  |  |  |
|  | CLR | ND-10.10 | 9.11/9.11 | [51] | ND - 13.6 | 1.377/0.50 | [60] |  |  |  |
|  |  | <1.58-8.26 | 0.44/- | [54] | ND | ND/- | [52] |  |  |  |
|  |  | 0.06-7.75 | 1.43/1.03 | [59] | ND-8.85 | 0.27/- | [55] |  |  |  |
|  | AZM | 4.18-68.86 | 12.18/- | [54] | ND-56.72 | 2.07/0.32 | [60] |  |  |  |
|  |  | ND-7.95 | 5.1/5.68 | [56] | ND-17.66 | 0.81/- | [55] |  |  |  |
|  |  | 4.51-8.32 | -/6.19 | [58] | 171.34-193.82 | 173.81/172.32 | [57] |  |  |  |
|  | CPFX | ND-7.32 | 7..32/7.32 | [51] | ND-0.84 | 0.03/- | [55] | ND-36.4 | 15.2/- | [53] |
|  |  | 9.9-28.58 | 14.75/- | [54] | 1.92-24.5 | 16/21.58 | [57] |  |  |  |
|  |  | ND-10.1 | 2.96/3.46 | [61] | ND | ND/- | [53] |  |  |  |
|  |  | ND-42.74 | 26.6/27.02 | [56] |  |  |  |  |  |  |
|  |  | 2.19-51.8 | -/20.7 | [58] |  |  |  |  |  |  |
|  | OFX | <1.77-154.79 | 7.68/- | [54] | ND-0.2 | 0.01 | [55] | ND-306 | 19.8/- | [53] |
|  |  | ND-511 | 25/3.07 | [61] | 0.54-1,220.86 | 180.21/0.54 | [57] |  |  |  |
|  |  | 3.87-254.74 | 102.6/104.97 | [56] | ND−80.6 | 13.2/- | [53] |  |  |  |
|  |  | <LOQ-144 | -/1.92 | [58] |  |  |  |  |  |  |
|  |  | ND-8.33 | 0.94/0.24 | [59] |  |  |  |  |  |  |
|  | NOR | ND-39.21 | 18.21/8.14 | [51] | 6.14 - 382.3 | 43.47/23.84 | [60] | ND-730 | 41.6/- | [53] |
|  |  | 8.66-53.28 | 14.89/- | [54] | ND-2.13 | 0.41/- | [52] |  |  |  |
|  |  | ND-1953 | 87/4.74 | [61] | ND | ND/- | [55] |  |  |  |
|  |  | 7.45-173.44 | 54.68/36.2 | [56] | 1.9-520.86 | 56.98/6.29 | [57] |  |  |  |
|  |  | 7.94-71.3 | -/26.6 | [58] | ND-65.6 | 4.2/- | [53] |  |  |  |
|  | ENR | 6.39-117.9 | 15.42/- | [54] | 8.83 - 206.7 | 27.35/18.63 | [60] | ND | ND/- | [53] |
|  |  | 7.9-18.81 | 13.5 | [56] | ND | ND/- | [52] |  |  |  |
|  |  | <LOQ-15.2 | -/<LOQ | [58] | ND-0.11 | 0.01/- | [55] |  |  |  |
|  |  |  |  |  | 2.46-27.06 | 4.63/2.77 | [57] |  |  |  |
|  |  |  |  |  | ND−0.99 | 0.098/- | [53] |  |  |  |
|  | MOX |  |  |  | ND-0.25 | 0.01/- | [55] |  |  |  |
|  | AMP | ND-15.1 | 1.32/ND | [61] |  |  |  |  |  |  |
|  | AMX | 34.6-786.4 | 173.8/107.66 | [56] |  |  |  |  |  |  |
|  |  | 12.03-1,334 | -/371 | [58] |  |  |  |  |  |  |
|  | LIN | 3.63-125.33 | 23.81/9.1 | [51] | ND-159.38 | 8.02/- | [55] |  |  |  |
|  | CLI |  |  |  | ND-100.45 | 7.38/- | [55] |  |  |  |
| NSAIDs | IBF |  |  |  | 0.78 - 146.7 | 29.51/15.81 | [60] |  |  |  |
|  | ATP |  |  |  | 3.15 - 341.6 | 47.15/26.03 | [60] |  |  |  |
|  | NPX |  |  |  | ND-17.52 | 3.915/2.571 | [60] |  |  |  |
|  | IM |  |  |  | ND - 76.59 | 15.87/12.08 | [60] |  |  |  |

**References**

1. Xie Z, Lu G, Yan Z, Liu J, Wang P, Wang Y. Bioaccumulation and trophic transfer of pharmaceuticals in food webs from a large freshwater lake. Environ Pollut*.* 2017;222:356–66.
2. Chen Y, Tan Y, Wang Y, Ma Y, Li P, Du Z, Yang L, Wu L, Cui S, Ding Y, Qi X, Zhang Z. Estimating Sources, Fluxes, and Ecological Risks of Antibiotics in the Wuhan Section of the Yangtze River, China: A Year-Long Investigation. Environ Toxicol Chem. 2023;42(3):605–19.
3. Ding F, Li Y, He T, Wang Y, Li Y, Huang Y, Yin G, Yang J, Wu S, Liu Y, Liu M. Land use and spatial contiguity are key driven factors of antibiotic multimedia patterns in the megacity river network. Sci Total Environ. 2024;947:174727.
4. Hu XL, Bao YF, Hu JJ, Liu YY, Yin DQ. Occurrence of 25 pharmaceuticals in Taihu Lake and their removal from two urban drinking water treatment plants and a constructed wetland. Environ. Sci Pollut Res Int. 2017;24(17):14889–902.
5. Chen X, Lei L, Liu S, Han J, Li R, Men J, Li L, Wei L, Sheng Y, Yang L, Zhou B, Zhu L. Occurrence and risk assessment of pharmaceuticals and personal care products (PPCPs) against COVID-19 in lakes and WWTP-river-estuary system in Wuhan, China. Sci Total Environ. 2021;792:148352.
6. Zhao J, Guo C, Yang Q, Liu W, Zhang H, Luo Y, Zhang Y, Wang L, Chen C, Xu J. Comprehensive monitoring and prioritizing for contaminants of emerging concern in the Upper Yangtze River, China: An integrated approach. J Hazard Mater. 2024;480:135835.
7. Tong L, Qin L, Guan C, Wilson ME, Li X, Cheng D, Ma J, Liu H, Gong F. Antibiotic resistance gene profiling in response to antibiotic usage and environmental factors in the surface water and groundwater of Honghu Lake, China. Environ Sci Pollut Res Int. 2020;27(25):31995–2005.
8. Li Y, Wang J, Lin C, Lian M, Wang A, He M, Liu X, Ouyang W. Riverine antibiotic occurrence and potential ecological risks in a low-urbanized and rural basin of the middle Yangtze River: Socioeconomic, land use, and seasonal effects. Environ Res. 2023;228:115827.
9. Liang X, Guan F, Chen B, Luo P, Guo C, Wu G, Ye Y, Zhou Q, Fang H. Spatial and seasonal variations of antibiotic resistance genes and antibiotics in the surface waters of Poyang Lake in China. Ecotoxicol Environ Saf. 2020;196:110543.
10. Zho Q, Liu G, Arif M, Shi X, Wang S. Occurrence and risk assessment of antibiotics in the surface water of Chaohu Lake and its tributaries in China. Sci Total Environ. 2022;807:151040.
11. Sun S, Chen Y, Lin Y, An D. Occurrence, spatial distribution, and seasonal variation of emerging trace organic pollutants in source water for Shanghai, China. Sci Total Environ. 2018; 639:1–7.
12. Wu T, Zhang Y, Wang B, Chen C, Cheng Z, Li Y, Wang B, Li J. Antibiotic resistance genes in Chishui River, a tributary of the Yangtze River, China: Occurrence, seasonal variation and its relationships with antibiotics, heavy metals and microbial communities. Sci Total Environ. 2022;846:157472.
13. Liu S, Wang C, Wang P, Chen J, Wang X, Yuan Q. Anthropogenic disturbances on distribution and sources of pharmaceuticals and personal care products throughout the Jinsha River Basin, China. Environ Res. 2021;198:110449.
14. Chen M, Jin X, Liu Y, Guo L, Ma Y, Guo C, Wang F, Xu J. Human activities induce potential aquatic threats of micropollutants in Danjiangkou Reservoir, the largest artificial freshwater lake in Asia. Sci Total Environ. 2022;850:157843.
15. Wang Y, Liu Y, Lu S, Liu X, Meng Y, Zhang G, Zhang Y, Wang W, Guo X. Occurrence and ecological risk of pharmaceutical and personal care products in surface water of the Dongting Lake, China-during rainstorm period. Environ Sci Pollut Res Int. 2019;26(28):28796-807.
16. Jiang L, Zhai W, Wang J, Li G, Zhou Z, Li B, Zhuo H. Antibiotics and antibiotic resistance genes in the water sources of the Wuhan stretch of the Yangtze River: Occurrence, distribution, and ecological risks. Environ Res. 2023;239(Pt 1):117295.
17. Wang G, Zhou S, Han X, Zhang L, Ding S, Li Y, Zhang D, Zarin K. Occurrence, distribution, and source track of antibiotics and antibiotic resistance genes in the main rivers of Chongqing city, Southwest China. J Hazard Mater. 2020;389:122110.
18. Wang N, Wang N, Qi D, Kang G, Wang W, Zhang C, Zhang Z, Zhang Y, Zhang H, Zhang S, Xu J.Comprehensive overview of antibiotic distribution, risk and priority: A study of large-scale drinking water sources from the lower Yangtze River. J Environ Manage. 2023;344:118705.
19. Zhang G, Lu S, Wang Y, Liu X, Liu Y, Xu J, Zhang T, Wang Z, Yang Y. Occurrence of antibiotics and antibiotic resistance genes and their correlations in lower Yangtze River, China. Environ Pollut. 2020;257:113365.
20. Jiang X, Zhu Y, Liu L, Fan X, Bao Y, Deng S, Cui Y, Cagnetta G, Huang J, Yu G. Occurrence and variations of pharmaceuticals and personal-care products in rural water bodies: A case study of the Taige Canal (2018-2019). Sci Total Environ. 2021;762:143138.
21. Mu Y, Tang B, Cheng X, Fu Y, Huang W, Wang J, Ming D, Xing L, Zhang J. Source apportionment and predictable driving factors contribute to antibiotics profiles in Changshou Lake of the Three Gorges Reservoir area, China.  J Hazard Mater. 2024;466:133522.
22. An W, Duan L, Zhang Y, Zhou Y, Wang B, Yu G. Pollution characterization of pharmaceutically active compounds (PhACs) in the northwest of Tai Lake Basin, China: Occurrence, temporal changes, riverine flux and risk assessment. J Hazard Mater. 2022;422:126889.
23. Zhu F, Wang S, Liu Y, Wu M, Wang H, Xu G. Antibiotics in the surface water of Shanghai, China: screening, distribution, and indicator selecting. Environ Sci Pollut Res Int. 2021;28(8):9836–48.
24. Li Y, Tong L, Zhang J, Liu H, Li M, Wen Z. Distribution and risk assessment of antibiotics under water level fluctuation in the riparian zone of the Hanjiang River. Ecotoxicol Environ Saf. 2023;256:114833.
25. Lin H, Chen L, Li H, Luo Z, Lu J, Yang Z. Pharmaceutically active compounds in the Xiangjiang River, China: Distribution pattern, source apportionment, and risk assessment. Sci Total Environ*.* 2018;636:975–84.
26. Guo X, Xiao JL, Zhang A, Yan Z, Chen S, Wang N. Antibiotic contamination in a typical water-rich city in southeast China: a concern for drinking water resource safety. J Environ Sci Health B. 2020;55(3):193–209.
27. Liu Y, Feng M, Wang B, Zhao X, Guo R, Bu Y, Zhang S, Chen J. Distribution and potential risk assessment of antibiotic pollution in the main drinking water sources of Nanjing, China. Environ Sci Pollut Res Int. 2020;27(17):21429-41.
28. Luo Y, Jin X, Zhao J, Xie H, Guo X, Huang D, Giesy JP, Xu J.  Ecological implications and drivers of emerging contaminants in Dongting Lake of Yangtze River Basin, China: A multi-substance risk analysis. J Hazard Mater. 2024;472:134519.
29. Huang J, Ding J, Jiang H, Wang Z, Zheng L, Song X, Zou H. Pharmaceuticals and Personal Care Products across Different Water Bodies in Taihu Lake Basin, China: Occurrence, Source, and Flux. Int J Environ Res Public Health. 2022;19(17):11135.
30. Liu Y, Chen Y, Feng M, Chen J, Shen W, Zhang S. Occurrence of antibiotics and antibiotic resistance genes and their correlations in river-type drinking water source, China. Environ. Sci Pollut Res Int. 2021;28(31):42339–52.
31. Zhou H, Ying T, Wang X, Liu J. Occurrence and preliminarily environmental risk assessment of selected pharmaceuticals in the urban rivers, China. Sci Rep. 2016;6:34928.
32. Tang J, Sun J, Wang W, Yang L, Xu Y. Pharmaceuticals in two watersheds in Eastern China and their ecological risks. Environ Pollut. 2021;277:116773.
33. Yan M, Xu C, Huang Y, Nie H, Wang J. Tetracyclines, sulfonamides and quinolones and their corresponding resistance genes in the Three Gorges Reservoir, China. Sci Total Environ. 2018;631-632:840–8.
34. Zeng Y, Duan L, Xu T, Hou P, Zhang X, Wang L, et al. Occurrence and risk assessment of antibiotics in urban river–wetland–lake systems in southwest China. Water. 2024;16(8):1124.
35. Linghu K, Wu Q, Zhang J, Wang Z, Zeng J, Gao S, et al. Occurrence, distribution and ecological risk assessment of antibiotics in Nanming river: Contribution from wastewater treatment plant and implications of urban river syndrome. Process Saf Environ Prot. 2023;169:428-436.
36. Zhang Z, Zhou Y, Han L, Guo X, Wu Z, Fang J, Hou B, Cai Y, Jiang J, Yang Z. Impacts of COVID-19 pandemic on the aquatic environment associated with disinfection byproducts and pharmaceuticals. Sci Total Environ. 2022;811:151409.
37. Yin C, Tan Y, Chen Y, Gao S, Wu M, Zhang Z. Mass load and source apportionment of pharmaceutical and personal care product in the Wuhan section of the Yangtze River, China. Sci Total Environ. 2025;959:178222.
38. Ma R, Wang B, Lu S, Zhang Y, Yin L, Huang J, Deng S, Wang Y, Yu G.  Characterization of pharmaceutically active compounds in Dongting Lake, China: Occurrence, chiral profiling and environmental risk. Sci Total Environ. 2016;557-558:268–275.
39. Cao SS, Duan YP, Tu YJ, Tang Y, Liu J, Zhi WD, Dai C.Pharmaceuticals and personal care products in a drinking water resource of Yangtze River Delta Ecology and Greenery Integration Development Demonstration Zone in China: Occurrence and human health risk assessment. Sci Total Environ. 2020;721:137624.
40. Ma R, Wang B, Yin L, Zhang Y, Deng S, Huang J, Wang Y, Yu G. Characterization of pharmaceutically active compounds in Beijing, China: Occurrence pattern, spatiotemporal distribution and its environmental implication. J Hazard Mater. 2017;323(Pt A):147-55.
41. Wang C, Zhao Y, Liu S, Xiao Q, Liang W, Song Y. Contamination, distribution, and risk assessment of antibiotics in the urban surface water of the Pearl River in Guangzhou, South China. Environ Monit Assess. 2021;193(2):98.
42. Wu H, Bin L, Guo P, Zhao Y, Chen C, Chen Z, Tang B. Ecological risk assessment of the typical anti-epidemic drugs in the Pearl River Delta by tracing their source and residual characteristics. J Hazard Mater. 2024;463:132914.
43. Zhang H, Ouyang W, Lin C, Wang L, Guo Z, Pei J, Zhang S, He M, Liu X. Anthropogenic activities drive the distribution and ecological risk of antibiotics in a highly urbanized river basin. Sci Total Environ. 2024;938:173596.
44. Zhao B, Xu J, Zhang G, Lu S, Liu X, Li L, Li M. Occurrence of antibiotics and antibiotic resistance genes in the Fuxian Lake and antibiotic source analysis based on principal component analysis-multiple linear regression model. Chemosphere. 2021;262:127741.
45. Jiang S, Shi B, Zhu D, Cheng X, Zhou Z, Xie J, Chen Z, Sun L, Zhang Y, Xie Y, Jiang L. Cross-contamination and ecological risk assessment of antibiotics between rivers and surrounding open aquaculture ponds. Environ Pollut. 2024;344:123404.
46. Qadeer A, Rui G, Yaqing L, Ran D, Liu C, Jing D, Anis M, Liu M, Wang S, Jiang X, Zhao X. A mega study of antibiotics contamination in Eastern aquatic ecosystems of China: occurrence, interphase transfer processes, ecotoxicological risks, and source modeling. J Hazard Mater. 2023;458:131980.
47. Zhang Y, Li J, Wu T, Ma K, Cheng Z, Yi Q, Dai Y, Wang B, Chen Y, Wang B, Hu X, Yang A, Yang Q, Zhong X. Characteristics of antibiotic resistance genes and microbial community distribution in Wanfeng Lake, upper Pearl River, China. Environ Sci Pollut Res Int. 2023;30(35):83214-30.
48. Peng FJ, Pan CG, Zhang M, Zhang NS, Windfeld R, Salvito D, Selck H, Van den Brink PJ, Ying GG.Occurrence and ecological risk assessment of emerging organic chemicals in urban rivers: Guangzhou as a case study in China. Sci Total Environ. 2017;589:46–55.
49. Cui Y, Wang Y, Pan C, Li R, Xue R, Guo J, Zhang R. Spatiotemporal distributions, source apportionment and potential risks of 15 pharmaceuticals and personal care products (PPCPs) in Qinzhou Bay, South China. Mar Pollut Bull. 2019;141:104–11.
50. Gong J, Lin C, Xiong X, Chen D, Chen Y, Zhou Y, Wu C, Du Y. Occurrence, distribution, and potential risks of environmental corticosteroids in surface waters from the Pearl River Delta, South China. Environ Pollut. 2019;251:102–9.
51. Wang J, Wei H, Zhou X, Li K, Wu W, Guo M. Occurrence and risk assessment of antibiotics in the Xi'an section of the Weihe River, northwestern China. Mar Pollut Bull. 2019;146:794–800.
52. Su Z, Wang K, Yang F, Zhuang T. Antibiotic pollution of the Yellow River in China and its relationship with dissolved organic matter: Distribution and Source identification. Water Res. 2023;235:119867.
53. Zhang Y, Liu L, Liu Y, Chen L, Wang J, Li Y, Wang K, Wang W. Deciphering the natural and anthropogenic drivers on the fate and risk of antibiotics and antibiotic resistance genes (ARGs) in a typical river-estuary system, China. J Hazard Mater. 2024;480:136006.
54. Wang L, Wang Y, Li H, Zhu Y, Liu R. Occurrence, source apportionment and source-specific risk assessment of antibiotics in a typical tributary of the Yellow River basin. J Environ Manage. 2022;305:114382.
55. Tian B, Zhang M, Zhu C, Yang R, Yin G, Hu S, Chen Y, Zhao N. Contrastive cognition into the occurrence, source identification and risk assessment of antibiotics in various drinking water sources. Environ Pollut. 2025;374:126226.
56. Lu S, Lin C, Lei K, Wang B, Xin M, Gu X, Cao Y, Liu X, Ouyang W, He M. Occurrence, spatiotemporal variation, and ecological risk of antibiotics in the water of the semi-enclosed urbanized Jiaozhou Bay in eastern China. Water Res. 2020;184:116187.
57. Zhang F, Mao X, Song X, Yu H, Yan J, Kong D, Liu Y, Yao N, Yang S, Xie S, Ji H, Zhou H. Ecological Risks of Antibiotics in Urban Wetlands on the Qinghai-Tibet Plateau, China. Int J Environ Res Public Health. 2023;20(3):1735.
58. Lu S, Wang J, Wang B, Xin M, Lin C, Gu X, Lian M, Li Y. Comprehensive profiling of the distribution, risks and priority of pharmaceuticals and personal care products: A large-scale study from rivers to coastal seas. Water Res. 2023;230:119591.
59. Kuang Y, Guo X, Hu J, Li S, Zhang R, Gao Q, Yang X, Chen Q, Sun W. Occurrence and risks of antibiotics in an urban river in northeastern Tibetan Plateau. Sci Rep. 2020;10(1):20054.
60. Yu X, Yu F, Li Z, Zhan J. Occurrence, distribution, and ecological risk assessment of pharmaceuticals and personal care products in the surface water of the middle and lower reaches of the Yellow River (Henan section). J Hazard Mater. 2023;443:130369.
61. Feng J, Liu Q, Ru X, Xi N, Sun J. Occurrence and distribution of priority pharmaceuticals in the Yellow River and the Huai River in Henan, China. Environ. Sci Pollut Res Int. 2020;27(14):16816–26.
