# Supplementary material for: Occurrence of antibacterials, antivirals, and anti-inflammatory pharmaceuticals for COVID-19 treatment as emerging contaminants in the Chinese freshwater environment before, during and after the pandemic: the need for dynamic eco-pharmacovigilance
Source: Environ Health Prev Med. 2026 Jul 3;31:44. doi: 10.1265/ehpm.25-00395 (PMC13366171; doi:10.1265/ehpm.25-00395)
Supplement: Supplementary file 3 — Additional file 3: Occurrence analysis of four classes of anti-COVID-19 drugs in the HaRB, HuRB, LRB and SRB. [file ehpm-31-044-s003.docx]

**Occurrence of antibacterials, antivirals, and anti-inflammatory pharmaceuticals for COVID-19 treatment as emerging contaminants in the Chinese freshwater environment before, during and after the pandemic: The need for dynamic eco-pharmacovigilance**

Sijia Ma, Hongxia Chen and Jun Wang *

*Correspondence: wangjun@wust.edu.cn

Institute of Pharmaceutical Innovation, Hubei Province Key Laboratory of Occupational Hazard Identification and Control, School of Medicine, Wuhan University of Science and Technology, Wuhan 430065, China

**Spatial-temporal variations in the occurrence of anti-COVID-19 drugs in the HaRB**

With a drainage area of 318,000 km^2^ covering the Beijing-Tianjin-Hebei region (the largest urban cluster in Northern China), the HaRB is characterized by high population density and rapid economic development, making it one of the most contaminated river basins in China [1,2]. The HaRB encompasses the mainstreams and tributaries of the Haihe, Beiyun, Wenyu, and Yongdingxin Rivers, and receives wastewater from approximately 70% of the population (about 28 million people) and 90% of the drainage systems on the North China Plain [2]. In recent decades, the Chinese government has paid considerable attention to water pollution control in the HaRB [1,2].

In the HaRB, post-pandemic data on the occurrence of anti-COVID-19 drugs as emerging contaminants in freshwater remain lacked. As shown in Table S4, the comparison of occurrence data before and during the COVID-19 outbreak indicated that, despite relatively scarce data during the pandemic reported in only three studies [3,4,5], the HaRB exhibited a notable improvement in surface water quality with respect to anti-COVID-19 drugs over time, in contrast to other river systems in China. Before the pandemic, multiple drugs that were later used for COVID-19 treatment, including the antibacterials ERY, AZM, and OFX, as well as a NSAID ATP, were detected at residual concentrations exceeding 1,000 ng/L in the HaRB. During the pandemic, however, the highest concentration among these drugs was only 201.68 ng/L (ERY). Accordingly, the cumulative maximum concentration of the included anti-COVID-19 drugs in surface water across the entire HaRB was higher before the COVID-19 outbreak than during the pandemic lockdown period (Figure S1). Especially in 2015, the cumulative maximum concentration of drugs in the Beiyun River in the HaRB exceeded 5,200 ng/L [6]. In the Beijing area, cumulative maximum concentrations of selected anti-COVID-19 drugs in surface water decreased from 2,559.8 ng/L in 2014 to 486.73 ng/L in 2020, then slightly increased to 560.01 ng/L in 2021 [4,7]. Similarly, cumulative maximum concentrations of studied pharmaceuticals in the Beiyun river, an important main channel of the HaRB, decreased significantly from pre-COVID-19 levels of 2,990.6-5,255.4 ng/L to 625.6 ng/L in 2021 during the pandemic [3,6,8,9].

**Table S4** Occurrence of anti-COVID-19 drugs in surface freshwater samples collected from the HaRB.

| **Group** | **PiE** | **Before the pandemic** | | | **During the pandemic** | | |
| --- | --- | --- | --- | --- | --- | --- | --- |
|  |  | **Range** (ng/L) | **Mean/**  **Median**  (ng/L) | **Reference** | **Range** (ng/L) | **Mean/**  **Median**  (ng/L) | **Reference** |
| Antibacterials | ERY | ND-372 | 20.8/4.6 | [7] | <LOQ-201.68 | 18.28/1.22 | [4] |
|  |  | ND-1,320 | - | [6] | 0.25-54 | 9.99/8.03 | [5] |
|  |  | 38.4-227 | 92.8/- | [2] | ND-29.6 | 4.42/- | [3] |
|  |  | ND-364 | -/64 | [8] | ND-16.1 | 6.05/- | [10] |
|  |  | ND-728 | 95.3/- | [10] |  |  |  |
|  |  | ND-107.26 | 37.89/- | [11] |  |  |  |
|  |  | ND-27.6 | 11.6/- | [12] |  |  |  |
|  |  | ND-84 | - | [13] |  |  |  |
|  |  | 0-642.3 | 24.5/- | [9] |  |  |  |
|  |  | 0.18-273 | 29.7/16.1 | [14] |  |  |  |
|  |  | ND-0.9 | - | [15] |  |  |  |
|  |  | 58.6-299 | - | [16] |  |  |  |
|  | ROX | ND-352 | 26.7/3.83 | [7] | <LOQ-96.83 | 11.4/0.41 | [4] |
|  |  | 43.9-235 | 84.1/- | [2] | <LOQ-8.3 | 1.76/1.07 | [5] |
|  |  | ND-327 | -/37 | [8] | ND-47.8 | 12.7/- | [3] |
|  |  | ND-24.2 | 4.71/- | [10] | ND-5.77 | 2.51/- | [10] |
|  |  | ND-69 | - | [13] |  |  |  |
|  |  | 0.14-526 | 45.4/10.9 | [14] |  |  |  |
|  | CLR | ND-96.9 | - | [6] | <LOQ-55.58 | 6.28/0.22 | [4] |
|  |  | ND-347 | -/33.2 | [8] | ND-49.5 | 11.8/- | [3] |
|  |  | 0.07-78.1 | 11.4/2.26 | [14] | 1.26-12.0 | 3.96/- | [10] |
|  |  | ND-4.95 | - | [15] |  |  |  |
|  | AZM | <LOQ-37.3 | 9/- | [2] | ND-166 | 6.96/0.33 | [5] |
|  |  | ND-215.06 | 45.27/- | [10] | ND-83.2 | 20.9/- | [3] |
|  |  | ND-16.7 | 9.55/- | [11] | ND-84.1 | 24.2/- | [10] |
|  |  | 0.45-1,060 | 46/1 | [12] |  |  |  |
|  |  | ND-2.47 | - | [15] |  |  |  |
|  | CPFX | ND-414 | 9.87/2.23 | [7] | <LOQ-8.21 | 0.63/<LOQ | [4] |
|  |  | 20.9-84.9 | 37.4/- | [2] | 0.35-14.4 | 1.97/1.29 | [5] |
|  |  | ND-11.4 | 0.89/- | [10] | ND-13 | 2.14/- | [3] |
|  |  | ND-36.9 | - | [13] | ND-8.48 | 2.39/- | [10] |
|  |  | ND-148 | 124/- | [17] |  |  |  |
|  |  | 0-232 | 8.8/- | [9] |  |  |  |
|  |  | 1.25-29.8 | 6.58/4.96 | [14] |  |  |  |
|  |  | ND-15.77 | - | [15] |  |  |  |
|  | OFX | 0.34-990 | 93.5/11.1 | [7] | <LOQ-106.77 | 6.44/<LOQ | [4] |
|  |  | 36.6-374 | 101/- | [2] | <LOQ-44.4 | 3.3/1.44 | [5] |
|  |  | ND-41.8 | 9.21/- | [10] | ND-114 | 18.7/- | [3] |
|  |  | ND-215.06 | 45.27/- | [11] | ND-17.0 | 3.84/- | [10] |
|  |  | 2.1-26.4 | 13.8/- | [12] |  |  |  |
|  |  | 80.9-1,270 | - | [13] |  |  |  |
|  |  | 8.86-428 | 89.2/- | [17] |  |  |  |
|  |  | 0-623.6 | 25.1/- | [9] |  |  |  |
|  |  | 0.33-1,000 | 79.8/1.48 | [14] |  |  |  |
|  |  | ND-76.25 | - | [15] |  |  |  |
|  | NOR | 0.87-403 | 27.6/7.65 | [7] | 1.42-16.6 | 5.76/5.1 | [5] |
|  |  | 27.5-188 | 54.5/- | [2] | ND-193 | 40/- | [3] |
|  |  | ND-105 | 23.1/- | [10] | ND-31.2 | 12.9/- | [10] |
|  |  | ND-113 | - | [13] |  |  |  |
|  |  | ND | ND/- | [17] |  |  |  |
|  |  | 0-466.3 | 20.2/- | [9] |  |  |  |
|  |  | 2.26-123 | 25.4/15.7 | [14] |  |  |  |
|  |  | ND-30.66 | - | [15] |  |  |  |
|  | ENR | ND-28.8 | 0.31/ND | [7] | 0.23-31.93 | 4.97/3.8 | [4] |
|  |  | 51.6-184 | 78.4/- | [2] | ND-18 | 0.97/- | [3] |
|  |  | ND-64.71 | 27.94/- | [10] | ND-3.68 | 0.93/- | [10] |
|  |  | ND | - | [13] |  |  |  |
|  |  | ND-111 | 34.3/- | [17] |  |  |  |
|  |  | <LOQ-182 | 5.29/0.79 | [14] |  |  |  |
|  |  | ND-2.82 | - | [15] |  |  |  |
|  | CTX | 4.74-12.4 | - | [16] |  |  |  |
|  | AMP | ND-2.35 | - | [15] | ND | ND/- | [3] |
|  |  | <0.43-18 | - | [16] | ND | ND/- | [10] |
|  | AMX | ND-0.51 | - | [15] |  |  |  |
|  | LIN | 0.13-152 | -/11.3 | [8] | ND-45.8 | 5.74/- | [3] |
|  |  | ND-407.12 | 107.13/- | [11] | ND-2.91 | 0.42/- | [10] |
|  |  | ND-40.5 | 23.5/- | [12] |  |  |  |
|  | CLI | 0.16-65.8 | -/15.6 | [8] |  |  |  |
| Antiviral drugs | RTV |  |  |  | ND-18 | 1.64/- | [3] |
|  |  |  |  |  | 20.5-81.7 | 46.2/- | [10] |
|  | LPV |  |  |  | ND-2.46 | 1.04/- | [10] |
|  | RBV |  |  |  | <LOQ-64.07 | 11.84/8.31 | [4] |
|  | OTV |  |  |  | <LOQ-69.92 | 2.44/<LOQ | [4] |
| NSAIDs | DFC | 1.8-121.6 |  | [6] | ND-13 | 2.14/- | [3] |
|  |  | 0.69-128 |  | [8] | 16.9-43.8 | 28.9/- | [10] |
|  |  | 0-986.5 | - | [9] |  |  |  |
|  | KPF | ND-65 | -/16.7 | [6] |  |  |  |
|  |  | 3.52-219 | 31.9/- | [8] |  |  |  |
|  |  | 0-17.3 | - | [9] |  |  |  |
|  | ATP | ND-3,577 | -/51.9 | [9] |  |  |  |
|  |  | 6.2-2,110 | 4.5/- | [8] |  |  |  |
|  |  | ND-71.95 | - | [11] |  |  |  |
|  |  | 28.6-507 | -/156 | [12] |  |  |  |
|  | IM | ND-74.9 | 31.46/- | [6] |  |  |  |
|  |  | 0.48-68.7 | 155/- | [8] |  |  |  |
|  |  | 0-52.6 | - | [9] |  |  |  |


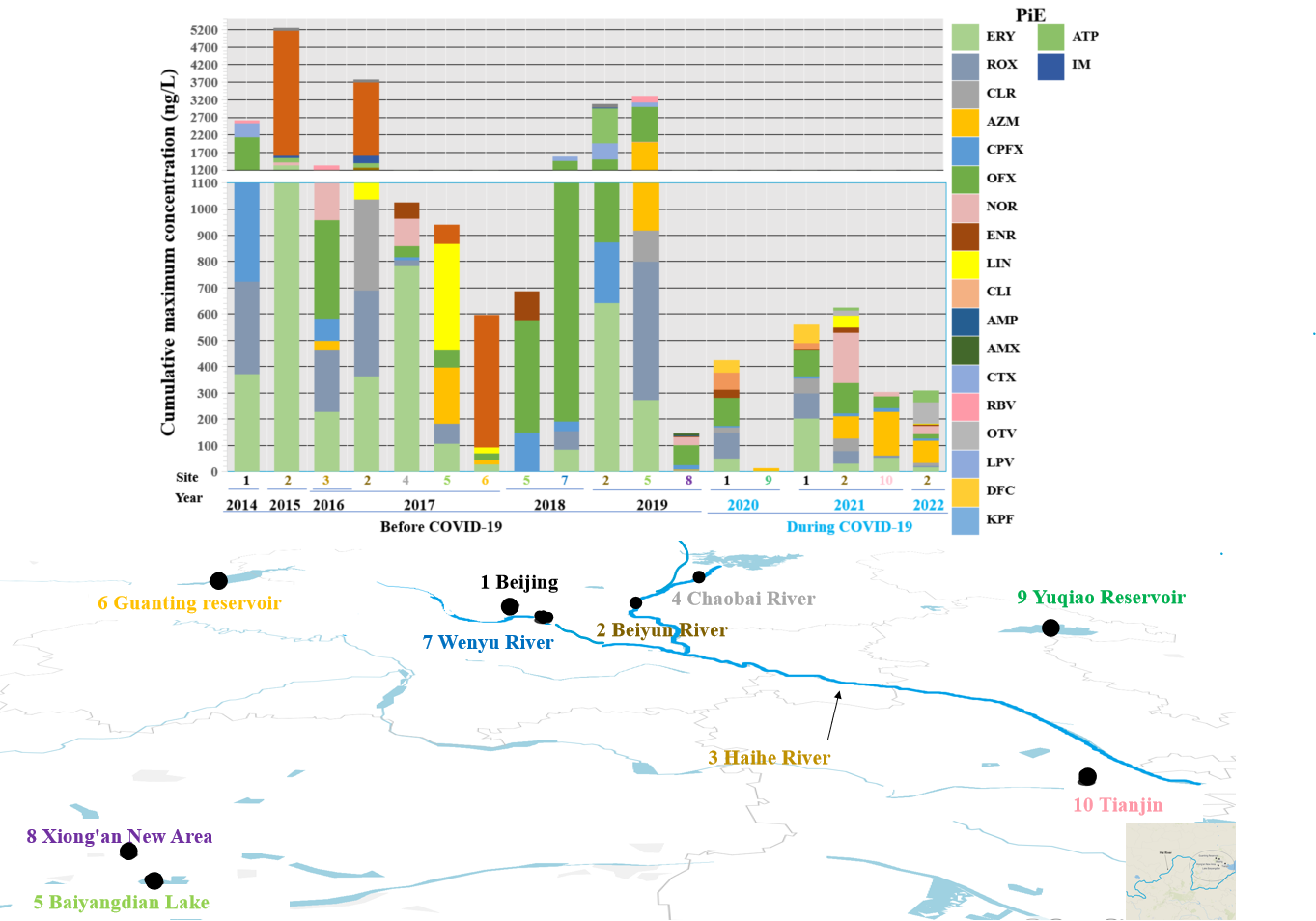


**Fig. S1** Cumulative maximum residue concentrations of anti-COVID-19 drugs collected at representative sampling point in the HaRB.

Such a significant improvement in pharmaceutical pollution may be attributed to China’s strengthened pollution control efforts along the HaRB in recent years. The HaRB has identified as one of the key priority areas in the National 12th and 13th Five-Year Plans for Water Pollution Control implemented by the Chinese government[1,2]. However, more updated occurrence data are still required to better understand the dynamic contamination status of pharmaceuticals in the HaRB’s water systems, which is essential for large-scale control and management of these emerging contaminants.

**Spatial-temporal variations in the occurrence of anti-COVID-19 drugs in the HuRB**

As the geographic boundary between northern and southern China, the HuRB (111.83°-121.42°E, 30.92°-36.60°N) spans approximately 270,000 km^2^ across eastern China, supporting around 190 million people and approximately 120,000 km² of cropland. The population density in the HuRB is more than four times the national average. Since the mid-twentieth century, the HuRB has experienced intense anthropogenic disturbance, with wastewater discharges significantly exacerbating water pollution [19-21].

As shown in Table S5 and Figure S2, the occurrence of a total of 11 antibacterials and 4 NSAIDs in freshwater samples collected in the HuRB was reported by 9 studies[22,23,24-28] . Before the COVID-19 outbreak, the top three PiE in terms of maximum residual concentrations in water samples were ROX (566.79 ng/L), KPF (531.43 ng/L), and CLI (216.38 ng/L). During the pandemic, the residual concentrations of antibacterial PiE in the HuRB significantly decreased compared to pre-COVID-19 levels, with only two compounds (CPFX and ENR) exceeding 50 ng/L. In contrast, NSAID pollution became significantly more severe during the pandemic. Wang et al. [24] collected lake water samples in December 2019 (before the COVID-19 outbreak) and April 2020 (during the pandemic) from the lower reaches of the HuRB. This study [24] found the maximum residual concentrations of four NSAIDs commonly used for COVID-19 treatment, IBF, KPF, NPX, and IM, during the pandemic increased by approximately 6.9-, 3.2-, 31.5-, and 23.7-fold, respectively. In particular, the risk assessment showed that NPX residues in the lake waters during the pandemic posed moderate risks to aquatic organisms, and a potential risk to human infant health [24]. After the pandemic, CPFX was detected in a reservoir at the junction of the Dabie Mountains and the Huaihe River at concentrations up to 1,409 ng/L, followed by LIN (541.2 ng/L) and OFX (326 ng/L) [27]. Figure S2 showed that, across all studied areas of the HuRB, the highest cumulative maximum concentration of the included anti-COVID-19 drugs was detected in Luoma Lake, one of the largest freshwater lakes in the HuRB, reaching its peak of approximately 3,120 ng/L in 2020 [24], which was much higher than in 2019 [22,28]. The second most contaminated freshwater system by the studied PiE was the Nanwan Reservoir, with a cumulative maximum concentration of approximately 2,620 ng/L in 2023 [27].

**Table S5** Occurrence of anti-COVID-19 drugs in surface freshwater samples collected from the HuRB.

| **Group** | **PiE** | **Before the pandemic** | | **During the pandemic** | | **After the pandemic** | |
| --- | --- | --- | --- | --- | --- | --- | --- |
|  |  | **Range**(ng/L) | **Reference** | **Range**(ng/L) | **Reference** | **Range**(ng/L) | **Reference** |
| Antibacterials | ERY | <LOQ | [28] | 6.35–29.5 | [25] | 5.69-6.77 | [26] |
|  |  |  |  | <LOQ-13.92 | [20] |  |  |
|  | ROX | <LOQ-566.78 | [28] | <LOQ-13.92 | [20] | ND-2.89 | [26] |
|  |  | ND-33.9 | [23] | ND-1.16 | [25] | 0-3.79 | [27] |
|  | CLR | <LOQ-66.7 | [28] | 4.47-7.68 | [20] | ND-6.26 | [26] |
|  |  |  |  |  |  | 0-0.7 | [27] |
|  | AZM | <LOQ-5.69 | [28] | ND-7.82 | [25] | 5.48-10.8 | [26] |
|  |  |  |  | <LOQ-6.59 | [20] |  |  |
|  | CPFX | ND-16 | [21] | 0.03-0.09 | [22] | ND-6.28 | [26] |
|  |  | LOQ-12.5 | [28] | 12.8-99.5 | [25] | 0-1,409 | [27] |
|  |  | ND-6.86 | [23] | <LOQ-11.12 | [20] |  |  |
|  | OFX | ND-5.7 | [21] | 0-0.03 | [22] | 0-326 | [27] |
|  |  | ND-19.3 | [23] | 6.72–24.5 | [25] |  |  |
|  |  | LOQ-22.78 | [28] | <LOQ-16.02 | [20] |  |  |
|  | NOR | ND-52 | [21] | 0.05-0.18 | [22] | ND-4.07 | [26] |
|  |  | <LOQ | [28] | 4.16–48.6 | [25] |  |  |
|  |  | ND-8.29 | [23] |  |  |  |  |
|  | ENR | ND-1.4 | [21] | 0-0.04 | [22] | ND-1.27 | [26] |
|  |  | <LOQ | [28] | 6.88–43.6 | [25] | 0-240.50 | [27] |
|  |  |  |  | <LOQ-90.87 | [20] |  |  |
|  | AMP | 3.93-54.2 | [23] |  |  |  |  |
|  | LIN | <LOQ-81.34 | [28] |  |  | 0-541.2 | [27] |
|  | CLI | <LOQ-216.38 | [28] | ND-6.42 | [25] | 0-128.2 | [27] |
| NSAIDs | IBF | ND-18.95 | [24] | ND-130.58 | [24] |  |  |
|  | KPF | 3.47-531.43 | [24] | ND-1,715.4 | [24] |  |  |
|  | NPX | ND-33.99 | [24] | ND-1,070.4 | [24] |  |  |
|  | IM | ND-8.3 | [24] | ND-197.12 | [24] |  |  |


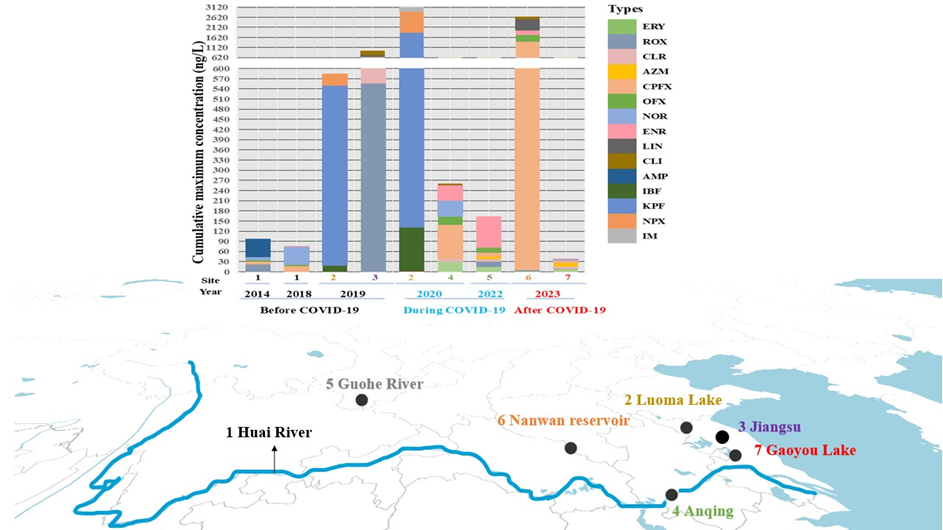


**Fig. S2** Cumulative maximum residue concentrations of anti-COVID-19 drugs collected at representative sampling point in the HuRB.

**Spatial-temporal variations in the occurrence of anti-COVID-19 drugs in the LRB and SRB**

Both the LRB and SRB are located in northeast China, where the usages of pharmaceuticals have been found to be significantly lower than those of the other five regions in China (Eastern, Northern, Central, Southern, and Southwestern parts) [29].

The LRB with a basin area of 2.3 × 10^5^ km^2^ covers Inner Mongolia and Liaoning Province, and supports a population of greater than 4,400,000. Among the seven major rivers in China, the Liao River with total length of 1,430 km has the lowest river flow rate (302 m^3^/s) and annual precipitation (426 mm), thus resulting in limited transportation and higher concentration of pharmaceuticals in the water environment of the LRB [29,30]. The 2017 Chinese Environmental Status Bulletin identified the major tributaries of the Liao River as heavily polluted [29]. However, as shown in Table S6, occurrence data on anti-COVID-10 drugs in the LRB were most limited among seven major river systems in China, with only two studies [22,31] reporting residual levels of 8 antibacterials in LRB’s water environment. Guo et al. [31] collected water samples from 29 sites evenly distributed across the LRB in Liaoning Province. Among the antibacterials commonly used for COVID-19 treatment, the β-L AMX had the highest detection rate (72.41%), with pollution levels of ND-128.59 ng/L, followed by OFX (27.59%, ND-27.21 ng/L). Risk assessment indicated that AMX and OFX in the LRB’s water environment posed high ecological risks to exposed aquatic organisms [31]. Another study conducted during March to July 2020 measured residual concentrations of the antibacterials ERY, ROX, AZM, CPFX, OFX, NOR, ENR in the Dahuofang reservoir, the primary drinking water source for nine cities within the LRB [22]. Results showed that CPFX and NOR were not detected, and the levels of other antibacterials used for COVID-19 treatment were all below 10 ng/L. However, because the Dahuofang reservoir was not included as a sampling site in the pre-COVID-19 study [31], direct comparisons of antibacterial residues in the LRB before and during the pandemic are not feasible based on these limited data.

**Table S6** Occurrence of anti-COVID-19 drugs in surface freshwater samples collected from the LRB and SRB.

| **Basin** | **Group** | **PiE** | **Before the pandemic** | | **During the pandemic** | | **After the pandemic** | |
| --- | --- | --- | --- | --- | --- | --- | --- | --- |
|  |  |  | **Range**(ng/L) | **Reference** | **Range**(ng/L) | **Reference** | **Range** (ng/L) | **Reference** |
| LRB | Antibacterials | ERY |  |  | 2.10-8.90 | [22] |  |  |
|  |  | ROX |  |  | 1.90-9.20 | [22] |  |  |
|  |  | AZM |  |  | 0.90-2.00 | [22] |  |  |
|  |  | CPFX | ND-5.02 | [31] | ND | [22] |  |  |
|  |  | OFX | ND-27.21 | [31] | 0.00-1.80 | [22] |  |  |
|  |  | NOR | ND-13.65 | [31] | ND | [22] |  |  |
|  |  | ENR | ND-5.47 | [31] | 0.00-2.60 | [22] |  |  |
|  |  | AMX | ND-128.59 | [31] |  |  |  |  |
| SRB | Antibacterials | ERY |  |  | 1.2-4.2 | [22] | 7.57-141.22 | [32] |
|  |  |  |  |  | 1.70-2.50 | [22] |  |  |
|  |  |  |  |  | 6.5-113.30 | [22] |  |  |
|  |  | ROX | 0.2-11.5 | [33] | 0.9-2.5 | [22] | 20.87-153.82 | [32] |
|  |  |  | ND-37.3 | [34] | 0.8-1.2 | [22] |  |  |
|  |  |  |  |  | 5.77-143.50 | [22] |  |  |
|  |  | CLR | 0.06-5.14 | [33] |  |  |  |  |
|  |  | AZM | ND-4.17 | [33] | 0.8-2 | [22] |  |  |
|  |  |  |  |  | 1.0-3.9 | [22] |  |  |
|  |  | CPFX | ND-35.5 | [34] | ND | [22] | 6.95-54.56 | [32] |
|  |  |  |  |  | ND | [22] |  |  |
|  |  |  |  |  | 24.27-159.38 | [22] |  |  |
|  |  | OFX | 0.01-1.8 | [33] | ND | [22] | 0.48-9.69 | [32] |
|  |  |  | <LOQ-26.2 | [34] | 0-1.7 | [22] |  |  |
|  |  |  |  |  | 1.74-39.8 | [22] |  |  |
|  |  | NOR | ND-2.4 | [33] | ND | [22] |  |  |
|  |  |  | ND-93.4 | [34] |  |  |  |  |
|  |  | ENR |  |  | ND | [22] |  |  |
|  |  | CTX | ND-5.25 | [33] |  |  |  |  |
|  |  | AMP |  |  | 0.77-6.44 | [32] | 0.57-6.88 | [32] |
|  |  | AMX | 15.9-134 | [34] | 58.12-117.76 | [32] | 56.1-57.29 | [32] |
|  |  | LIN | ND-84.4 | [34] |  |  |  |  |
|  | NSAIDs | IBF | 2.2-53.8 | [34] |  |  |  |  |
|  |  | DFC | 1.2-45 | [34] |  |  |  |  |
|  |  | ATP | ND-65.8 | [34] |  |  |  |  |
|  |  | NPX | 0.4-15.4 | [34] |  |  |  |  |

As the third-longest river in China, the Songhua River with the length of 1,927 km drains a basin of approximately 54.4 km^2^, accounting for over 60% of the total area of northeast China [29,33]. As shown in Table S6, among the included anti-COVID-19 drugs, only AMX was detected in water samples collected from the SRB at concentrations exceeding 100 ng/L before the pandemic. During the pandemic, however, the residual concentrations of four antibacterials, including ERY, ROX, CPFX, and AMX, all exceeded 100ng/L. In April 2023 (post-pandemic), a study covering 18 sampling sites in the SRB, including the Songhua River main stream, the Yitong River, and the Liao River [32], found that ERY and ROX persisted at concentrations up to 141.22 and 153.82 ng/L, respectively, which were slightly higher than during the pandemic reported in the same study [32]. In contrast, this study showed the maximum residual levels of CPFX, OFX, and AMX after the pandemic were only 34.2, 24.3, and 48.6 % of those during the pandemic, respectively [32]. These changes suggest a shift in the usage profile of common drugs used for COVID-19 control.

**References**

1. Tang W, Zhao Y, Wang C, Shan B, Cui J. Heavy metal contamination of overlying waters and bed sediments of Haihe Basin in China. Ecotoxicol Environ Saf. 2013;98:317–23.
2. Lei K, Zhu Y, Chen W, Pan HY, Cao YX, Zhang X, Guo BB, Sweetman A, Lin CY, Ouyang W, He MC, Liu XT. Spatial and seasonal variations of antibiotics in river waters in the Haihe River Catchment in China and ecotoxicological risk assessment. Environ Int. 2019;130:104919.
3. Huangfu Y, Li Q, Yang W, Bu Q, Yang L, Tang J, Gan J. Occurrence, Source Apportionment, and Ecological Risk of Typical Pharmaceuticals in Surface Waters of Beijing, China. Toxics. 2024;12(3):171.
4. Chen M, Hong Y, Jin X, Guo C, Zhao X, Liu N, Lu H, Liu Y, Xu J. Ranking the risks of eighty pharmaceuticals in surface water of a megacity: A multilevel optimization strategy.  Sci Total Environ. 2023;878:163184.
5. Wu Y, Song S, Chen X, Shi Y, Cui H, Liu Y, Yang S. Source-specific ecological risks and critical source identification of PPCPs in surface water: Comparing urban and rural areas. Sci Total Environ. 2023;854:158792.
6. Ma R, Wang B, Yin L, Zhang Y, Deng S, Huang J, Wang Y, Yu G. Characterization of pharmaceutically active compounds in Beijing, China: Occurrence pattern, spatiotemporal distribution and its environmental implication. J Hazard Mater. 2017;323(Pt A):147-55.
7. Li W, Gao L, Shi Y, Liu J, Cai Y. Occurrence, distribution and risks of antibiotics in urban surface water in Beijing, China. Environ Sci Process Impacts. 2015;17(9):1611-9.
8. Duan L, Zhang Y, Wang B, Cagnetta G, Deng S, Huang J, Wang Y, Yu G. Characteristics of pharmaceutically active compounds in surface water in Beijing, China: Occurrence, spatial distribution and biennial variation from 2013 to 2017.  Environ Pollut. 2020;264:114753.
9. Meng Y, Zhang J, Fiedler H, Liu W, Pan T, Cao Z, Zhang T. Influence of land use type and urbanization level on the distribution of pharmaceuticals and personal care products and risk assessment in Beiyun River, China. Chemosphere. 2022;287(Pt 1):132075.
10. Huangfu Y, Shi Y, Yang W, Chang G, Li Q, Gao X, et al. Exploring the occurrence, spatial distribution, and ecological risk of pharmaceuticals in surface water from an urban catchment of Beijing based on DGT in situ measurement. ACS ES&T Water. 2025;5:3205–3215.
11. Zhang Y, Chen H, Jing L, Teng Y. Ecotoxicological risk assessment and source apportionment of antibiotics in the waters and sediments of a peri-urban river. Sci Total Environ. 2020;731:139128.
12. Zhang P, Zhou H, Li K, Zhao X, Liu Q, Li D, Zhao G, Wang L. Occurrence of pharmaceuticals and personal care products, and their associated environmental risks in Guanting Reservoir and its upstream rivers in north China. RSC Adv. 2018;8(9):4703-12.
13. Zhang P, Zhou H, Li K, Zhao X, Liu Q, Li D, Zhao G. Occurrence of pharmaceuticals and personal care products, and their associated environmental risks in a large shallow lake in north China. Environ Geochem Health. 2018;40(4):1525-39.
14. Liu X, Zhang G, Liu Y, Lu S, Qin P, Guo X, Bi B, Wang L, Xi B, Wu F, Wang W, Zhang T. Occurrence and fate of antibiotics and antibiotic resistance genes in typical urban water of Beijing, China.  Environ Pollut. 2019;246:163–73.
15. Yang L, Wang T, Zhou Y, Shi B, Bi R, Meng J. Contamination, source and potential risks of pharmaceuticals and personal products (PPCPs) in Baiyangdian Basin, an intensive human intervention area, China. Sci Total Environ. 2021;760:144080.
16. Fu C, Xu B, Chen H, Zhao X, Li G, Zheng Y, Qiu W, Zheng C, Duan L, Wang W. Occurrence and distribution of antibiotics in groundwater, surface water, and sediment in Xiong'an New Area, China, and their relationship with antibiotic resistance genes. Sci Total Environ. 2022;807(Pt 2):151011.
17. Fang L, Wang L, Chen W, Sun J, Wang L. Identifying the impacts of natural and human factors on ecosystem service in the yangtze and yellow river basins. J Clean Prod. 2021;314:127995.
18. Zhang L, Shen L, Qin S, Cui J, Liu Y. Quinolones antibiotics in the Baiyangdian Lake, China: Occurrence, distribution, predicted no-effect concentrations (PNECs) and ecological risks by three methods. Environ Pollut. 2020;256:113458.
19. Tai H, Yi X, Chai N, Xiao J. Hydrochemical characterization and assessment of health risks of trace elements in the Huai River Basin of China. Environ Sci Pollut Res Int. 2024;31(20):28982–96.
20. Wu H, Liu R, Liu G, He M, Arif M, Li F, Si W, Yue Z, Hu H. Unveiling antibiotic contamination in surface water: A study of the Huaihe River Basin's Huaibei Plain, a significant Chinese herbal medicine planting region. Sci Total Environ. 2024;933:173125.
21. Wu X, Yuan Z, Wang S. Prioritization, sources, and ecological risk of typical antibiotics in the Huai River, a Chinese major river: a warning about aquaculture. Environ Sci Pollut Res Int. 2023;30(23):64254–64.
22. Qadeer A, Rui G, Yaqing L, Ran D, Liu C, Jing D, Anis M, Liu M, Wang S, Jiang X, Zhao X. A mega study of antibiotics contamination in Eastern aquatic ecosystems of China: occurrence, interphase transfer processes, ecotoxicological risks, and source modeling. J Hazard Mater. 2023;458:131980.
23. Feng J, Liu Q, Ru X, Xi N, Sun J. Occurrence and distribution of priority pharmaceuticals in the Yellow River and the Huai River in Henan, China. Environ. Sci Pollut Res Int. 2020;27(14):16816–26.
24. Wang N, Kang G, Hu G, Chen J, Qi D, Bi F, Chang N, Gao Z, Zhang S, Shen W. Spatiotemporal distribution and ecological risk assessment of pharmaceuticals and personal care products (PPCPs) from Luoma Lake, an important node of the South-to-North Water Diversion Project. Environ Monit Assess. 2023;195(11):1330.
25. Chen H, Zheng W, Shen X, Zhang F, Zhou X, Shen J, Lu M. Occurrence, Distribution, and Ecological Risk Assessment of Antibiotics in Different Environmental Media in Anqing, Anhui Province, China. Int J Environ Res Public Health. 2021;18(15):8112.
26. Xu X, Yang C, Zou L, Leng J, Wang N, Zhang J. Occurrence, distribution, and ecological risks of antibiotics and antibiotic resistance genes in the surface waters of Gaoyou Lake, China. Environ Monit Assess. 2024;196(10):967.
27. Zhao J, Hou S, Zhang H, Sun S, Guo C, Zhang X, Song G, Xu J. Spatiotemporal variations and priority ranking of emerging contaminants in nanwan reservoir: A case study from the agricultural region in huaihe river basin in China. J Environ Manage. 2024;368:122195.
28. Kong M, Bu YQ, Zhang Q, Zhang SH, Xing LQ, Gao ZQ, Bi FZ, Hu GJ. Distribution, abundance, and risk assessment of selected antibiotics in a shallow freshwater body used for drinking water, China. J Environ Manage. 2021;280:111738.
29. Li Y, Ding J, Zhang L, Liu X, Wang G. Occurrence and ranking of pharmaceuticals in the major rivers of China. Sci Total Environ. 2019;696:133991.
30. Xu Y, Guo C, Lv J, Hou S, Luo Y, Zhang Y, Xu J. Spatiotemporal profile of tetracycline and sulfonamide and their resistance on a catchment scale. Environ Pollut. 2018;241:1098–105.
31. Guo H, Zhao F, Li R, Jin SC, Zhang HB, Zhang KY, Li SS, Shu Q, Na GS. Occurrence and distribution of antibiotics and antibiotic resistance genes in water of Liaohe River Basin, China. J Environ Chem Eng. 2022;10(5):108297.
32. Chen A, Zhang T, Cheng F, Yang H, Guo Z, Zhao S, Zhang YN, Qu J. Comprehensive analysis and risk assessment of Antibiotic contaminants, antibiotic-resistant bacteria, and resistance genes: Patterns, drivers, and implications in the Songliao Basin. Environ Pollut. 2024;361:124852.
33. Wang W, Wang H, Zhang W, Liang H, Gao D. Occurrence, distribution, and risk assessment of antibiotics in the Songhua River in China. Environ Sci Pollut Res Int. 2017;24(23):19282–92.
34. Zhang L, Du S, Zhang X, Lyu G, Dong D, Hua X, Zhang W, Guo Z. Occurrence, distribution, and ecological risk of pharmaceuticals in a seasonally ice-sealed river: From ice formation to melting.  J Hazard Mater. 2020;389:122083.
